# IL4I1⁺ Macrophages and TDO2⁺ Myofibroblasts Drive AhR‐Mediated Immunosuppression and Ferroptosis Resistance in Solid Predominant Lung Adenocarcinoma

**DOI:** 10.1002/advs.202513606

**Published:** 2025-12-22

**Authors:** Zhaoxuan Wang, Weijiao Xu, Lei Zhao, Lin Zhong, Wendan Yu, Shengmin Wang, Lu Sun, Tao Guo, Fengzhou Li, Zhuoshi Li, Lei Fang, Shiqing Wang, Guohui Zhang, Guoqing Xue, Wei Guo, Shilei Zhao, Chundong Gu

**Affiliations:** ^1^ Department of Thoracic Surgery The First Affiliated Hospital of Dalian Medical University Dalian China; ^2^ Department of Thoracic Surgery Shanghai Chest Hospital Shanghai Jiao Tong University School of Medicine Shanghai Key Laboratory of Thoracic Tumor Biotherapy Shanghai China; ^3^ Institute of Cancer Stem Cell Dalian Medical University Dalian China; ^4^ Department of Pathology The First Affiliated Hospital of Dalian Medical University Dalian China; ^5^ Department of Oncology The Second Hospital of Dalian Medical University Dalian China

**Keywords:** cancer‐associated fibroblasts, ferroptosis, histological subtypes, lung adenocarcinoma, tumor microenvironment, tumor‐associated macrophages

## Abstract

Lung adenocarcinoma (LUAD) displays marked intratumoral heterogeneity with distinct histological patterns. The solid pattern representing poorly differentiated LUAD is linked to poor prognosis and therapeutic resistance. To uncover underlying mechanisms, we integrate bulk and single‐cell RNA sequencing and identify a preferential enrichment of interleukin 4 induced 1 (IL4I1)‐expressing tumor‐associated macrophages (TAMs) and tryptophan 2,3‐dioxygenase (TDO2)‐expressing myofibroblastic cancer‐associated fibroblasts (myCAFs) in a solid pattern of LUAD. Spatial transcriptomics reveals their co‐localization in peritumoral stroma, forming an immune‐excluded niche. Mechanistically, TDO2⁺ myCAFs promoted monocyte‐to‐IL4I1⁺ TAM differentiation via the kynurenine‐aryl hydrocarbon receptor (AhR) axis. Tryptophan metabolomic landscapes confirm that IL4I1⁺ TAMs and TDO2⁺ myCAFs enhance tryptophan degradation and accumulation of AhR ligands (e.g., kynurenine, indole‐3‐carboxaldehyde), contributing to CD8⁺ T cell exhaustion and anti‐PD‐1 therapeutic resistance. IL4I1⁺ TAMs and TDO2⁺ myCAFs conformably mediate ferroptosis resistance through the AhR‐NRF2‐GPX4‐SLC7A11 pathway. Notably, AhR antagonist CH‐223191 restores ferroptosis sensitivity of tumor cells. A triple therapy combining CH‐223191, ferroptosis inducer (Imidazole ketone erastin or RSL3), and anti‐PD‐1 agent demonstrates superior efficacy and safety in vivo. Together, our findings demonstrate that IL4I1⁺ TAMs and TDO2⁺ myCAFs synergistically establish an immunosuppressive, ferroptosis‐resistant niche via AhR signaling in solid predominant LUAD and offer promising therapeutic strategies to reprogram the tumor microenvironment.

## Graphical abstract

Solid predominant lung adenocarcinoma exhibits an immune‐excluded, ferroptosis‐resistant niche enriched with IL4I1⁺ TAMs and TDO2⁺ myCAFs. Spatial and multi‐omics analyses reveal AhR‐driven crosstalk that promotes T cell exhaustion and therapy resistance. Blocking AhR with CH‐223191 restores ferroptosis sensitivity, and its combination with ferroptosis inducers and anti‐PD‐1 achieves potent antitumor efficacy.

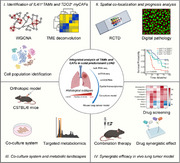

## 1 Introduction

Lung adenocarcinoma (LUAD), as a malignancy with a significant mortality burden, is marked by histological heterogeneity and dynamic cellular plasticity [1]. Clinically, LUAD progression typically follows an evolutionary sequence from adenocarcinoma in situ (AIS) to minimally invasive adenocarcinoma (MIA), ultimately developing into invasive adenocarcinoma (IAC) [2]. Upon progression to the IAC stage, LUAD demonstrates distinctive histopathological heterogeneity, which significantly complicates its diagnosis and clinical management. Consensual diagnostic criteria according to the latest WHO classification of LUAD generally recognize distinct histological growth patterns, including lepidic (well differentiated), papillary/acinar (moderately differentiated), and micropapillary/solid (poorly differentiated), with tumor specimens frequently demonstrating coexisting patterns along this differentiation spectrum [3]. Emerging evidence has established strong correlations between histological subtypes and clinical outcomes [4], particularly regarding therapeutic responses to chemotherapy [5], epidermal growth factor receptor tyrosine kinase inhibitors (EGFR‐TKIs) targeted therapy [6], and immune checkpoint inhibitors (ICIs) therapy [7]. Notably, the solid predominant subtype, representing the most aggressive high‐grade pattern, serves as an independent prognostic indicator for poor survival outcomes, particularly pronounced in early‐stage LUAD patients [8]. Additionally, this subtype demonstrates distinct immunological characteristics, including dense tumor‐infiltrating lymphocytes (TILs) populations and elevated expression of immunosuppressive checkpoints, including programmed death‐ligand 1 (PD‐L1) and lymphocyte‐activating 3 (LAG3) [9]. These findings underscore the need for comprehensive investigations into the molecular drivers of histological progression in LUAD, particularly mechanisms governing pattern‐specific tumor microenvironment (TME) remodeling and therapeutic resistance development.

Previous studies have elucidated the multi‐omics landscape underlying histological heterogeneity in LUAD. Genomic evolution is recognized as a crucial oncogenic driver, with the TRACERx study [10], which demonstrated that tumors dominated by high‐grade patterns (e.g., solid and micropapillary) exhibit elevated tumor mutational burden (TMB), higher rates of whole‐genome doubling (WGD), and recurrent somatic alterations in oncogenic driver genes such as *TP53*, *KRAS*, and *SMARCA4*. Concurrently, dynamic TME remodeling significantly contributes to histological evolution [11]. The TME encompasses diverse cellular components, including immune cells (e.g., T cells, B cells, and tumor‐associated macrophages [TAMs]) [12], stromal cells (e.g., cancer‐associated fibroblasts [CAFs] and endothelial cells) [13], and non‐cellular elements such as extracellular matrix (ECM) proteins, cytokines, chemokines, metabolites, and extracellular vesicles [14]. Large‐scale single‐cell spatial analyses in LUAD have revealed that solid predominant LUAD displays the most significant immune infiltration across histological subtypes, characterized by dense TILs and myeloid cell dominance [15]. In particular, CD163^+^ TAMs emerge as pivotal mediators of immunosuppression and tumor progression in this aggressive subtype. In addition to immune cells, CAFs serve as pivotal regulators in the TME, which drive ECM remodeling, establish pro‐metastatic niches, and suppress anti‐tumor immunity through crosstalk with immune cells [16]. Recent research has further uncovered remarkable functional heterogeneity within the CAFs population, including myofibroblastic CAFs (myCAFs), which promote ECM stiffness and metastasis; inflammatory CAFs (iCAFs), which secrete cytokines to sustain chronic inflammation; and antigen‐presenting CAFs (apCAFs), implicated in modulating T cell responses [17]. Strikingly, CAFs‐derived paracrine transforming growth factor‐β (TGF‐β) signaling has been shown to induce phenotypic plasticity in tumor cells, driving transitions from solid to acinar growth patterns [18]. Moreover, recent findings highlight that bidirectional interactions between TAMs and CAFs orchestrate critical processes in tumor initiation [19], metastasis [20], and therapeutic resistance [21], with these cellular alliances increasingly implicated in modulating response to chemotherapy, targeted therapies, and immunotherapies [22]. Despite these advances, mechanistic associations between histological subtype‐specific TME and clinical outcomes such as survival disparities and treatment responses remain poorly defined. Over the past decade, single‐cell RNA sequencing (scRNA‐seq) and spatial transcriptomics have revolutionized TME profiling, enabling high‐resolution dissection of cellular subpopulations, intercellular communication networks, and context‐dependent phenotypic plasticity. These technologies now provide unprecedented opportunities to decode how stromal‐immune‐tumor cell interactions drive histological diversification and therapeutic vulnerabilities in LUAD.

In the study, we performed a multi‐omics investigation in solid predominant LUAD through integrated analysis of bulk RNA‐seq, scRNA‐seq, tissue microarray, spatial transcriptomics, and multiplexed immunohistochemistry (IHC). Our multi‐platform approach revealed a characteristic enrichment of IL4I1^+^ TAMs and TDO2^+^ myCAFs within the peritumoral region of the solid pattern. Subsequent co‐culture experiments revealed that TDO2^+^ myCAFs‐derived kynurenine (Kyn) promoted IL4I1 expression in macrophages through the aryl hydrocarbon receptor (AhR) binding promoter region of IL4I1. Extensive AhR transcriptional activation in the TME of solid predominant LUAD synergistically exhausted CD8^+^ T cell cytotoxicity, conferred anti‐PD‐1 therapy resistance, and induced tumor cells ferroptosis resistance through NRF2‐GPX4‐SLC7A11 pathway. Notably, we developed a novel combinatorial therapeutic strategy targeting these pathways consisting of AhR antagonist CH‐223191, ferroptosis inducer (Imidazole ketone erastin [IKE] or RSL3), and anti‐PD‐1 therapy, which demonstrated potent antitumor efficacy with an acceptable safety profile. These findings provide both mechanistic insights into the TME of solid predominant LUAD regulation and a precision medicine framework for tailored LUAD treatment based on predominant growth patterns.

## 2 Results

### 2.1 Bulk RNA‐Seq Identifies Significant Enrichments of TAMs and CAFs in Solid Predominant LUAD

**FIGURE 1:**
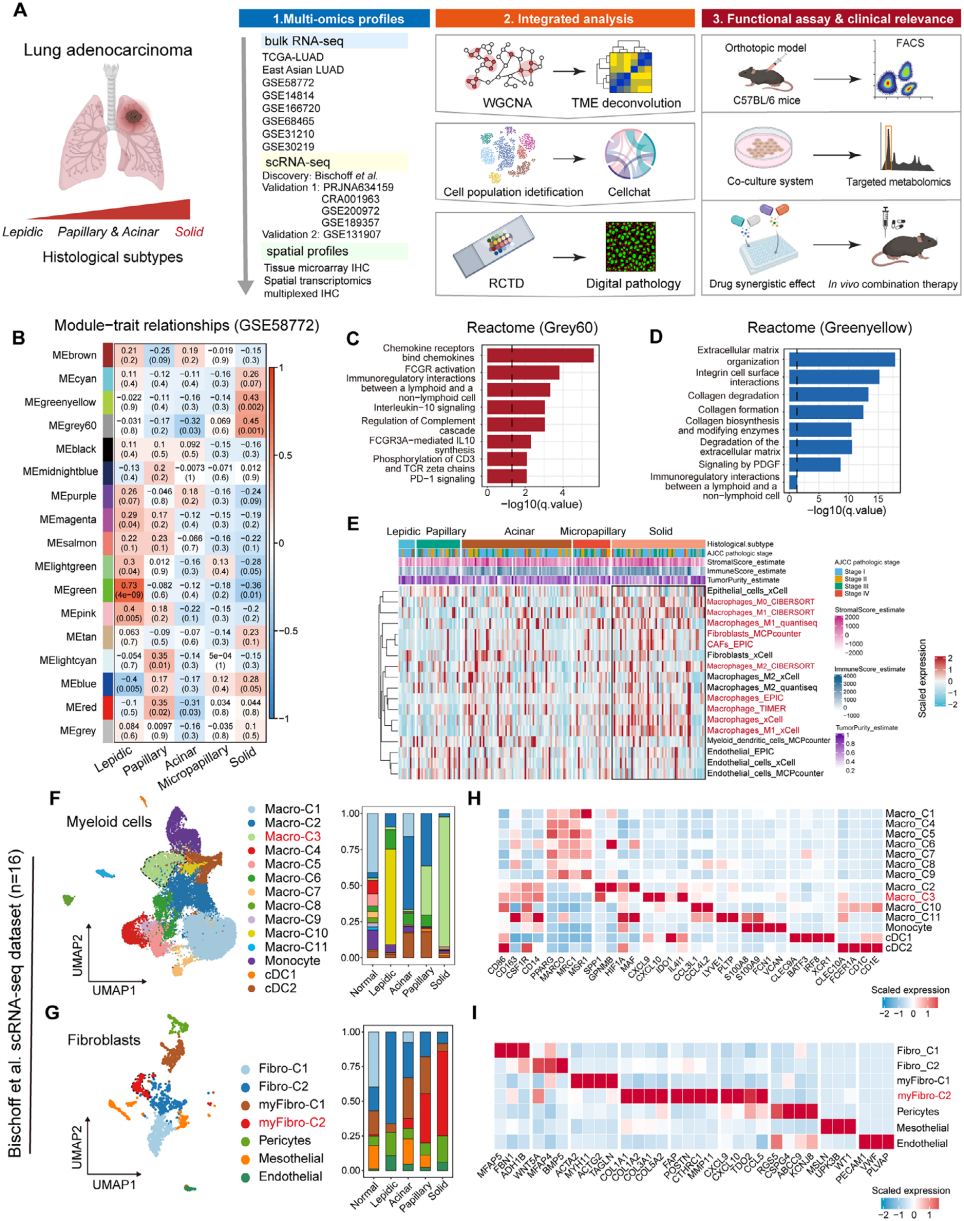
Bulk RNA‐seq and scRNA‐seq analyses reveal TAMs and CAFs in solid predominant LUAD. **(A)** Overall study workflow. **(B)** Consensus network modules correlated with histological subtypes in the GSE58772 cohort (*n* = 48) using the eigenmodule (the first principal component of the module). Pearson correlation coefficient, along with *p*‐value in parentheses underneath; color‐coded according to correlation coefficient. The blue color indicates a negative correlation, while the red color represents a positive correlation. (**C,D)** Reactome pathway enrichment analyses of genes in greenyellow **(C)** and grey60 modules **(D)**. (**E) **Heatmap showing the immune and stroma infiltration in the TME among histologic subtypes in TCGA‐LUAD cohort (*n* = 186). (**F)** UMAP plot showing sub‐clustering of myeloid cells from Bischoff et al.’s study (*n* = 16), colored and labeled by cell subtypes (left), and stacked histogram showing the frequency of subclusters of myeloid cells among histologic subtypes (right). (**G)** UMAP plot showing sub‐clustering of fibroblasts from Bischoff et al.’s study (*n* = 16), colored and labeled by subtypes (left), and stacked histogram showing the frequency of subclusters of fibroblasts among histologic subtypes (right). (**H)** Heatmap showing the expression levels of specific markers among myeloid cells subclusters. **(I)** Heatmap showing the expression levels of specific markers among fibroblasts subclusters.

The overall design of this study is shown in Figure 1A. To systematically elucidate critical properties of solid predominant LUAD, we first performed weighted gene co‐expression network analysis (WGCNA) [23] on the microarray dataset GSE58772 [24]. Specifically, the dataset was generated using laser capture microdissection to separate tumor regions that precisely represent distinct histological patterns. The whole‐transcriptome containing 34,694 genes and 48 tumor samples (lepidic, *n* = 10; papillary, *n* = 9; acinar, *n* = 10; micropapillary, *n* = 9; solid, *n* = 10) was clustered into 17 co‐expression modules (Figure S1A–C and Table S1). We focused on grey60 module (*n* = 101, *p* = 0.001) and the greenyellow module (*n* = 241, *p* = 0.002), which were not only significantly positively correlated with solid phenotype but also showed a moderate inter‐module correlation (Figure 1B; Figure S1D). Then, we conducted protein‐protein interaction (PPI) network using STRING database and Cytohubba [25] to identify hub elements in each module: grey60 module featured immune‐related chemokines and receptors (*CXCL9*, *CXCL10*, *CXCL13*, *CD163*, *CSF1R*) and greenyellow module showed an enrichment in extracellular matrix (ECM) components (*COL5A2*, *THBS2*, *COL4A2*, *COL6A2*, *COL1A1*) (Figure S1E). Complementary analysis of module membership (MM) and gene significance (GS) validated these hub gene selections (Figure S1F,G). Subsequently, functional enrichment analysis [26, 27] of grey60 module showed enrichment in several immune‐related processes such as chemokine receptors and chemokines, Fc‐gamma receptors (FCGR) activation, interleukin‐10 signaling, and PD‐1 signaling (Figure 1C). In addition, the greenyellow module demonstrated activation of ECM organization, collagen degradation, and signaling by platelet‐derived growth factor (PDGF) (Figure 1D). In previous studies, these molecular signatures respectively represented established markers of TAMs [28] and myCAFs [29]. To validate these findings, we analyzed cell compositions of TME in TCGA‐LUAD cohort with confirmed histologic subtypes using seven widely adopted algorithms [30], including ESTIMATE [31], CIBERSORT [32], EPIC [33], xCell [34], MCP‐counter [35], TIMER [36], and quantiseq [35]. In line with WGCNA findings, the ESTIMATE algorithm revealed that solid predominant LUAD significantly exhibited higher immune and stromal scores compared to other histological subtypes. Furthermore, solid predominant LUAD samples exhibited an increased abundance of macrophages (both M1‐like macrophages and M2‐like macrophages), fibroblasts, and CAFs (Figure 1E). These findings suggested TAMs and CAFs as crucial phenotypes in the solid pattern of LUAD.

### 2.2 Single‐Cell Transcriptomic Profiling Reveals Distinct Immunosuppressive TAMs and myCAFs Subclusters in Solid Predominant LUAD

To elucidate the heterogeneity of TAMs and CAFs, we first analyzed a discovery scRNA‐seq dataset from Bischoff et al.’s study [37]. We selected eight tumor samples (lepidic, *n* = 1; papillary, *n* = 1; acinar, *n* = 4; solid, *n* = 2) and eight matched normal lung tissues for further analysis. Following quality control and batch correction procedures [38, 39], we obtained a sparse matrix comprising 86 275 cells and 27 854 genes (Figure S2A,B). Using typical cell markers, total cells were classified into nine major cell types: epithelial cells (*EPCAM*, *CDH5*, *KRT7*, *KRT18*, *n* = 14,241), T/NK cells (*PTPRC*, *CD3D*, *CD3E*, *NKG7*, *GNLY*, *n* = 27 954), myeloid cells (*S100A8*, *S100A9*, *CSF1R*, *CD163*, *CD86*, *n* = 31 992), B cells (*CD79A*, *CD19*, *MS4A1*, *n* = 2228), plasma cells (*CD79A*, *JCHAIN*, *IGKC*, *n* = 2,505), proliferating immune cells (*PTPRC*, *MKI67*, *TOP2A*, *n* = 1,841), mast cells (*TPSB2*, *KIT*, *CPA3*, *n* = 1,667), endothelial cells (*PECAM1*, *VWF*, *n* = 2,141), and fibroblasts (*COL1A1*, *COL1A2*, *ACTA2*, *n* = 1,706) (Figure S2C,D) [40]. Consistent with findings of RNA‐seq, solid predominant LUAD samples exhibited a greater proportion of immune and stromal cells and a lower proportion of epithelial cells (Figure S2E). Then, we respectively performed an in‐depth analysis of myeloid cells and fibroblasts, referring to specific signatures proposed by previous studies. Briefly, myeloid cells were clustered into monocytes, macrophages (Macro‐C1 to Macro‐11), and conventional dendritic cells (cDC1 and cDC2) (Figure 1F). Fibroblasts were clustered into fibroblasts (Fibro‐C1 and Fibro‐C2), myofibroblasts (myFibro‐C1 and myFibro‐C2), pericytes, mesothelial cells, and endothelial cells (Figure 1G). Representative marker genes were shown in Figure 1H,I. Analysis of cellular subpopulation proportions across histological subtypes revealed that resident‐tissue macrophages (*PPARG*, *MACRO*, *MRC1*, and *MSR1*) and Fibro‐C1 (*MFAP5*, *FBN1*, and *ADH1B*) were majorly distributed in normal lung tissues. Lepidic predominant LUAD samples were primarily composed of inflammatory Macro‐C10 (*CCL3L1* and *CCL4L2*) and Fibro‐C2 (*WNT5A*, *MFAP4*, and *BMP5*). Acinar and papillary predominant LUAD samples featured Macro‐C2 (*SPP1*, *GPNMB*, *HIF1A*, and *MAF*) and myFibro‐C1 (*ACTA2*, *MYH11*, *ACTG2*, and *TAGLN*). Macro‐C3 and myFibro‐C2 respectively, represented dominant myeloid cell and fibroblast subpopulations in solid predominant LUAD (Figure 1F,G). Notably, Macro‐C3 and myFibro‐C2 synergistically displayed overexpression of T cell chemokines (*CXCL9* and *CXCL10*) (Figure 1H,I), which indicated these two subpopulations may possess the ability to recruit CXCR3^+^ T cells in the TME. Besides, myFibro‐C2 was characterized by enriched ECM remodeling markers (e.g., *FAP*, *POSTN*, *COL5A2*, *CTHRC1*, and *MMP11*) (Figure 1I), which may develop a stromal niche for T cells infiltration. Interestingly, these two subpopulations respectively expressed high levels of tryptophan (Trp) metabolizing enzymes (Macro‐C3:*IL4I1* and *IDO1*; myFibro‐C2:*TDO2*). Recent studies revealed that IDO1, TDO2, and IL4I1 as Trp catabolic enzymes respectively converted Trp to Kyn (IDO1/TDO2) [41] and indole‐3‐pyruvic acid (I3P) (IL4I1) [42] in the intra‐ and extracellular milieu, which limited T cells proliferation and induced aggregation of CD8^+^ PD‐1^+^ exhausted T cells (Tex) and CD4^+^ Foxp3^+^ regulatory T cells (Treg) through AhR transcriptional activation [43]. To further substantiate the robustness of these findings, we performed additional analyses using two independent validation cohorts, including validation cohort 1 (PRJNA634159 [44], CRA001963 [45], GSE200972 [46], and GSE189357 [47]) and validation cohort 2 (GSE131907 [48]). The validation cohort 1 comprised 16 tumor samples, including AIS (*n* = 3), MIA (*n* = 3), papillary (*n* = 4), acinar (*n* = 2), and solid (*n* = 4) subtypes. Notably, LUAD at the stage of AIS and MIA grew as a typical lepidic pattern. After performing batch correction and preliminary cell‐type classification (Figure S3A,B), we extracted myeloid and fibroblast subpopulations for subsequent analyses. We identified that Macro‐C2 (*IL4I1*, *IDO1, CXCL9*, *CXCL10*, *IL2RA*, and *MMP2*) and Fibro‐C1 (*FAP*, *POSTN*, *CTHRC1*, *COL1A1, COL5A2*, *TDO2*, *CXCL9*, and *CXCL10*) had higher proportions in solid predominant LUAD samples (Figure S3C–F). Additionally, we evaluated signature scores of Macro‐C3 and myFibro‐C2 based on their respective top 50 genes and found their enrichment levels were prominently elevated in the solid subtype within validation cohort 1 (Figure S3G,H). Moreover, analysis of validation cohort 2 with parallel pipeline yielded consistent results (Figure S3I,J), with Macro‐C1 (*IL4I1*, *IDO1, CXCL9*, *CXCL10*, *IL2RA*, and *CD163*) and Fibro‐C1 (*FAP*, *POSTN*, *CTHRC1*, *COL1A1, COL5A2*, *TDO2*, *CXCL9*, and *CXCL10*) being predominantly enriched in poorly differentiated LUAD samples (Figure S3K–N). The gene signatures of Macro‐C3 and myFibro‐C2 were also upregulated in poorly differentiated LUAD samples, further validating our initial observations (Figure S3O,P).

Furthermore, we observed that signature scores of Macro‐C3 and myFibro‐C2 had a stronger positive correlation with CD8⁺ T cells and Tregs (Figure S4A,B), compared to other immune cells, including B cells, monocytes, CD4⁺ T cells, NK cells, neutrophils, and dendritic cells. Moreover, these scores demonstrated a positive relationship with co‐inhibitory immune checkpoints such as *CD274*, *PDCD1*, *LAG3*, *TIGIT*, *HAVCR2*, *CTLA4*, and *IL2RA* (Figure S4C,D). The result indicated their immunosuppressive roles by restricting T cell function. Then, we confirmed that Macro‐C3 and myFibro‐C2 in the discovery cohort exhibited synergistic enrichment in interleukin‐10 signaling, immunoregulatory interactions between a lymphoid and a non‐lymphoid cell, and Trp catabolism pathway (Figure S5A,B). Thus, we re‐clustered T/NK cells in the scRNA‐seq dataset and observed a larger fraction of exhausted CD8^+^ CXCR3^+^ T cells (CD8^+^ Tex‐PDCD1) and regulatory CD4^+^ CXCR3^+^ T cells (CD4^+^ Treg‐FOXP3) in solid predominant LUAD (Figure S5C,D). Notably, these suppressive T cell subpopulations exhibited higher expression of AhR activating pathway genes, including *AhR*, *HSP90AB1*, *CYP1A2*, and *ARNT* (Figure S5E), suggesting that these T cells may be maintained in a state of persistent AhR activation and suppression regulated by Macro‐C3 and myFibro‐C2. Furthermore, the ligand‐receptor analysis using the CellChat algorithm [49] revealed that Macro‐C3 and myFibro‐C2 orchestrated the higher strength of outgoing interactions from other subpopulations in solid predominant LUAD (Figure S5F,G). Specifically, Macro‐C3 and myFibro‐C2 communicated through ECM‐receptor interactions including COL1A1‐CD44, FN1‐CD44, LAMB1‐CD44, and THBS2‐CD47 and secreted signals including growth factor pathway (TGFB1‐ACVR1/TGFBR1, PDGF‐PDGFR, and HBEGF‐EGFR/ERBB2), as well as cytokines signaling (CSF1‐CSF1R, CCL2‐CCR2, IL1B‐ILR1, MIF‐CD74/CD44, C3‐/ITGAX/ITGB2, and RARRES2‐CMKLR1) (Figure S5H). Besides, Macro‐C3 and myFibro‐C2 could synergistically interact with suppressive T cells through CXCL9/CXCL10‐CXCR3 (Figure S5H). Moreover, a deep learning‐based histopathological analysis by Saltz et al. [50] of H&E‐stained images in TCGA‐LUAD cohort confirmed that the subtype of “brisk, band‐like” tumor‐infiltrating lymphocytes (TILs) exhibited higher signature scores of Macro‐C3 and myFibro‐C2 (Figure S5I–K), suggesting substantial immune infiltration at the tumor margin without effective anti‐tumor activity. Overall, we recognized IL4I1^+^ IDO1^+^ TAMs (Macro‐C3) and TDO2^+^ myCAFs (myFibro‐C2) as the dominant immunosuppressive cell populations in solid predominant LUAD, laying a foundation for investigating their clinical relevance and therapeutic targeting.

### 2.3 Clinical Implications of IL4I1^+^ TAMs and TDO2^+^ myCAFs in LUAD Patients

**FIGURE 2:**
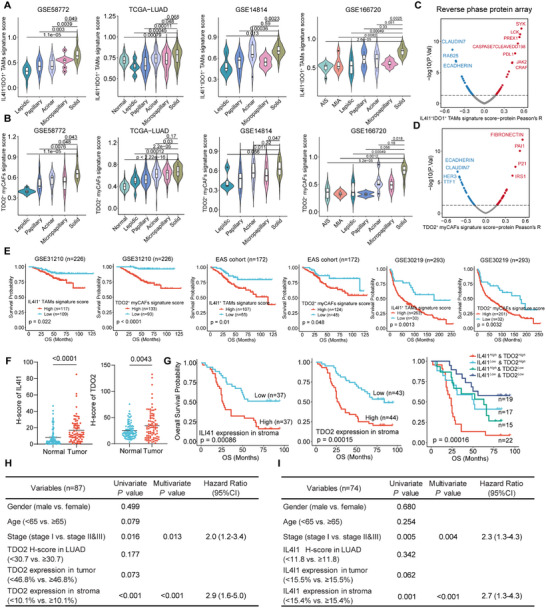
Clinical implications of IL4I1^+^ TAMs and TDO2^+^ myCAFs in LUAD patients. **(A,B)** Violin plot showing signature scores of IL4I1^+^ IDO^+^ TAMs **(A)** and TDO2^+^ myCAFs **(B)** across histological subtypes in GSE58772 (*n* = 48), TCGA‐LUAD (*n* = 245), GSE14814 (*n* = 51), and GSE166720 (*n* = 52). *P*‐values were calculated with Wilcoxon test. **(C,D)** The scatterplot showing the correlation between the signature scores of IL4I1^+^ IDO^+^ TAMs **(C)** and TDO2^+^ myCAFs **(D)** and the protein level in TCGA‐LUAD. The blue color indicates a significantly negative correlation (*p*‐value < 0.05, Peason's *R* < −0.3), while the red color represents a significantly positive correlation (*p* < 0.05, Peason's *R* > 0.3). **(E) **Kaplan–Meier overall survival curves of signature scores of IL4I1^+^ TAMs and TDO2^+^ myCAFs in GSE31210 (*n* = 226), EAS cohort (*n* = 172), and GSE30219 (*n* = 293). *P*‐values were calculated by log‐rank test. (**F)** Quantification of IL4I1 and TDO2 staining intensity in the adjacent normal and LUAD tissue from the tissue microarray. *P*‐values were calculated with Student's *t*‐test. (**G)** Kaplan‐Meier survival curves generated for the positive proportions of IL4I1 in stroma (*n* = 74), TDO2 in stroma (*n* = 87), and both (*n* = 73) from the tissue microarray. Patients were divided into high‐ and low‐expression groups (median‐cutoff). *P*‐values were calculated by log‐rank test. (**H,I)** Univariate and multivariate Cox hazard regression analysis of IL4I1 and TDO2 for OS in LUAD.

Next, we evaluated abundance of IL4I1^+^ IDO1^+^ TAMs and TDO2^+^ myCAFs by calculating signature scores. Solid predominant LUAD samples exhibited significantly higher signature scores of IL4I1^+^ IDO1^+^ TAMs and TDO2^+^ myCAFs compared to normal lung tissues and non‐solid tumor samples in multiple bulk RNA‐seq datasets, including GSE58772 [24] (*n* = 48), TCGA‐LUAD (*n* = 245), GSE14814 [51] (*n* = 51), and GSE166720 [52] (*n* = 52) (Figure 2A,B). In addition, signature scores of IL4I1^+^ IDO1^+^ TAMs and TDO2^+^ myCAFs in GSE68465 [53] (*n* = 462) also exhibited higher levels in LUAD tissues, especially poorly differentiated tumor samples (Figure S6A,B). Besides, reverse phase protein array (RPPA) in TCGA‐LUAD [54] showed that higher signature scores of IL4I1^+^ IDO1^+^ TAMs and TDO2^+^ myCAFs characterized by high protein levels of fibronectin, PD‐L1, and plasminogen activator inhibitor‐1 (PAI‐1) (*p* < 0.05, Pearson's *R* > 0.3), while low levels of typical well‐differentiated LUAD biomarkers including E‐cadherin, HER3, Claudin‐7, and TTF1 (*p* < 0.05, Pearson's *R* < −0.3) (Figure 2C,D). Further survival analyses in multiple LUAD cohorts demonstrated that higher signature scores of IL4I1^+^ IDO1^+^ TAMs and TDO2^+^ myCAFs in LUAD cohorts, including GSE31210 [55] (*n* = 226), East Asian LUAD (EAS) cohort [56] (*n* = 172), and GSE30219 [57] (*n* = 293), were significantly correlated with poor prognosis (Figure 2E; Figure S6C). Multivariate Cox regression confirmed that higher signature scores of IL4I1^+^ IDO1^+^ TAMs and TDO2^+^ myCAFs were independent prognostic risk factors (Figure S6D–G). These results supported that these two cell types especially existed in solid predominant LUAD and further contributed to unfavorable clinical outcomes.

Although both IL4I1 and IDO1 were co‐expressed in TAMs, IL4I1 exhibited more specific and prevalent expression within macrophage populations in scRNA datasets from Bischoff et al.’ s study [37] and EMTAB6149 [58] (Figure 1H; Figure S7A,B). Additionally, IL4I1‐derived metabolites demonstrated a stronger capacity to activate AhR pathway [42]. Therefore, we were particularly interested in IL4I1^+^ TAMs and performed subsequent analyses on their clinical relevance and biological functions. We first assessed positive levels of IL4I1 and TDO2 using a tissue microarray (TMA) from LUAD patients (Figure S7C–F). H‐scores of both IL4I1 and TDO2 were significantly elevated in tumor tissues compared to adjacent normal tissues (Figure 2F). However, higher‐than‐median IL4I1 (H‐score>11.8) or TDO2 (H‐score > 30.7) expression in whole tissues was not associated with OS, suggesting that their prognostic value might be obscured by spatial heterogeneity, in which distinct tumor regions or specific cellular subpopulations contributed differently to disease progression and patient survival. To address this, we trained a machine learning‐based object classifier in QuPath software to identify the tumor region and the stromal region (Figure S7G–I). Kaplan‐Meier analysis revealed that higher IL4I1 expression in stromal region (≥ 15.1%) was significantly associated with poor OS (*p* = 0.00086), whereas IL4I1 expression in the tumor region showed no prognostic value. Similarly, elevated stromal TDO2 expression (≥ 10.1%) correlated with shorter OS (*p* = 0.00015) (Figure 2G). Notably, patients with high stromal expression of both IL4I1 and TDO2 had the worst prognosis (*p* = 0.00016) (Figure 2G). Cox regression analysis further confirmed that, along with TNM stage, stromal IL4I1 and TDO2 expression were independent predictors of OS in both univariate and multivariate models (Figure 2H,I). Collectively, these findings suggested that IL4I1^+^ TAMs and TDO2^+^ myCAFs, as stromal‐enriched populations with heightened Trp‐metabolizing activity, may contribute to the establishment of an immunosuppressive TME in solid predominant LUAD, thereby promoting disease progression and poor prognosis.

### 2.4 Spatial Profiling Reveals IL4I1^+^ TAMs and TDO2^+^ myCAFs Exhibit Co‐Localization and Recruit Suppressor T Cells

**FIGURE 3:**
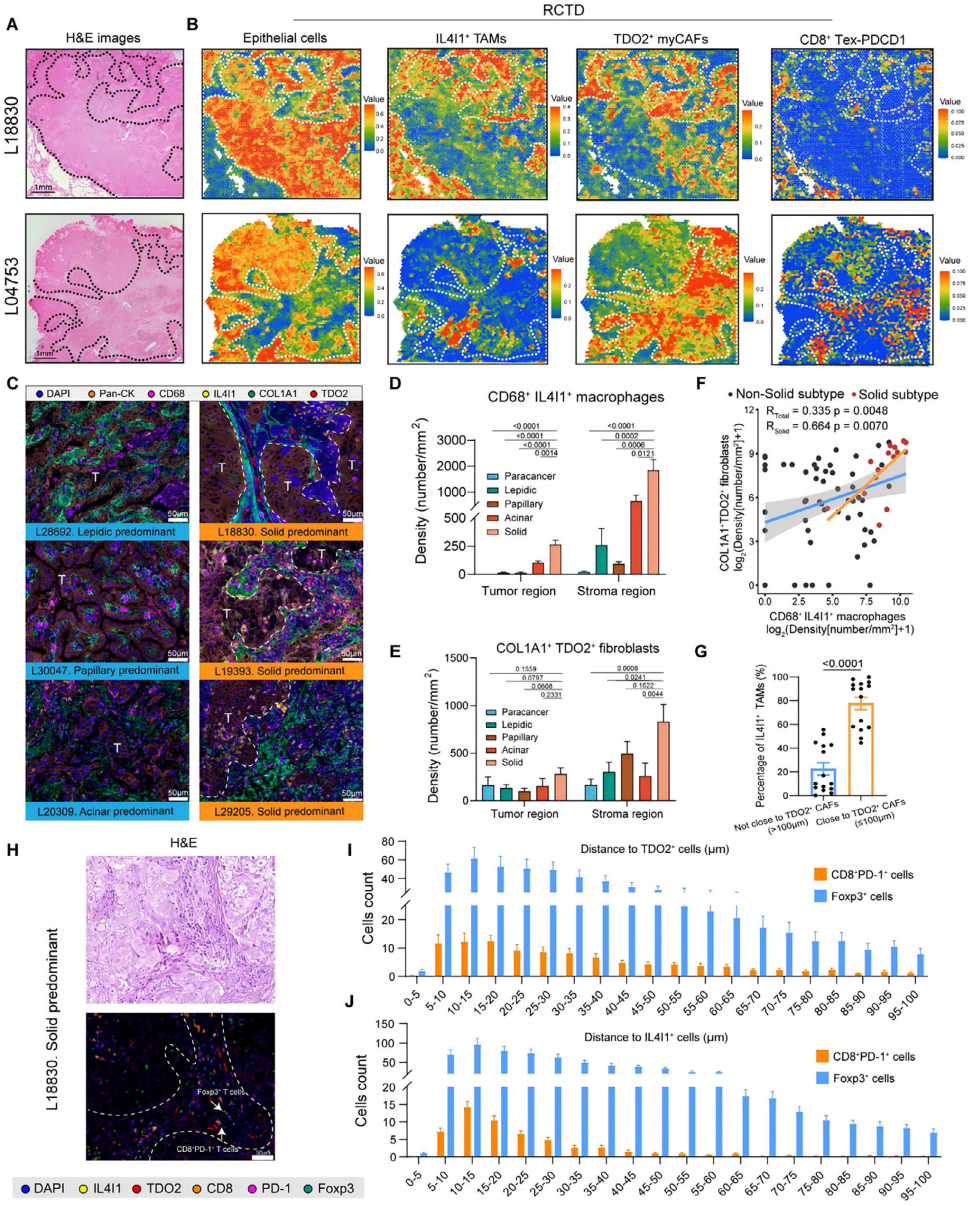
Spatial profiling reveals IL4I1^+^ TAMs and TDO2^+^ myCAFs exhibit co‐localization and recruit suppressor T cells. **A**. H&E staining of solid predominant LUAD (n = 2). Black dotted line indicating tumor‐stroma interface. **B**. RCTD algorithm showing the spatial distribution of epithelial cells, IL4I1^+^ TAMs, TDO2^+^ myCAFs, and exhausted CD8^+^ T cells. White dotted line indicating tumor‐stroma interface. **C**. Representative multiplexed IHC staining of solid predominant LUAD samples and non‐solid predominant LUAD samples stained for Pan‐CK, CD68, IL4I1, COL1A1, and TDO2. Scale bar = 50 µm. **D‐E**. Boxplots showing the density of CD68^+^ IL4I1^+^ TAMs (**D**) and COL1A1^+^ TDO2^+^ CAFs (**E**) across histological regions from LUAD patients (18 adjacent normal regions, 12 lepidic regions, 12 papillary regions, 12 acinar regions, and 15 solid regions). *P*‐values were calculated by Wilcoxon test. (**F)** Scatter plot showing the correlation between the density of CD68^+^ IL4I1^+^ TAMs and COL1A1^+^ TDO2^+^ CAFs. Red plots represent solid regions, and black plots represent non‐solid regions. P‐values and correlation coefficient were determined by the Pearson correlation test. (**G)** Bar plot showing proportions of CD68^+^ IL4I1^+^ TAMs colocalized with COL1A1^+^ TDO2^+^ CAFs in regions of solid pattern. *P*‐values were calculated with Student's *t*‐test. (**H)** Representative H&E and multiplexed IHC staining of solid predominant LUAD samples stained for IL4I1, TDO2, CD8, PD‐1, and Foxp3. White arrows indicating CD8^+^ PD‐1^+^ T cells and Foxp3^+^ T cells. Scale bar = 50 µm. (**I–J)** Bar plot showing proportions of TDO2^+^ cells and IL4I1^+^ cells in the proximity of CD8^+^ PD‐1^+^ cells and Foxp3^+^ cells in solid regions (0–100 µm).

To decipher characteristics of IL4I1^+^ TAMs and TDO2^+^ myCAFs at the spatial level, we performed 10× Genomics Visium spatial transcriptomics on tumor sections from two treat‐naïve solid predominant LUAD patients. After quality control, transcriptomics from 4,936 and 4,296 spots were obtained from L18830 (T_2a_N_0_M_0_, stage IB) and L04753 (T_1b_N_0_M_0_, stage IA). The overall organization of histological patterns and tumor‐stroma interface were annotated by two certified pathologists for hematoxylin and eosin (H&E) staining and spatial transcriptomics (Figure 3A,B). To resolve the high‐resolution spatial landscapes, we performed a robust cell type decomposition (RCTD) deconvolution algorithm to estimate the cellular composition of each spot. Refer to expression profiles of 13 major cell types in solid predominant samples from the discovery cohort including epithelial cells, IL4I1^+^ TAMs, TDO2^+^ myCAFs, CD8^+^ Tex‐PDCD1, CD4^+^ Treg‐FOXP3, monocytes, cDC1, cDC2, mast cells, B cells, plasma cells, endothelial cells, and pericytes, we found that IL4I1^+^ TAMs and TDO2^+^ myCAFs exhibited striking co‐localized features in peritumoral tissue (Figure 3B). Furthermore, spatial spots adjacent to these two groups of cells exhibited a higher percentage of CD8^+^ Tex‐PDCD1 and CD4^+^ Treg‐FOXP3 (Figure 3B; Figure S8A), indicating the formation of an immunosuppressive niche within the peritumoral microenvironment. In parallel, on the basis of typical cell type markers, we classified the spots of L18830 into four spatial niches based on unbiased clustering and histological features, including epithelial cells, IL4I1^+^ TAMs & TDO2^+^ myCAFs with adaptive immune cells, POSTN^+^ myCAFs, and SFTPC^+^ alveolar cells type 2 (AT2). Similarly, the spots of L04753 were classified into seven spatial niches, including epithelial cells, IL4I1^+^ TAMs, and TDO2^+^ myCAFs with adaptive immune cells, IL4I1^+^ TAMs, POSTN^+^ myCAFs, immune cells, and mitochondria (MT)‐cells (Figure S8B–E). Functional enrichment analysis revealed that the niche formed by IL4I1^+^ TAMs and TDO2^+^ myCAFs with adaptive immune cells is highly active in pathways including ECM organization, collagen degradation, signaling by PDGF, and immunoregulatory interactions between a lymphoid and a non‐lymphoid cell (Figure S8F). Score spots with IL4I1^+^ TAMs and TDO2^+^ myCAFs signature exhibited a significantly positive correlation in each spot (Figure S8G). To further contextualize IL4I1⁺ TAMs and TDO2⁺ myCAFs within the broader myeloid landscape, we examined the spatial distribution of M2 macrophages and myeloid‐derived suppressor cells (MDSCs). We performed the SpaCET algorithm with its pre‐annotated reference that includes malignant cells, M1‐like macrophages, M2‐like macrophages, and CAF. The results showed that both M2‐like macrophages and CAF were detectable in our sections and showed co‐localization in the peritumoral stroma and at the tumor‐stroma interface, consistent with an immune‐excluded niche (Figure S9A). The abundance of malignant cell clusters, M2 macrophages, and CAFs calculated by SpaCET closely resembled the cell populations identified in our RCTD analysis. In addition, we quantified MDSC‐related gene signatures [59, 60, 61] and visualized their spatial localization across tumor sections. However, their spatial similarity with IL4I1⁺ TAMs or TDO2⁺ myCAFs was weak to moderate (Figure S9B), suggesting that MDSC recruitment and niche formation may be governed by distinct regulatory mechanisms. Consistently, infiltration analyses using TIDE further showed that MDSC abundance exhibited weak correlations with IL4I1 or TDO2 expression (Figure S9C). Together, these results revealed that ECM‐binding components and chemokine‐driven recruitment between TDO2^+^ myCAFs and IL4I1^+^ TAMs provided a plausible explanation for their spatial co‐localization and suppressive T cell recruitment, especially within the peritumoral stromal‐enriched regions.

To validate the above findings, we localized these two cell populations on treatment‐naïve LUAD samples (*n* = 10) across histological subtypes by performing multiplexed IHC staining of Pan‐CK, CD68, IL4I1, COL1A1, and TDO2 (Figure 3C). We first performed H&E and thyroid transcription factor 1 (TTF1) staining to distinguish histological patterns and tumor cells in LUAD (Figure S10A,B). Then, we captured three representative fields from each histological region of each sample. The results found that the solid pattern had a significantly higher density of CD68^+^ IL4I1^+^ macrophages and COL1A1^+^ TDO2^+^ fibroblasts, especially in the stromal region (Figure 3D‐E). These two groups of cells exhibited a high correlation at the quantitative level (Pearson's *R* = 0.335, *p* = 0.0048), especially in the solid pattern (Pearson's *R* = 0.664, *p* = 0.0070) (Figure 3F). Besides, CD68^+^ IL4I1^+^ macrophages and COL1A1^+^ TDO2^+^ fibroblasts showed colocalization (proximity < 100 µm) in the solid pattern (*p* < 0.0001) (Figure 3G). Then, we co‐stained IL4I1 and TDO2 with T cell‐related markers, including CD8, PD‐1, and Foxp3, for serial sections of LUAD samples described above (Figure S10C). We observed that the solid region exhibited a relatively higher density of CD8^+^ PD‐1^+^ T cells and Foxp3^+^ T cells (Figure S10D). In addition, we defined IL4I1^+^ cells and TDO2^+^ cells as the spatial center and quantified the numbers of CD8^+^ PD‐1^+^ T cells and Foxp3^+^ T cells (0–100 µm) in fields of solid subtype. The results showed CD8^+^ PD‐1^+^ T cells and Foxp3^+^ T cells mostly distributed in the proximal region (< 50 µm) (Figure 3H–J). Together, these findings revealed that IL4I1^+^ TAMs and TDO2^+^ myCAFs showed a strong spatial and quantitative relevance in solid predominant LUAD and contributed to effectively decoy effector T cells within the stromal compartment, thereby hindering their ability to engage and eliminate tumor cells.

### 2.5 TDO2^+^ myCAFs Orchestrate Differentiation From Monocytes to IL4I1^+^ TAMs via Kyn‐AhR Axis

**FIGURE 4:**
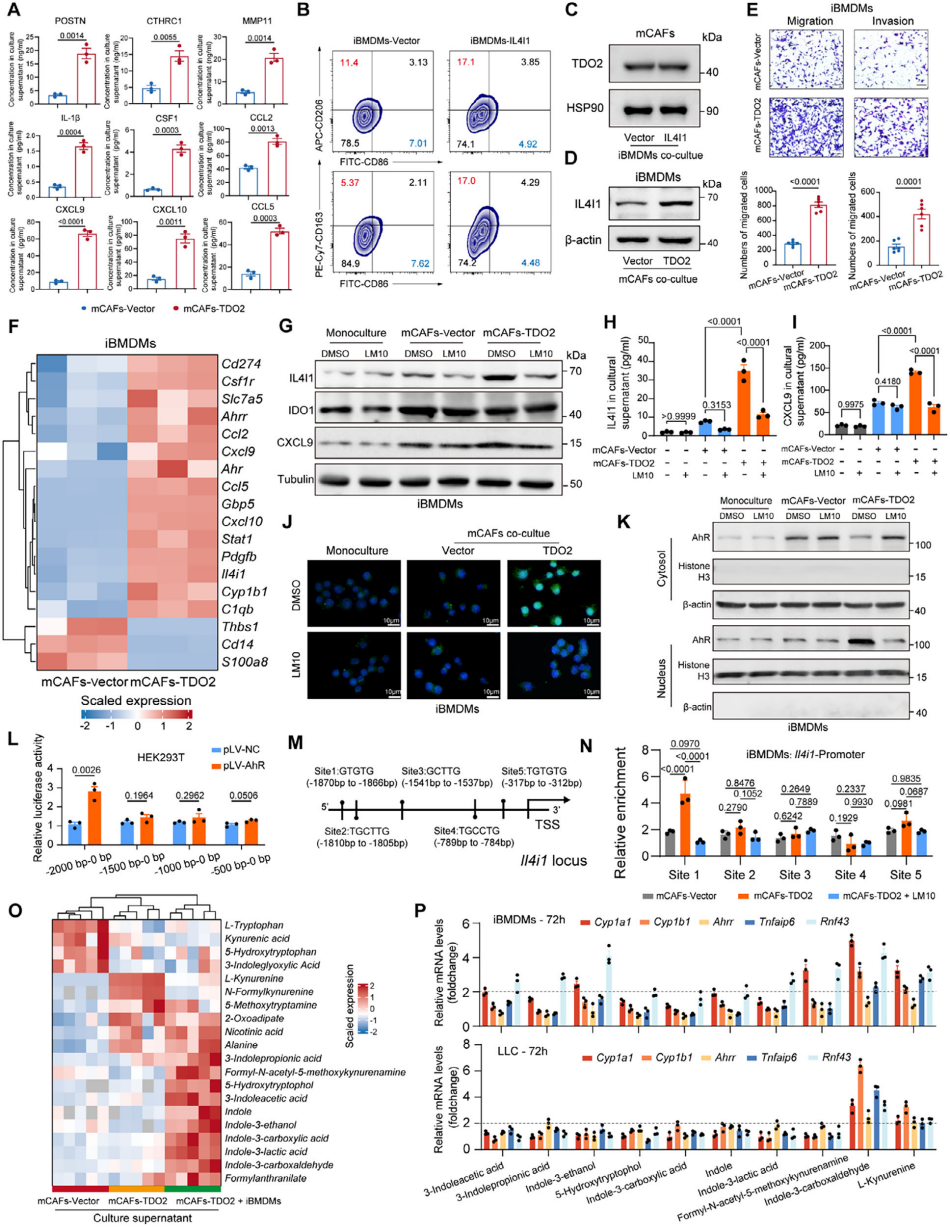
TDO2^+^ myCAFs orchestrate differentiation from monocytes to IL4I1^+^ TAMs via Kyn‐AhR axis. **(A)** Bar plot showing the concentration of POSTN, CTHRC1, MMP11, IL‐1β, CSF1, CCL2, CCL5, CXCL9, and CXCL10 in cultural supernatant of mCAFs‐Vector/TDO2 cells using ELISA (*n* = 3 per group). *P*‐values were calculated with Student's *t*‐test. (**B)** Flow cytometry analysis of M1 (CD86) and M2 (CD206 and CD163) markers in iBMDMs‐Vector/IL4I1. **(C)** Immunoblotting showing TDO2 expression in mCAFs co‐cultured with iBMDMs‐vector or iBMDMs‐IL4I1 for 72 h. (**D)** Immunoblotting showing IL4I1 expression in iBMDMs co‐cultured with mCAFs‐Vector or TDO2 cells for 72 h. (**E)** Transwell assay showing chemotaxis and infiltration of iBMDMs co‐cultured with mCAFs‐vector or mCAFs‐TDO2 (*n* = 6 per group). *P*‐values were calculated with Student's *t*‐test. (**F)** Heatmap showing the expression of IL4I1^+^ TAMs signature genes using RNA sequencing (*n* = 3 per group). (**G)** Immunoblotting showing IL4I1, IDO1, and CXCL9 expression in iBMDMs co‐cultured with mCAFs‐vector, mCAFs‐TDO2, and mCAFs‐TDO2 with TDO inhibitor LM10 for 72 h. **(H,I)** ELISA showing the concentration of IL4I1 **(H)** and CXCL9 **(I)** in culture supernatant of co‐cultured iBMDMs (*n* = 3 per group). *P*‐values were calculated with one‐way ANOVA test with Tukey's post‐hoc test. (**J)** Immunofluorescence staining showing the subcellular localization of AhR in iBMDMs co‐cultured with mCAFs for 72 h. Scale bar = 10 µm. (**K)** Immunoblotting showing AhR expression in nuclear and cytoplasmic fractions of iBMDMs monoculture or co‐cultured with mCAFs‐Vector/TDO2 for 72 h. **(L)** Bar plot showing AhR binding sites in the *Ili41* promoter region using dual luciferase reporters. Analysis of luciferase activity was performed in HEK‐293T cells transfected with pLV‐AhR or vector for 48 h (*n* = 3 per group). *P*‐values were calculated with Student's *t*‐test. (**M)** The −2000 bp to 0 bp region contained five AhR‐Arnt binding sites, as predicted by the JASPAR database. **(N)** Bar plot showing the chromatin DNA that coprecipitated with AhR protein was analyzed by qPCR using five pairs of primers designed to amplify the predicted binding sites of *Ili41* promoter region in iBMDMs co‐cultured with mCAFs for 72 h (*n* = 3 per group). *P*‐values were calculated with one‐way ANOVA test with Tukey's post‐hoc test. (**O)** Heatmap showing the abundance of Trp‐metabolites in the culture supernatant of mCAFs‐vector, mCAFs‐TDO2, and mCAFs‐TDO2 co‐cultured with iBMDMs (*n* = 5 per group). (**P)** Bar plot showing the expression of AhR downstream genes (*Cyp1a1*, *Cyp1b1*, *Ahrr*, *Tnfaip6*, *Rnf43*) using RT‐qPCR in iBMDMs and LLC cells treated with Trp‐metabolites for 72 h (*n* = 3 per group).

In view of the close spatial relationship between IL4I1^+^ TAMs and TDO2^+^ myCAFs, we hypothesized that two cell subpopulations had reciprocal regulatory mechanisms, which synergistically controlled the evolution of solid predominant LUAD. We next investigated interaction mechanisms and the downstream regulation of adaptive immune response. Given the results of gene homology analysis (Figure S11A,B) and functional conservation of TDO2 and IL4I1 between humans and mice in previous studies [62, 63], we orthotopically injected Lewis lung carcinoma (LLC) cells in immunocompetent C57BL/6 mice and isolated primary murine PDGFRα^+^ EpCAM^−^ CD45^−^ CD31^−^ CAFs (mCAFs) using fluorescence‐activated cell sorting (FACS) (Figure S12A,B). To delineate the transcriptional features of fibroblast subpopulations within the LLC‐induced TME, we analyzed the scRNA‐seq dataset GSE256051 [64]. Unsupervised clustering identified distinct cell populations, including malignant cells, myeloid cells, T/NK cells, and fibroblasts. Within the fibroblast compartment, mCAFs subset was delineated by robust expression of ECM‐remodeling related genes, including *Postn*, *Cthrc1*, *Col1a1*, *Mmp11*, and *Fap*. This subset concomitantly exhibited elevated transcripts for inflammatory cytokines and chemokines (*Cxcl9*, *Cxcl10*, *Il1b*, *Ccl2*, *Ccl5*, and *Csf1*), implicating mCAFs in immunoregulatory processes within the TME. Notably, although *Tdo2* showed relatively low expression in LLC‐induced fibroblasts overall, its expression was preferentially enriched in the mCAF subpopulation (Figure S12C). Then, we performed RT‐qPCR and western blotting to assess the expression level of TDO2 in murine normal lung fibroblasts NIH/3T3 and mCAFs, then constructed stable TDO2‐overexpressing mCAFs (mCAFs‐TDO2) (Figure S12D,E). We additionally evaluated the expression levels of representative genes in TDO2^+^ myCAFs, including POSTN, CTHRC1, MMP11, IL‐1β, CSF1, CCL2, CCL5, CXCL9, and CXCL10 using RT‐qPCR and ELISA, which exhibited increased expression at the transcriptional level and enhanced secreted concentration in culture supernatant (Figure 4A; Figure S12F). On the other hand, we overexpressed expression level of IL4I1 in immortalized bone marrow‐derived macrophages (iBMDMs‐IL4I1) (Figure S12G‐H), which induced an increase in M2‐like macrophage phenotypes (CD163 and CD206) (Figure 4B). These results validated that TDO2 and IL4I1 played functionally complementary roles in shaping the TME, with TDO2‐expressing myCAFs promoting an inflammatory and chemokine‐rich stroma and IL4I1‐expressing macrophages favoring immunosuppressive M2‐like polarization.

To clarify the regulatory relationship between mCAFs and iBMDMs, we performed the bidirectional co‐culture experiment to assay phenotypic changes. We first co‐cultured mCAFs with iBMDMs‐vector/IL4I1, but the expression of TDO2 showed no significant difference (Figure 4C). RNA sequencing showed iBMDMs‐IL4I1 promoted expression of *Ube2v1*, *Itgb2*, *Postn*, *Fap*, *Col1a1*, *Col1a2*, *Cxcl12*, *Igf1*, *Igfbp3*, *Pcdhga7*, *Pcdhgb2*, and *Pcdhgb7* in mCAFs (Figure S12I). Gene set enrichment analysis (GSEA) analysis [65] exhibited enrichment of inflammatory response pathway, ECM organization, non‐integrin membrane ECM interactions, and degradation of ECM in mCAFs co‐cultured with iBMDMs‐IL4I1, compared with the control group (Figure S12J). These findings suggested that iBMDMs‐IL4I1 promoted functional changes in mCAFs that were associated with ECM remodeling and inflammation. Subsequently, we co‐cultured iBMDMs with mCAFs‐TDO2 and discovered increased expression of IL4I1 in iBMDMs (Figure 4D). Besides, the transwell assay revealed that mCAFs‐TDO2 in the lower chamber promoted the recruitment and infiltration of iBMDMs in the upper chamber (Figure 4E). These results indicated that mCAFs‐TDO2 could recruit and support the formation of IL4I1^+^ TAMs. Then, RNA sequencing revealed that iBMDMs co‐cultured with mCAFs‐TDO2 expressed elevated marker genes like *Il4i1*, *Cxcl9*, *Cxcl10*, *Ccl2*, *Ccl5*, *Cd274*, *Slc7a5*, and *Stat1* while decreased expression of *S100A8*, *Thbs1*, and *Cd14* (Figure 4F; Figure S12K). GSEA analysis showed enrichment of human IL4I1^+^ TAMs signature and immunoregulatory interactions between a lymphoid and a non‐lymphoid cell in iBMDMs co‐cultured with mCAFs‐TDO2, compared with the control group (Figure S12L). Importantly, we utilized the Monocle2 algorithm [66] and found that these altered genes were identical within the trajectory from monocytes to IL4I1^+^ TAMs using pseudo‐time analysis (Figure S12M,N). Next, iBMDMs were cultured either monoculture or co‐cultured with mCAFs‐Vector or with mCAFs‐TDO2. Immunoblotting demonstrated that co‐culture with mCAFs‐TDO2 significantly increased IL4I1 and CXCL9 protein levels in iBMDMs relative to both monoculture and mCAFs‐Vector conditions. By contrast, IDO1 expression was elevated by the presence of mCAFs per se and was not further augmented by TDO2 overexpression, indicating that IDO1 regulation in this context was largely independent of TDO2 (Figure 4G). Consistent with these intracellular changes, measurements of culture supernatants in iBMDMs revealed higher concentrations of secreted IL4I1 and CXCL9 under the mCAFs‐TDO2 co‐culture condition (Figure 4H,I), suggesting that IL4I1^+^ TAMs may contribute to an immunosuppressive extracellular TME. Besides, flow cytometry analysis of CD86, CD206, and CD163 in iBMDMs co‐cultured with mCAFs‐TDO2 exhibited significantly higher rangeability of CD206 and CD163, relative to CD86 (Figure S12O). Notably, these effects were reversed upon treatment with the TDO inhibitor LM10. Collectively, these findings demonstrated that TDO2 in mCAFs promotes the differentiation of monocytes into IL4I1^+^ TAMs, contributing to the immunosuppressive TME.

Mechanically, previous evidence suggested that IL4I1 may be a potential target gene for AhR, which acts as a transcription factor and forms a heterodimer with ARNT to induce transcriptional activation [42]. Our RNA‐seq results also showed that iBMDMs co‐cultured with mCAFs‐TDO2 expressed AhR pathway genes, including *AhR*, *Cyp1b1*, and *Ahrr* (Figure 4F). In addition, mCAFs‐TDO2 induced a higher proportion of CD206^+^ AhR^+^ iBMDMs using flow cytometry analysis, and LM10 diminished these effects (Figure S13A). Thus, we speculated that Kyn derived from mCAFs‐TDO2 as an activating ligand for AhR in iBMDMs, thereby inducing IL4I1 expression. To test this hypothesis, we treated iBMDMs with exogenous Kyn in a dose‐gradient manner (0, 50, 100, and 200 µm). Our findings revealed a significant upregulation of IL4I1 expression following Kyn treatment, which could be abrogated upon co‐treatment with AhR antagonist CH‐223191 (Figure S13B), suggesting that Kyn promoted IL4I1 expression through activation of AhR signaling in macrophages. Furthermore, immunofluorescence staining and nucleoplasmic separation revealed that iBMDMs co‐cultured with mCAFs‐TDO2 showed significant AhR nuclear translocation, and LM10 reversed this effect (Figure 4J,K). To evaluate the regulatory elements in the *Il4i1* promoter region, we generated a series of luciferase reporter constructs containing four truncated fragments upstream of the *Il4i1* transcription start site (TSS) (−2000 to 0 bp, −1500 to 0 bp, −1000 to 0 bp, and −500 to 0 bp). After co‐transfection of pLV‐AhR and promoter region sequence in HEK‐293T cells, the −2000 to 0 bp region significantly upregulated the luciferase signal (Figure 4L), which indicated that the segment between −2000 and −1500 bp may contain AhR binding xenobiotic response elements (XREs). Then, we predicted potential transcription factor binding sites (TFBS) using JASPAR database (Figure S13C). We identified five candidate TFBS in the promoter region of the *Il4i1* gene (Figure 4M). Next, we conducted a chromatin immunoprecipitation‐quantitative polymerase chain reaction (ChIP‐qPCR) experiment in iBMDMs and confirmed binding sites of AhR to the promoter region (−1870 to −1866 bp) of *Il4i1* following mCAFs‐TDO2 treatment (Figure 4N), which was significantly reduced by LM10 treatment. Collectively, these results supported that TDO2 promoted IL4I1 transcription through Kyn‐AhR pathway.

### 2.6 TDO2⁺ myCAFs and IL4I1⁺ TAMs reprogram Trp Metabolic Landscapes in the TME

Previous research indicated that Trp metabolism primarily involved three metabolic pathways: Kyn, serotonin, and indole pathways [67]. Next, we investigated Trp metabolic landscapes in cultural supernatant of mCAFs‐vector, mCAFs‐TDO2, and mCAFs‐TDO2 co‐cultured with iBMDMs using liquid chromatography‐tandem mass spectrometry (LC‐MS/MS). We performed principal components analysis (PCA) and correlation analysis of metabolite profiles in the cultural supernatant (Figure S14A,B), indicating significant alterations in Trp metabolism associated with TDO2 overexpression or co‐culture conditions. Compared to mCAFs‐vector, both mCAFs‐TDO2 monoculture and co‐cultured with iBMDMs substantially degraded Trp in culture medium (Figure 4O; Figure S14C,D). Specifically, in mCAFs‐TDO2 monoculture, we detected increased levels of Kyn metabolic pathway products, including Kyn, N‐formylkynurenine (NFK), accompanied by a marked decrease in kynurenic acid (KynA). In contrast, the cultural supernatant of mCAFs‐TDO2 co‐cultured with iBMDMs revealed that the absence of IL4I1‐derived metabolite I3P but multiple known I3P derivatives exhibited elevated concentrations, including 3‐indoleacetic acid (IAA), 3‐indolepropionic acid (IPA), indole‐3‐ethanol (I3E), indole‐3‐carboxylic acid (I3CA), indole‐3‐lactic acid (ILA), and indole‐3‐carboxaldehyde (I3A). In addition, serotonin metabolites, including 5‐hydroxytryptophol (5‐HTOL) and formyl‐N‐acetyl‐5‐methoxykynurenamine (AFMK), also showed increased levels. Notably, although the mCAFs‐TDO2 co‐cultured with iBMDMs exhibited a markedly elevated Kyn level compared to the mCAFs‐vector group, the overall accumulation of Kyn was notably lower than that observed in mCAFs‐TDO2 monoculture (Figure S14D). This reduction may be attributed to active uptake and metabolism of Kyn by iBMDMs, possibly through AhR signaling or downstream catabolic pathways. In addition, competition for the upstream substrate tryptophan between mCAFs and iBMDMs may further limit Kyn production by restricting TDO2 enzymatic activity. Together, these results highlight a dynamic metabolic crosstalk between stromal and myeloid cells in the TME.

To functionally assess their impact on distinct cell types in the TME, we reconfigured discrepant metabolites according to LC‐MS/MS quantification and treated iBMDMs and LLC cells for 72 h. Through evaluating the expression of canonical AhR target genes, including *Cyp1a1*, *Cyp1a2*, *Ahrr*, *Tnfaip6*, and *Rnf43*, we found that I3A as I3P derivant, and Kyn exerted the higher activation capacity of AhR (Figure 4P). Overall, the diverse range of Trp metabolites produced by mCAFs‐TDO2 co‐cultured with iBMDMs suggested competition between TDO2 and IL4I1 for available Trp may drive their accumulation in the TME of solid predominant LUAD. These findings indicated that TDO2⁺ myCAFs in solid patterns not only shaped the immunosuppressive TME through Trp metabolism but also facilitated the formation of IL4I1⁺ TAMs, collectively amplifying AhR‐mediated immune suppression and promoting tumor malignancy.

### 2.7 IL4I1^+^ TAMs and TDO2^+^ myCAFs Synergistically Promote CD8^+^ T Cell Exhaustion and Immunotherapy Resistance

**FIGURE 5:**
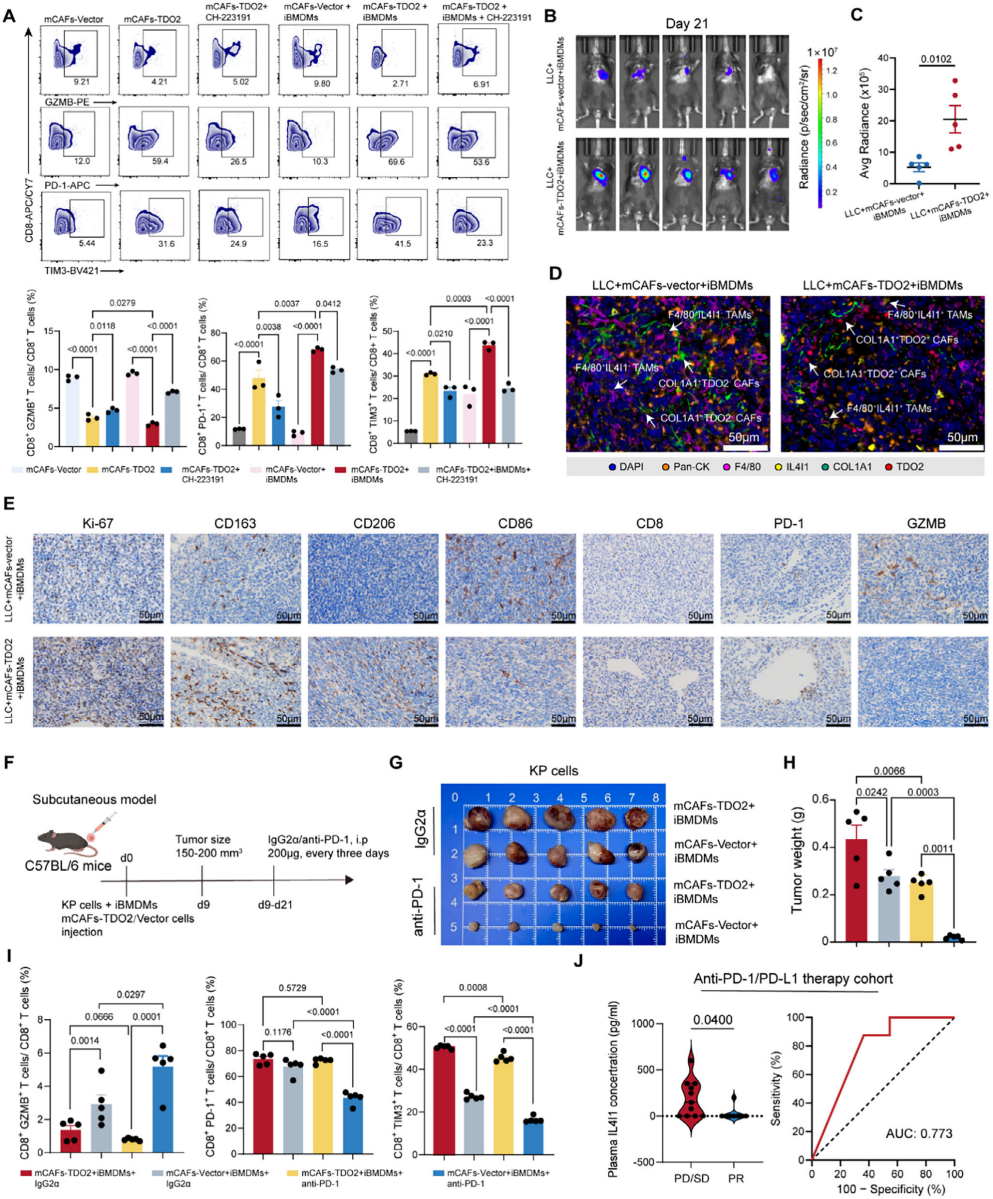
IL4I1^+^ TAMs and TDO2^+^ myCAFs synergistically promote CD8^+^ T cell exhaustion and immunotherapy resistance. (**A)** Flow cytometry analysis of CD8, GZMB, PD‐1, and TIM3 expression in murine CD8^+^ T cells co‐cultured with mCAFs and iBMDMs (*n* = 3 per group). *P*‐values were calculated with one‐way ANOVA test with Tukey's post‐hoc test. (**B)** In vivo bioluminescence imaging showing the growth of orthotopic lung tumors constructed by LLC, mCAFs‐vector/TDO2, and iBMDMs on day 21 (*n* = 5 per group). (**C)** Normalized bioluminescence imaging quantification of orthotopic lung tumors (*n* = 5 per group). *P*‐values were calculated with Student's *t*‐test. (**D)** Representative multiplexed IHC staining of orthotopic lung tumors stained for Pan‐CK, IL4I1, TDO2, F4/80, and COL1A1. Scale bar = 10 µm. (**E)** Representative IHC staining of orthotopic lung tumors stained for Ki‐67, CD163, CD206, CD86, CD8, PD‐1, and GZMB. Scale bar = 50 µm. (**F)** Schematic outline showing anti‐PD‐1 treatment for subcutaneous xenograft tumor formed by mouse KP lung cancer cells, mCAFs‐vector/TDO2, and iBMDMs in C57BL/6 mice. (**G)** Gross appearance of subcutaneous xenograft tumor (*n* = 5 per group). (**H)** Tumor weight of subcutaneous xenograft tumor formed by mouse KP lung cancer cells, mCAFs‐vector/TDO2, and iBMDMs in the treatment of IgG2α and anti‐PD‐1 (*n* = 5 per group). *P*‐values were calculated with one‐way ANOVA test with Tukey's post‐hoc test. (**I)** Bar plot showing the frequency of CD8^+^ GZMB^+^ T cells, CD8^+^ PD‐1^+^ T cells among CD8A^+^ T cell, and CD8^+^ TIM3^+^ T cells (*n* = 5 per group). *P*‐values were calculated with one‐way ANOVA test with Tukey's post‐hoc test. (**J)** The concentrations of IL4I1 in the LUAD patients accepted anti‐PD‐1/PD‐L1 treatment. Violin plot showing the concentrations of blood plasma IL4I1 in PR (*n* = 8) and SD/PD (*n* = 11) LUAD patients (left). *P*‐values were calculated with Student's *t*‐test. Receiver operating characteristic (ROC) curves showing the diagnostic accuracy of IL4I1 (right) for prediction of immunology response.

Given that AhR activation suppresses anti‐tumor immunity by up‐regulating co‐inhibitory checkpoints on CD8⁺ T cells [43], we next examined whether IL4I1^+^ TAMs and TDO2^+^ myCAFs cooperatively modulate CD8⁺ T cell function via Trp metabolism. We co‐cultured CD8^+^ T cells with mCAFs‐TDO2/vector monoculture or mCAFs‐TDO2/vector co‐cultured with iBMDMs in vitro. According to the gating strategy outlined in Figure S15A, flow cytometry analysis revealed that mCAFs‐TDO2 promoted the differentiation of CD8⁺ T cells into an exhausted phenotype, characterized by increased expression of PD‐1 and TIM‐3, along with reduced cytotoxicity levels of granzyme B (GZMB) (Figure 5A). Moreover, the co‐culture of mCAFs‐TDO2 and iBMDMs resulted in a more significant induction of CD8⁺ T cell exhaustion (Figure 5A). Besides, we observed a concomitant increase in the expression of AhR in CD8⁺ T cells. The proportions of AhR^+^ PD‐1^+^ and AhR^+^ TIM3^+^ in CD8^+^ T cells were both elevated in co‐culture conditions (Figure S15B,C). Importantly, treatment with CH‐223191 effectively reversed exhausted phenotype and restored CD8⁺ T cell cytotoxicity (Figure 5A). Consistent with these findings, among Trp‐derived metabolites, I3A and Kyn exhibited the highest capacity to induce AhR^+^ PD‐1^+^ and AhR^+^ TIM‐3^+^ in CD8^+^ T cells (Figure S15D,E). Furthermore, we directly co‐cultured triple‐cell system for 72 h, including LLC‐luciferase cells, mCAFs‐TDO2/vector, and iBMDMs, then orthotopically implanted triple‐cell model into the C57BL/6 mice (Figure S15F). In orthotopic lung tumors, in vivo imaging and bioluminescence analysis revealed that mCAFs‐TDO2 and iBMDMs substantially promoted tumor proliferation and intrathoracic metastasis (Figure 5B,C; Figure S15G). Furthermore, multiplexed IHC staining identified that COL1A1^+^ TDO2^+^ mCAFs and F4/80^+^ IL4I1^+^ TAMs significantly infiltrated in the lung tumors induced by LLC, mCAFs‐TDO2, and iBMDMs (Figure 5D). IHC staining revealed that mCAFs‐TDO2 and iBMDMs effectively led to accumulation of Ki‐67^+^ cells, CD206^+^ cells, CD163^+^ cells, CD8^+^ cells, and PD‐1^+^ cells while reduced GZMB^+^ and CD86^+^ cells (Figure 5E). These findings revealed that the interaction of mCAFs‐TDO2 and iBMDMs constructed the immunosuppressive TME in vivo.

Previous studies have indicated that IL4I1 and TDO2 confer resistance to ICIs by promoting an immunosuppressive TME. In support, bulk RNA‐seq from Braun et al.’s study (*n* = 181) revealed that high expression of IL4I1 and TDO2 predicted the shorter OS in advanced clear cell renal cell carcinoma patients accepted anti‐PD‐1 therapy (Figure S16A). Next, considering that previous research reported that LLC cells were insensitive to anti‐PD‐1 therapy [68], we employed Kras^G12D^Tp53^−/−^ (KP) lung cancer cells to investigate the response to anti‐PD‐1 treatment in vivo. We subcutaneously implanted triple‐cell system, including KP cells, mCAFs‐TDO2/vector, and iBMDMs into the C57BL/6 mice and treated with anti‐PD‐1 therapy (Figure 5F). Tumor weight and volume showed that mCAFs‐TDO2 and iBMDMs promoted anti‐PD‐1 resistance, compared to mCAFs‐vector and iBMDMs (Figure 5G,H; Figure S16B). Flow cytometry analysis showed IgG2α‐treated lesions of mCAFs‐TDO2 and iBMDMs group had higher proportions of exhausted CD8^+^ T cells (CD8^+^ PD‐1^+^ T cells and CD8^+^ TIM3^+^ T cells) and lower proportions of cytotoxic CD8^+^ T cells (CD8^+^ GZMB^+^ T cells) than mCAFs‐vector and iBMDMs group. Besides, anti‐PD‐1 therapy reduced the expression of PD‐1 and TIM3 and enhanced the production of GZMB in CD8^+^ T cells in the group of mCAFs‐vector and iBMDMs, compared to mCAFs‐TDO2 and iBMDMs group (Figure 5I).

Given that IL4I1 is a secreted protein, it suggests a potential predictive biomarker in peripheral blood samples. We retrospectively collected plasma samples from 19 advanced‐stage LUAD patients receiving anti‐PD‐L1/PD‐1 therapy to assay the expression level of IL4I1 in plasma (Figure S16C). According to RECIST guidelines, progressive disease (PD) or stable disease (SD) patients (*n* = 11) exhibited a significantly higher concentration of IL4I1, compared to the partial response (PR) patients (*n* = 8), with the area under the curve (AUC) of 0.773 (Figure 5J). Together, these findings suggested that IL4I1^+^ TAMs and TDO2^+^ myCAFs synergistically drove immune evasion and anti‐PD‐1 resistance, highlighting the need for combination strategies targeting the immunosuppressive stromal components. IL4I1 may serve as a valuable biomarker for predicting the response of patients with LUAD who require ICIs therapy.

### 2.8 AhR Activation Promotes Ferroptosis Resistance and Enables Pharmacological Reversal in LUAD

**FIGURE 6:**
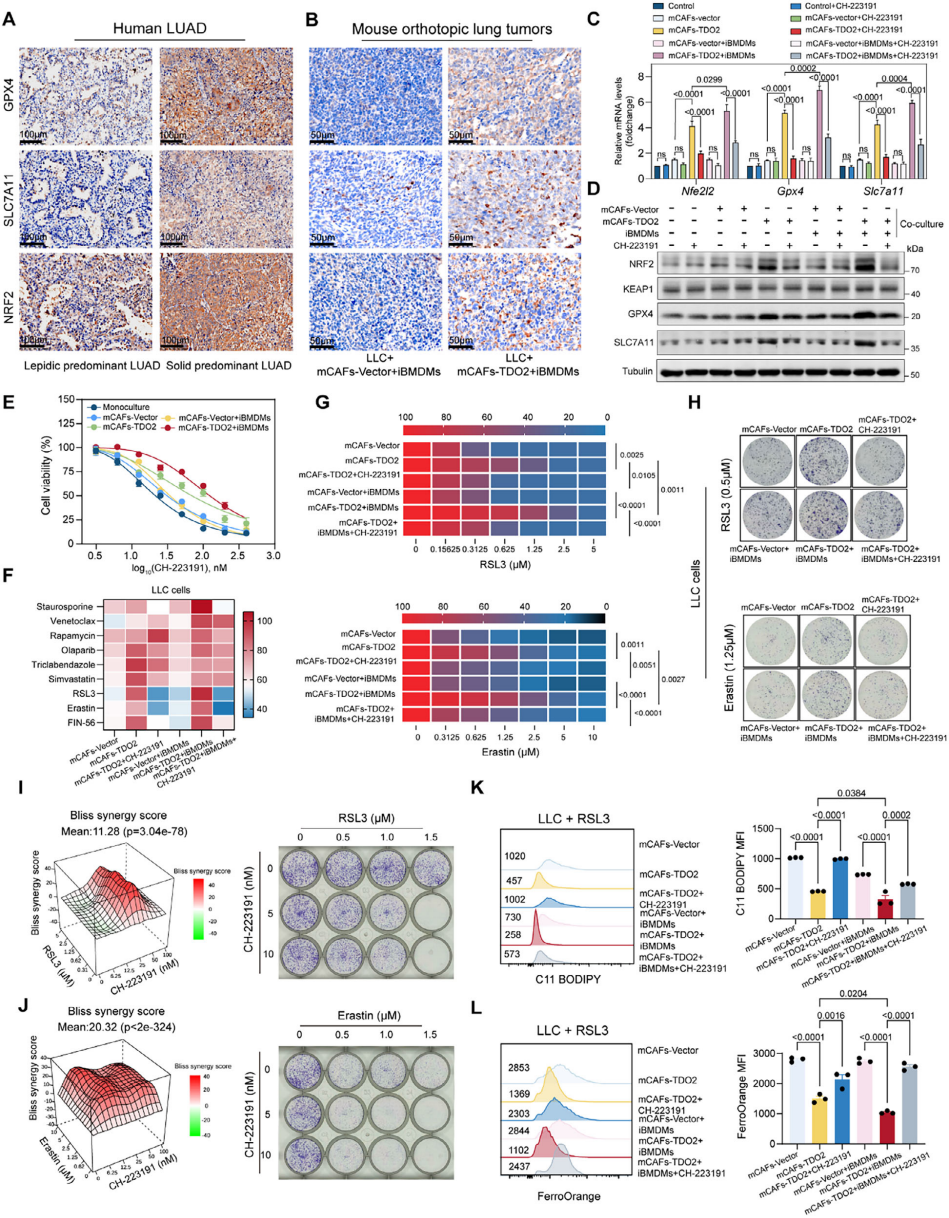
AhR activation promotes ferroptosis resistance and enables pharmacological reversal in LUAD. (A) Representative IHC staining of GPX4, SLC7A11, and NRF2 in human LUAD tissues. Scale bar = 100 µm. (B) Representative IHC staining of GPX4, SLC7A11, and NRF2 in mouse orthotopic lung tumors. Scale bar = 50 µm. (C) Bar plot showing the expression of *Nfe2l2*, *Gpx4*, and *Slc7a11* using RT‐qPCR in LLC cells monoculture or co‐cultured with mCAFs‐Vector/TDO2 ± iBMDMs and treated with DMSO or CH‐223191 for 72 h (*n* = 3 per group). *P*‐values were calculated with one‐way ANOVA test with Tukey's post‐hoc test. (D) Immunoblotting showing NRF2, KEAP1, SLC7A11, and GPX4 expression in LLC cells monoculture or co‐cultured with mCAFs‐Vector ± iBMDMs and treated with DMSO or CH‐223191 for 72 h. (E) Cell viability of LLC in monoculture or co‐culture with mCAFs‐Vector/TDO2 ± iBMDMs, following treatment with increasing concentrations of CH‐223191 (*n* = 3 per group). (F) Heatmap showing cell viability of co‐cultured LLC cells treated with programmed cell death inducers (*n* = 3 per group). (G) Heatmap showing the cell viability of co‐cultured LLC cells treated with increasing doses of ferroptosis inducers RSL3 and Erastin (*n* = 3 per group). *P*‐values were calculated with a one‐way ANOVA test with Tukey's post‐hoc test. (H) Colony formation assays showing ferroptosis resistance of LLC cells co‐cultured with mCAFs (vector or TDO2) ± iBMDMs and treated with RSL3 (0.5 µm) or Erastin (1.25 µm) and CH‐223191 treatment remodel ferroptosis sensitivity. (I,J) LLC cells were treated with increasing concentrations of RSL3 (I) or Erastin (J), alone or in combination with CH‐223191, followed by colony formation assays. Bliss synergy scores were calculated to evaluate drug interactions. 3D synergy plots showing Bliss synergy scores (left). Representative colony formation images (right). (K) Lipid ROS levels were assessed by flow cytometry using C11‐BODIPY staining (*n* = 3 per group). *P*‐values were calculated with one‐way ANOVA test with Tukey's post‐hoc test. (L) Intracellular Fe^2+^ levels were measured using FerroOrange (*n* = 3 per group). Representative histograms (left) and quantification of mean fluorescence intensity (MFI) (right) are shown. P‐values were calculated with one‐way ANOVA test with Tukey's post‐hoc test.

Despite immunotherapy having revolutionized cancer treatment, conventional oncologic therapies, including chemotherapy and small‐molecule targeted agents, remain the mainstay of clinical practice due to their direct tumor‐killing effects. However, intrinsic and TME‐driven resistance often limits their efficacy. Recent studies revealed that Trp metabolites‐mediated AhR activation sustained intrinsic malignant properties by preventing oxidative stress and ferroptosis through AhR‐NRF2‐GPX4‐SLC7A11 [69]. These findings exposed a potential therapeutic vulnerability where inhibition of AhR could sensitize tumors to ferroptosis. In support, AhR antagonists CH‐223191 have been reported to suppress tumor growth and overcome resistance to ferroptosis inducers in multiple tumor models. In our study, we first performed histopathological analyses and confirmed that both solid predominant LUAD samples and orthotopic lung tumors formed by LLC, mCAFs‐TDO2, and iBMDMs expressed higher levels of NRF2, GPX4, and SLC7A11 (Figure 6A,B). Furthermore, RT‐qPCR and immunoblotting found that increased expression levels of NRF2, GPX4, and SLC7A11 in LLC cells co‐cultured with mCAFs‐TDO2 and iBMDMs while these effects could be partially reversed by CH‐223191 treatment (Figure 6C,D). We then evaluated the anti‐tumor effect of CH‐223191 monotherapy on LLC cells under diverse culture conditions. The results demonstrated that CH‐223191 monotherapy markedly attenuated the in vitro viability of LLC cells (Figure 6E). Besides, even when mCAFs‐TDO2 and iBMDMs co‐culture induced an oxidative‐resistant phenotype, CH‐223191 treatment was still able to partially restrain tumor cell survival (Figure 6E). These observations indicated that AhR inhibition alone can impair tumor growth, and further imply that combining CH‐223191 with ferroptosis inducers may elicit a synergistic anti‐tumor effect by counteracting TME‐mediated resistance.

To further verify therapeutic advantages of ferroptosis over other forms of programmed cell death (PCD), we performed multiple PCD inducer challenges on LLC cells co‐cultured with mCAFs‐TDO2 and iBMDMs using novel molecules or FDA‐approved clinical drugs, including apoptosis (Staurosporine and Venetoclax), autophagy (Rapamycin and Olaparib), pyroptosis (Triclabendazole and Simvastatin), and ferroptosis (Erastin, RSL3, and FIN‐56). We found that mCAFs‐TDO2 monoculture or co‐cultured with iBMDMs induced tumor cells broad resistance to these drugs, while SLC7A11 inhibitor Erastin and GPX4 inhibitor RSL3 could be significantly alleviated by CH‐223191 (Figure 6F). CoQ10 inhibitor FIN‐56 (also inhibits GPX4) exhibited a slight effect in LLC cells co‐cultured with mCAFs‐TDO2 and iBMDMs. Then, both IC50 and colony formation assays demonstrated that CH‐223191 effectively sensitized tumor cells to RSL3/Erastin by significantly attenuating resistance conferred by mCAFs‐TDO2 monoculture or co‐cultured with iBMDMs (Figure 6G,H). Bliss synergy analysis revealed a strong cooperative effect between CH‐223191 and RSL3/Erastin (Bliss score > 10), with combination index (CI) values < 0.5 across gradient concentrations (*p* < 0.05) (Figure 6I,J). Then, assessed by BODIPY‐C11 and intracellular Fe^2+^ staining, LLC cells co‐cultured with mCAFs‐TDO2 and iBMDMs had lower lipid ROS levels (Figure 6K) and lower cellular Fe^2+^ (Figure 6L) in RSL3‐treatment, then combined with CH‐223191 treatment reversed these effects of ferroptosis resistance. These findings positioned the AhR as a regulator of system Xc^–^/GSH/GPX4 defense in solid predominant LUAD, offering a dual‐targeting strategy combining AhR antagonist with ferroptosis inducers to overcome TME‐driven therapy resistance.

### 2.9 Synergistic Efficacy of AhR Antagonist, Ferroptosis Inducer, and Immunotherapy in LUAD Treatment

**FIGURE 7:**
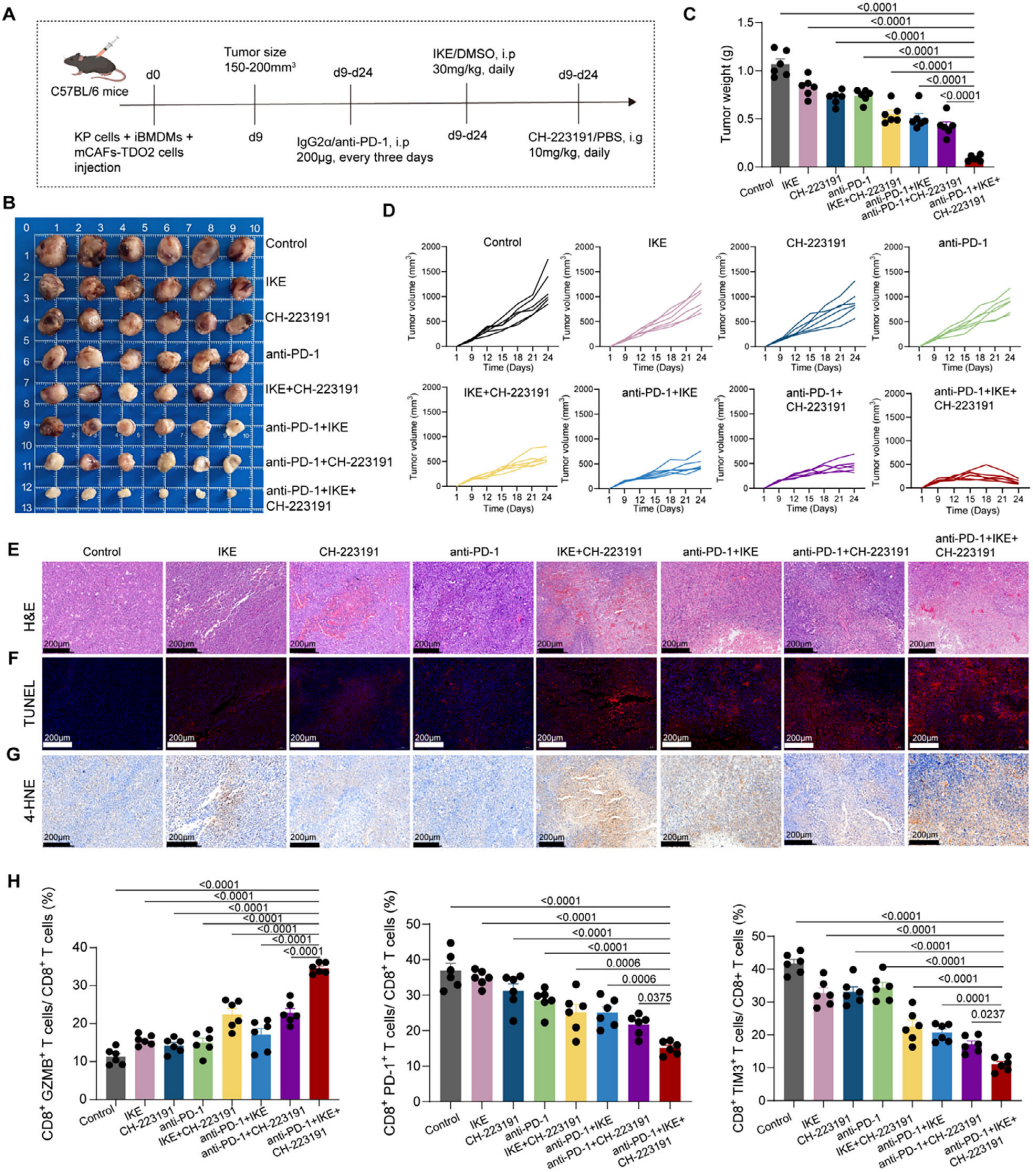
Synergistic efficacy of AhR antagonist, ferroptosis inducer, and immunotherapy in LUAD treatment. **(A)** Schematic outline showing combination treatment regimen (CH‐223191, IKE, and anti‐PD‐1) for subcutaneous tumors constructed by mouse KP lung cancer cells, mCAFs‐TDO2, and iBMDMs. (**B–D)** Gross appearance of the tumor mass (**B**), tumor weight (**C**), and kinetics of the tumor volume (mm^3^) (**D**) were measured and documented for C57BL/6 mice in group (*n* = 6 per group). (**E–G)** Representing H&E staining (**E**), TUNEL staining (**F**), and 4‐HNE staining (**G**) of tumor tissues under different treatment regimens. Scar bar = 200 µm. (**H)** Bar plot showing percentages of CD8^+^ GZMB^+^ T cells, CD8^+^ PD‐1^+^ T cells, and CD8^+^ TIM3^+^ T cells under different treatment regimens (*n* = 6 per group). *P*‐values were calculated with one‐way ANOVA test with Tukey's post‐hoc test.

On the basis of the above research evidence, we demonstrated that the spatial niche composed of IL4I1^+^ TAMs and TDO2^+^ myCAFs not only restricted CD8^+^ T cells cytotoxicity but also induced tumor cell ferroptosis resistance. Notably, AhR as a targetable protein may significantly reverse the aforementioned oncogenic effects. Therefore, we test whether CH‐223191, ferroptosis inducer (imidazole ketone erastin [IKE] or RSL3), and anti‐PD‐1 therapy exert a synergistic anti‐tumor effect in vivo. We established xenograft tumors by injecting the triple‐cell co‐culture system, including KP cells, mCAFs‐TDO2, and iBMDMs, then treated the mice with CH‐223191, IKE or RSL3, and anti‐PD‐1 therapy (Figure 7A; Figure S17A). As shown in Figure 7B,D and Figure S17B–D, combined treatment of CH‐223191, IKE or RSL3, and anti‐PD‐1 significantly inhibited tumor growth compared with monotherapy or two‐drug combination therapy. H&E staining revealed three‐drug combination group showed extensive necrotic areas, fewer residual tumors, and marked infiltration of lymphocytes and plasma cells (Figure 7E; Figure S17E). Additionally, TUNEL staining revealed the highest proportion of apoptotic cells in the three‐drug combination group, further supporting enhanced tumor cell death induced by the combination treatment (Figure 7F; Figure S17F). Notably, 4‐hydroxynonenal (4‐HNE) staining revealed a marked accumulation of lipid peroxidation products in the tumor tissues from the triple therapy group. Compared to monotherapies, the triple combination led to widespread 4‐HNE‐positive staining throughout the tumor mass (Figure 7G; Figure S17G). Furthermore, the results of flow cytometry indicated that the combined therapy induced an immune response with increased proportions of CD8^+^ GZMB^+^ T cells and decreased proportions of CD8^+^ PD‐1^+^ cells and CD8^+^ TIM3^+^ cells (Figure 7H; Figure S17H,I). We noticed that circular structures emerged exclusively in tumors treated with the combination regimen and were not detected in controls (Figure S18A). Histological analysis excluded preparation artifacts and vascular structures (marker by CD31) (Figure S18B). H&E staining revealed a sharply defined three‐layer organization: a hyalinized, acellular fibrotic core (marker by αSMA), a transitional zone with nuclear condensation and fragmentation, and an outer rim containing foam‐like stromal cells, inflammatory infiltrates, and focal fibrosis (Figure S18C,D). Consistent with these morphological features, TUNEL and 4‐HNE staining indicated localized apoptosis and lipid peroxidation (Figure S18E,F). Together, these findings suggested that the triple‐combination therapy not only enhanced ferroptosis‐mediated tumor cell death but also effectively restored CD8^+^ T cells cytotoxicity by reversing T cell exhaustion within the immunosuppressive TME.

To determine the safety of three‐drug combination, we found that no significant difference in body weight between the different treatment groups (Figure S19A,B). Besides, the immune‐related adverse events (IrAEs) of multidrug combinations on immunotherapy mainly included hepatotoxicity and cardiotoxicity. We collected peripheral blood samples of treated mice and evaluated aspartate aminotransferase (AST), alanine aminotransferase (ALT), and L‐lactate dehydrogenase (L‐LDH). The results showed that acceptable adverse events in the treated mice (Figure S19C,D). Collectively, our results provide preclinical evidence that the combination of AhR inhibitor, ferroptosis inducer with ICIs as an optimal therapy for tumors resembling solid predominant LUAD patients in terms of biological characteristics.

## 3 Discussion

Histopathological heterogeneity is a hallmark of LUAD, which reflects the trajectory of tumor evolution, clinical behavior, and stratified risk. However, whether and how molecular features determine growing patterns and histological heterogeneity is largely unexplored. In the study, we demonstrated that peritumoral IL4I1^+^ TAMs and TDO2^+^ myCAFs induced CD8^+^ cells exhaustion and tumor cells ferroptosis resistance via Trp‐metabolites activating AhR‐dependent manner in solid predominant LUAD. This conclusion is documented by the following findings: First, integrated bioinformatics analyses of bulk RNA‐seq and scRNA‐seq datasets identified IL4I1^+^ TAMs and TDO2^+^ myCAFs as pivotal cellular subpopulations specifically enriched in solid predominant LUAD. Second, spatial profiles including TMA with digital pathology, spatial transcriptomics, and multiplexed IHC emphasized IL4I1^+^ TAMs and TDO2^+^ myCAFs showed co‐localized distribution in the peritumoral region of solid growth pattern. These cells contributed to an immune‐excluded architecture, thereby preventing effective T cell infiltration into tumor parenchyma. Third, overexpression of TDO2 in CAFs promoted differentiation trajectory from monocytes to IL4I1^+^ TAMs through Kyn‐AhR‐IL4I1 axis, which further reprogrammed Trp metabolic profile involved in AhR activation, particularly elevating the levels of Trp‐metabolites such as Kyn and I3A. Fourth, the accumulation of IL4I1^+^ TAMs and TDO2^+^ myCAFs promoted AhR activation and upregulation of inhibitory checkpoints, including PD‐1 and TIM3 on CD8^+^ T cells, promoting immune exhaustion and resistance to anti‐PD‐1 therapy. Fifth, drug screening demonstrated that these cell populations conferred resistance to various PCD inducers. AhR antagonist CH‐223191 restored sensitivity to ferroptosis inducers such as GPX4 inhibitor RSL3 and SLC7A11 inhibitor Erastin. Lastly, combination therapy with CH‐223191, IKE/RSL3, and anti‐PD‐1 exerted a synergistic anti‐tumor effect in LUAD model, underscoring the therapeutic potential of targeting the IL4I1/TDO2‐AhR axis.

In line with our study, previous studies have shown that solid pattern exhibits a distinctly immune‐excluded TME characterized by low infiltration of effector T cells within the tumor core, despite the presence of abundant immune cells in the surrounding stroma. Tavernari et al. [70] revealed a stepwise evolution of the TME from immune desert (lepidic) to immune‐infiltrated (papillary/acinar) and finally to immune‐excluded (solid). Tumor cores in the solid pattern expressed increased proliferation (Ki‐67) and epithelial marker expression (EpCAM and Pan‐CK), while peripheries showed upregulation of TIM3 and VISTA. Compared with lepidic and papillary patterns, the solid pattern showed significantly reduced spatial interactions between T cells and tumor cells, emphasizing that spatial proximity, rather than T cell abundance, was more predictive of favorable outcomes. In high‐grade lung tumors, CD163^+^ M2‐like macrophages exhibited stronger interactions with CD8^+^ T cells, Foxp3^+^ Tregs, and B cells, supporting their immunosuppressive role within the TME [15]. Besides, a scRNA‐seq study has identified subpopulations of CAFs associated with T cell exclusion in NSCLC, including MYH11^+^ ACTA2^+^ CAFs observed in early‐stage tumors and FAP^+^ ACTA2^+^ CAFs observed in advanced‐stage tumors. MYH11^+^ACTA2^+^ CAFs were mainly enriched in LUAD samples, particularly within acinar and papillary subtypes, whereas they were largely absent in the solid subtype of LUAD and in LUSC samples [71]. Our findings supported and extended previous findings regarding the immune‐excluded TME in solid pattern. Specifically, we identified that IL4I1^+^ TAMs and TDO2^+^ myCAFs could recruit and decoy CXC3R^+^ T cells through chemokines CXCL9 and CXCL10, which showed a co‐localization with suppressor T cells using spatial profiles. These suggest that aberrant chemokine signaling may contribute to the establishment of an immune‐excluded niche, providing a mechanistic basis for spatial disconnection between immune cells and tumor cells in solid pattern.

Furthermore, the simple recruitment of CD8^+^ T cells by the tumor stroma is insufficient to prevent cancer cells from anti‐tumor immunity^74^. IDO1/2, TDO2, and IL4I1 participate in the process of Trp degradation, which mediates immunosuppression by AhR activation [62]. Recent studies have demonstrated that TDO2‐expressing fibroblasts and IL4I1‐expressing macrophages hinder effective anti‐tumor immune responses across multiple tumor types. Hu et al. [72] demonstrated that TDO2^+^ myofibroblasts in oral squamous cell carcinoma (OSCC) attracted both CD4^+^ and CD8^+^ T cells through the CXCL9/CXCL10/CXCL11‐CXCR3 axis, which formed a barrier to prevent T cells from attacking tumors and caused T cell inhibition through Kyn produced by TDO2. Besides, TDO2^+^ matrix fibroblasts promoted pulmonary metastasis of breast cancer, recruited T cells through CCL8 and CCL11, and induced T cell dysfunction by producing Kyn [73]. On the other hand, IL4I1 is another Trp‐metabolizing enzyme predominantly upregulated in TAMs, monocyte‐derived DCs, macrophages residing in granulomas, microglia, and B cells [62]. The single‐cell landscape of human monocytes and macrophages has revealed that IL4I1^+^ PD‐L1^+^ IDO1^+^ and TREM2^+^ TAM subsets accumulated in tumor tissue, and the former was detected in greater proportions in the tumor periphery exhibiting immunosuppressive property [79]. IL4I1 also reprogrammed an immunosuppressive TME in melanoma and thereby conferred resistance to anti‐PD‐L1 therapy [75]. Additionally, TAMs in lung cancer patients generally expressed M2‐like signature (*MMP12*, *WNT5A*, *IL10*, *ADORA3*, *IL4I1*, and *CD209*) and 25% of patients co‐expressed a strong signature of M1‐like genes (*CXCL9*, *CXCL10*, *CXCL11*, *CXCL12*, *STAT1*, and *FAM26F*), which associated with CD8^+^ tissue‐resident memory T cells in lung tumors [76]. Notably, Li et al. [77] reported that thymol inhibited IL4I1 expression and blocked AhR signaling, thereby sensitizing immunotherapy in LUAD.

In our findings, we observed a strong correlation and spatial co‐localization between TDO2^+^ myCAFs and IL4I1^+^ TAMs, suggesting a potential intercellular crosstalk. Building on this hypothesis, we demonstrated that TDO2^+^ myCAFs facilitated macrophage recruitment and stromal infiltration, and further promoted the phenotypic transition toward IL4I1^+^ TAMs through AhR transcriptional activation. Mechanistically, macrophages display various phenotypes and adapt to environmental signals by undergoing metabolic remodeling. Chen et al. [78] demonstrated that concurrent inhibition of Trp catabolism by IDO1 and IL4I1 inhibitors suppressed the macrophage pro‐inflammatory response, whereas single inhibition promoted pro‐inflammatory activation. Interestingly, our study revealed that significant elevation of IL4I1 and pro‐inflammatory factor CXCL9 induced by mCAF‐TDO2 appeared to provide evidence for the shift between different inflammatory states. Especially, integrating spatial‐level findings, CXCL9‐mediated pro‐inflammatory regulation in the context of enriched tumor stromal components seemed to favor a mechanism that restricted direct T cell‐tumor interactions and suppressed T cell cytotoxicity, thereby promoting an immune‐suppressive inflammatory TME. In support, among identified and quantified Trp metabolites in the cultural supernatant of TDO2^+^ myCAFs and IL4I1^+^ TAMs, Kyn and I3A exhibited the strongest agonistic activity toward AhR, which may explain upregulation of inhibitory immune checkpoints and downregulation of cytotoxic molecules in CD8^+^ T cells. Although IL4I1^+^ TAMs in lung cancer exhibited immunosuppressive phenotypes and led to poor clinical outcomes, Matusiak et al. []^92^ reported that IL4I1^+^ macrophages in colorectal cancer primarily function as phagocytes of dying cells in regions of high turnover and were associated with a favorable prognosis. These contradictory observations suggested that the function of IL4I1 may be influenced by tumor type and underlying genetic background. More studies are warranted to delineate the molecular determinants of IL4I1^+^ TAM heterogeneity and their distinct roles in shaping the TME across cancers. Further, subcutaneous xenograft tumor comprised of TDO2^+^ myCAFs and IL4I1^+^ TAMs demonstrated resistance to anti‐PD‐1 therapy. Flow cytometry analysis of tumor tissues after treatment suggested that persistent and irreversible immune exhaustion was a potential cause of resistance. Moreover, the observation that IL4I1 levels were elevated in the peripheral blood of advanced‐stage LUAD patients with PD/SD based on RECIST guidelines suggested a potential for predictive biomarker development. Likewise, inhibition of TDO2 and anti‐PD‐1 therapy prevented tumor progression in OSCC models [72]. Besides, a recent study on relapsed diffuse large B‐cell lymphoma revealed that knock‐out IL4I1 expression in macrophages improved poor efficacy of CD19 chimeric antigen receptor (CAR) T‐cell therapy combined with PD‐1 inhibitor in relapsed/refractory diffuse large B‐cell lymphoma [80]. Circulating IL4I1 levels may serve as a valuable biomarker for early identification of patients at risk for ICIs resistance, paving the way for precision‐guided combination therapies that target both metabolic rewiring and immune dysfunction.

Beyond their immunosuppressive roles, TDO2^+^ myCAFs and IL4I1^+^ TAMs were found to confer tumor cell resistance to multiple forms of PCD inducers, including apoptosis, autophagy, pyroptosis, necroptosis, and ferroptosis. This resistance phenotype likely stemmed from the metabolic reprogramming induced by Trp‐derived AhR ligands, which modulated cell survival within the TME. Remarkably, pharmacological inhibition of AhR using CH‐223191 selectively restored sensitivity to ferroptosis inducers, such as RSL3 (GPX4 inhibitor) and Erastin (SLC7A11 inhibitor), in LUAD models enriched with TDO2^+^ myCAFs and IL4I1^+^ TAMs. Similar to our findings, recent mechanistic studies have elucidated that Trp metabolites exert anti‐ferroptotic effects through eliminated lipid peroxidation and antioxidant stress pathways. Specifically, its metabolic products, particularly Kyn [81], I3P [82], serotonin [83], and 3‐hydroxyanthranilic acid [84], triggered a cell‐protective program involving key ferroptosis regulators such as GPX4, SLC7A11, HO‐1, FSP1, and FTH1. Moreover, these protective effects appeared to be mediated by NRF2 and AhR signaling, indicating the convergence of metabolic and transcriptional reprogramming in ferroptosis resistance. Our results showed that tumor cells co‐cultured with TDO2^+^ myCAFs and IL4I1^+^ TAMs exhibited sustained activation of AhR/NRF2/GPX4/SLC7A11 pathway, which uncovered a potential therapeutic target for combined treatment with AhR inhibitor and ferroptosis inducer.

Strikingly, our study proposed the combination of AhR inhibition, ferroptosis inducer, and immunotherapy as an innovative treatment strategy in LUAD. Notably, prior studies have suggested that combining ferroptosis inducers with immunotherapy can overcome resistance and enhance therapeutic efficacy [85]. Similarly, AhR antagonism has been explored as a strategy to rejuvenate exhausted T cells and reverse immunosuppression in the TME [86]. Building on these insights, our study provides a unique perspective by identifying AhR as a central node that not only suppresses immune activation but also promotes tumor cell resistance to oxidative stress‐induced ferroptosis. Based on this dual function, we rationally designed a triple‐drug combination strategy comprising AhR antagonism, ferroptosis inducer, and immune checkpoint blockade. This approach simultaneously targets distinct facets of the TME: it sensitizes tumor cells to ferroptosis by disrupting redox homeostasis, while reinvigorating anti‐tumor immunity by reversing immune cell dysfunction. By antagonizing AhR activity in both tumor and immune compartments, the strategy exploits the multifaceted role of AhR to amplify therapeutic outcomes. Importantly, preliminary data indicate that this combination can achieve these effects without inducing systemic toxicity, suggesting a favorable safety profile. Given the high recurrence rate and limited treatment options for solid predominant LUAD, this triple combination therapy represents a promising and innovative treatment strategy. Future clinical studies are warranted to validate its efficacy and safety in patients, and to identify predictive biomarkers that could guide patient selection and optimize therapeutic response.

Despite unveiling the cooperative roles of TDO2^+^ myCAFs and IL4I1^+^ TAMs in promoting immunosuppression and ferroptosis resistance via Trp metabolism and proposing a combination therapeutic strategy centered on AhR inhibition, this study has several limitations: First, although in vitro co‐culture and in vivo models support the role of the IL4I1‐TDO2‐AhR axis in CD8⁺ T cell exhaustion and ferroptosis resistance, the dynamic regulatory mechanisms within the broader tumor immune microenvironment remain to be fully clarified, particularly through conditional knockout or lineage‐tracing models. Second, the study primarily focuses on LUAD using clinical specimens, multi‐omics datasets, and murine models. The generalizability of these findings to other lung cancer subtypes or solid tumors requires further investigation. Third, current AhR antagonists and ferroptosis inducers are largely in preclinical or early‐phase development. Their clinical efficacy, safety profiles, and therapeutic windows need to be assessed in well‐designed clinical trials. Lastly, although IL4I1 and TDO2 show spatial specificity in spatial and multiplex IHC analyses, the extent of their intratumoral heterogeneity and precise association with immunotherapy response requires validation in larger, multicenter cohorts.

## 4 Conclusion

In summary, our study uncovers a critical stromal‐immune circuit in solid predominant LUAD, where peritumoral IL4I1^+^ TAMs and TDO2^+^ myCAFs cooperatively drive CD8^+^ T cell exhaustion and ferroptosis resistance via Trp metabolites‐mediated AhR activation. Through multi‐omics integration, spatial profiling, and functional validation, we demonstrate that this IL4I1/TDO2‐AhR axis underlies immune exclusion and therapeutic resistance. Targeting this pathway with AhR antagonists, ferroptosis inducer, and anti‐PD‐1 therapy achieves synergistic anti‐tumor effects, providing a rationale for combinatorial strategies in immune‐excluded LUAD.

## 5 Experimental Section

Complete materials and methods are provided in the Supplementary Information.

### 5.1 Public Datasets Collection

The bulk RNA‐seq dataset for LUAD from The Cancer Genome Atlas (TCGA) cohorts was obtained from UCSC Xena (http://xena.ucsc.edu/), and corresponding histopathological information was obtained from the previous study. The RNA‐seq dataset of LUAD for the East Asian ancestry cohort was obtained from Chen et al.’s study [56]. The RNA‐seq dataset of early‐stage LUAD with corresponding histopathological information was derived from the Gene Expression Omnibus (GEO) with accession numbers GSE166720 [52]. The RNA‐seq dataset of advanced clear cell renal cell carcinoma treated with anti‐PD‐1 therapy was derived from Braun et al.’s study [87]. Normalization of the count data was computed based on the R package “DESeq2” (v1.38.2) [88] transformation, and the public normalized gene expression data based on fragments per kilobase of exon model per million reads mapped (FPKM) was converted into Transcript Per Million (TPM), which were used as the gene expression matrix for downstream analysis. Moreover, multiple microarray datasets of lung cancer with clinical data (e.g., histological subtype or survival time) used in this study were retrieved from GSE58772 [24], GSE14814 [51], GSE31210 [55], GSE30219 [57], and GSE68465 [53]. This data normalization process was conducted using the R package “limma” (v3.54.1) [89]. The scRNA‐seq dataset of human LUAD with histopathological information was retrieved from Bischoff et al.’s study [37], PRJNA634159 [44], CRA001963 [45], GSE200972 [46], GSE189357 [47], and GSE131907 [48]. The scRNA‐seq dataset of murine lung tumors was retrieved from GSE256051 [64]. The scRNA‐seq dataset of human lung cancer was retrieved from Lambrechts et al.’s study [58] and the processed count data were available from the tumor immune single cell hub 2 (TISCH2) database [90]. The level 4 RPPA dataset of LUAD patients, including approximately 300 protein markers, covering all major cancer signaling pathways was retrieved from the cancer proteome atlas (TCPA) [54].

### 5.2 10x Visium Spatial Transcriptomics Sequencing and Processing

We collected two formalin‐fixed paraffin‐embedded (FFPE) sections of pathologically diagnosed early‐stage solid predominant LUAD from the First Affiliated Hospital of Dalian Medical University to perform spatial transcriptomics sequencing. The clinical datasets were summarized in Table S2. The capture of gene expression information was performed by the Visium Spatial platform of 10x Genomics through the use of spatially barcoded mRNA‐binding oligonucleotides in the default protocol. Raw sequencing reads were quality checked and mapped by Space Ranger on the reference GRCh38 human reference genome to estimate gene expression on spots. The gene‐spot matrices generated after spatial transcriptomics processing were analyzed with the R package Seurat (v4.3.0). We processed the spatial transcriptomics using Seurat, beginning with the Load10X_Spatial function. To ensure data quality, we filtered the spots, retaining only those with a minimum of 200 detected genes. Genes with fewer than 10 read counts or expressed in fewer than 3 spots were excluded. Then, we applied the SCTransform function to normalize the spots, followed by independent component analysis for dimensionality reduction and spot clustering. Robust cell type decomposition (RCTD) [91] algorithm was used to assign cell types from Bischoff et al.’s study. Cluster markers were identified with Wilcoxon tests as implemented in the function “FindAllMarkers”. Cell signature scoring was conducted using function “AddModuleScore” with default settings. Spatial feature expression plots were created using the function “SpatialFeaturePlot”.

### 5.3 Cell Lines and Cell Culture

Murine lung cancer Lewis lung carcinoma (LLC), murine lung fibroblasts NIH/3T3 cells, and human embryonic kidney (HEK)‐293T cells were purchased from the American Type Culture Collection (ATCC). Immortalized bone marrow‐derived macrophages (iBMDMs) and murine lung cancer Kras^G12D^Tp53^−/−^ (KP) cells were kindly provided by Prof. F. Yao (Shanghai Jiao Tong University). These cells were cultured in Dulbecco's modified Eagle's medium (DMEM) supplemented with 10% fetal bovine serum (FBS) and 1% penicillin‐streptomycin. All cells were maintained in a humidified incubator with 5% CO_2_ at 37°C.

### 5.4 Isolation of Primary Cells

Primary murine CAFs were isolated from lung tumors following orthotopic injection of LLC cells. Briefly, LLC cells were injected into the left lung of C57BL/6 mice. After 28 days, tumor‐bearing mice were sacrificed, and tumors were carefully excised. Briefly, sheared tissues (approximately 1 mm^3^) were digested with collagenase type I and collagenase type II in DMEM with 10% FBS and penicillin‐streptomycin. Following filtration and red blood cell lysis, single‐cell suspensions were subjected to sort by fluorescence‐activated cell sorting (FACS) using antibodies including APC anti‐PDGFRα (135907, BioLegend, 1:200), FITC anti‐EpCAM (118207, BioLegend, 1:200), PerCP/Cyanine5.5 anti‐CD45 (103131, BioLegend, 1:200), and PE anti‐CD31 (102407, BioLegend, 1:200). The PDGFRα^+^ EpCAM^−^ CD45^−^ CD31^−^ mCAFs were sorted and cultured in DMEM medium for downstream analyses. All procedures used mCAFs up to the 10th passage.

Primary murine CD8^+^ T cells were isolated from the spleen of 6‐ to 8‐week‐old male C57BL/6 mice using the MojoSort Mouse CD8 T Cell Isolation Kit (480008, BioLegend) following the manufacturer's instructions. Mouse T‐Activator CD3/CD28 for T cell expansion and activation for 48 h (anti‐mouse CD3, 5 µg/mL, 100238, BioLegend; anti‐mouse CD28, 1 µg/mL, 102116, BioLegend). Murine CD8^+^ T cells cultured in RPMI 1640 supplemented with 10% FBS, 1% penicillin‐streptomycin, and mouse IL‐2 (200 ng/mL, 212‐12‐20UG, PeproTech).

### 5.5 RNA Isolation and RT‐qPCR

Total RNA was extracted with TRIzol reagent (Invitrogen) and reverse transcribed into complementary DNA with a reverse transcription kit (R223‐01, Vazyme). RT‐qPCR was performed using the ChamQ Universal SYBR qPCR master mix (Q711‐02, Vazyme) according to the manufacturer's protocol based on the Mx3000p Instrument (Aglient). The data were analyzed by the 2^−ΔΔCt^ method. The primers used for amplification are listed in Table S3.

### 5.6 Flow Cytometric Analysis

The cells in each co‐culture system and single‐cell suspension were harvested. Cells were incubated with LIVE/DEAD Fixable Aqua Dead Cell Stain Kit (L34966, Invitrogen, 1:200) or Fixable Viability Dye eFluor (65‐0866‐18, eBioscience, 1:200) and Fc receptor blocking reagent (553142, BD Pharmingen, 1:400) on ice for 20 min and centrifuged at 400×g for 5 min. Then, cells were resuspended in the staining buffer and stained with antibodies on ice for 30 min in dark. Use the following antibodies: FITC anti‐mouse CD86 Antibody (105005, BioLegend, 1:200); APC anti‐mouse CD206 (MMR) Antibody (141707, BioLegend, 1:200); PE/Cyanine7 anti‐mouse CD163 Antibody (155319, BioLegend, 1:200); PE/Cyanine7 anti‐mouse TCR‐β chain (109221, BioLegend, 1:200); FITC anti‐mouse CD3 (100203, BioLegend, 1:200); FITC anti‐mouse CD8a (100705, BioLegend, 1:200); APC/Cyanine7 anti‐mouse CD8a (100713, BioLegend, 1:200); PE anti‐human/mouse Granzyme B (GZMB) (372207, BioLegend, 1:200); APC anti‐mouse CD279 (PD‐1) (135209, BioLegend, 1:200); Brilliant Violet 421 anti‐mouse CD366 (Tim‐3) (119723, BioLegend, 1:200). Flow cytometry was performed using an LSRFortessa instrument (BD Biosciences) and analyzed using FlowJo software (v10.8.1).

### 5.7 Chromatin Immunoprecipitation‐qPCR

Chromatin immunoprecipitation (ChIP) assays were performed using the SimpleChIP Enzymatic Chromatin IP Kit (Cell Signaling Technology) following the manufacturer's protocol. Briefly, 1 × 10⁷ iBMDM cells were cross‐linked, lysed, and enzymatically digested to obtain chromatin fragments. Chromatin extracts were immunoprecipitated with mouse monoclonal anti‐AhR antibody (sc‐133088, Santa Cruz) or mouse IgG (negative control). The immunoprecipitated DNA was purified for subsequent analysis by qPCR. Quantitative data were obtained as the ratio to input DNA. Primers used here were listed in Table S4.

### 5.8 Targeted Metabolomics

Targeted metabolomics for 44 Trp‐pathway metabolites using LC‐MS/MS was carried out by ExionLC ADsystem (SCIEX) coupled with a QTRAP 6500+ mass spectrometer (SCIEX) in Novogene Co. (Beijing, China). Briefly, conditioned medium from mCAFs‐vector, mCAFs‐TDO2, mCAFs‐TDO2 co‐cultured with iBMDMs (1:1 ratio) was used for subsequent metabolomics analysis. Then, samples were separated on a Xselect HSS T3 (2.1 × 150 mm, 2.5 µm) column with a 20 min gradient using 0.1% formic acid in water and 0.1% formic acid in acetonitrile as mobile phases. Data were acquired in both positive and negative multiple reaction monitoring (MRM)modes, and metabolite identification was based on Novogene's in‐house database. Data processing was performed using SCIEX OS software, and metabolites were annotated with KEGG and HMDB database. The list of metabolites used in vitro was as follows: 3‐Indoleacetic acid (I10008, Psaitong), 3‐Indolepropionic acid (220027, Sigma‐Aldrich), Indole‐3‐ethanol (W010155, MedChemExpress), 5‐Hydroxytryptophol (W041019, MedChemExpress), Indole‐3‐carboxylic acid (T5984, TargetMol), Indole (I104726, Aladdin), Indole‐3‐lactic acid (HY‐113099, MedChemExpress), Formyl‐N‐acetyl‐5‐methoxykynurenamine (AFMK) (T41345, TargetMol), Indole‐3‐carboxaldehyde (T8105, TargetMol), and L‐Kynurenine (S5839, Selleck).

### 5.9 Animal Experiments

All animal maintenance and operational procedures were carried out in accordance with the animal ethical agreement (No.AEE24060) approved by the Animal Care and Ethics Committee of Dalian Medical University. The 6‐ to 8‐week‐old male C57BL/6 mice were purchased from Beijing Vital River Laboratory Animal Technology and housed in a specific pathogen‐free environment with a 12/12 h day/night cycle. In all animal experiments, the maximum tumor volume permitted by the ethics committee is 1500 mm^3^ and this limit was not exceeded in our study. Tumor volume in mm^3^ was calculated using the following formula: tumor volume = 0.52 × L × W^2^, where L is the longest dimension and W is the perpendicular dimension.

In Experiment 1, LLC cells expressing luciferase reporter, mCAFs‐vector or mCAFs‐TDO2 cells, and iBMDMs (ratio 1:1:1) were seeded in 100 mm dishes and co‐cultured. After 3 days, the cells of direct co‐culture system were harvested and resuspended in 100 µL 40% Matrigel in PBS, which were implanted into the left lung of C57BL/6 mice. In vivo imaging observation was performed 3 weeks after the implantation, before the mice were sacrificed, and tumors were photographed. At the end points, tumor‐bearing mice were euthanized, and tissues were collected and fixed in 4% paraformaldehyde. Paraffin‐embedded lung samples were subjected to H&E, IHC staining, and multiplexed IHC staining. IHC staining was performed with following antibodies: anti‐Ki‐67 (27309‐1‐AP, proteintech, 1:500), anti‐CD86 (15880‐1‐AP, proteintech, 1:300), anti‐CD206 (#24595, CST, 1:400), anti‐CD163 (ab182422, Abcam, 1:500), and anti‐CD8 (ab316778, Abcam, 1:500), anti‐PD‐1 (#84651, CST, 1:100), anti‐Granzyme B (ab255598, Abcam, 1:200). Multiplexed IHC was performed with primary antibodies: anti‐Pan‐Keratin (4545, CST, 1:1000), anti‐TDO2 (15880‐1‐AP, proteintech, 1:300), anti‐IL4I1 (ab317248, Abcam, 1:1000), anti‐COL1A1 (72026, CST, 1:200), and anti‐F4/80 (70076, CST, 1:500). For each specimen, whole‐slides were scanned using Pannoramic MIDI platform.

In Experiment 2, murine Kras^G12D^Tp53^−/−^ (KP) cells, TDO2‐overexpressing or vector control mCAFs, and iBMDMs were co‐cultured at a 1:1:1 ratio in 100 mm dishes for 72 h. The mixed cells were then collected, resuspended in 50 µL of 50% Matrigel in PBS, and subcutaneously injected into the right flanks of C57BL/6 mice. On day 9 post‐implantation, mice were randomly divided into four groups (*n* = 5 per group) and intraperitoneally administered either IgG2α isotype control (BE0089, RRID: AB_1107769, BioXcell) or anti‐PD‐1 antibody (200 µg, BE0146, RRID: AB_10949053, BioXcell) every three days, corresponding to the following treatment groups: (1) mCAF‐vector + iBMDMs + IgG2α, (2) mCAF‐vector + iBMDMs + anti‐PD‐1, (3) mCAF‐TDO2 + iBMDMs + IgG2α, and (4) mCAF‐TDO2 + iBMDMs + anti‐PD‐1. On day 21, all mice were euthanized, and tumors were harvested for volume measurement and subsequent flow cytometry analyses.

In Experiment 3, KP cells, mCAFs‐TDO2, and iBMDMs were co‐cultured at a 1:1:1 ratio in 100 mm dishes for 72 h. The cell mixture was harvested, resuspended in 50 µL of 50% Matrigel in PBS, and subcutaneously implanted into the right flanks of C57BL/6 mice. On day 9 post‐implantation, tumor‐bearing mice were randomly assigned to eight groups (*n* = 6 per group) to receive various combinations of anti‐IgG2α/anti‐PD‐1 (200 µg, BE0146, BioXcell), DMSO/RSL3 (30 mg/kg, S8155, Selleck), DMSO/IKE (30 mg/kg, S8877, Selleck), and PBS/CH‐223191 (10 mg/kg, S7711, Selleck), corresponding to the following treatment groups: (1) IgG2α + DMSO + PBS, (2) IgG2α + IKE/RSL3 + PBS, (3) IgG2α + DMSO + CH‐223191, (4) anti‐PD‐1+ DMSO + PBS, (5) IgG2α + IKE/RSL3 + CH‐223191, (6) anti‐PD‐1+ IKE/RSL3 + PBS, (7) anti‐PD‐1 + DMSO + CH‐223191, and (8) anti‐PD‐1 + IKE/RSL3 + CH‐223191. Anti‐PD‐1 and IKE/RSL3 were administered via intraperitoneal injection, while CH‐223191 was given via oral gavage. Anti‐PD‐1 was administered every three days, whereas IKE/RSL3 and CH‐223191 were administered daily. On day 24, mice were euthanized for sample collection. Tumor tissues were analyzed by histological staining and flow cytometry analyses. Additionally, peripheral blood was collected for the evaluation of potential hepatotoxicity (AST and ALT) and cardiotoxicity (L‐LDH).

### 5.10 Survival Analysis

Survival analysis was performed using the R packages “survminer” (v3.1‐8) and “survival” (v3.1‐8). Patients within all datasets were divided into two groups based on the best‐separation cut‐off value determined by the “surv_cutpoint” function to plot the Kaplan–Meier survival curves, and *P*‐value was calculated using a log‐rank test. Univariate Cox proportional models were first used to analyze associations between the clinical parameters and OS, among which the parameters with statistical significance were further included in a multivariate Cox regression analysis. *P*‐value < 0.05 was considered statistically significant.

### 5.11 Statistical Analyses

All statistical analyses and graphical presentations were performed using open‐sourced R (v4.2.2) or GraphPad Prism software (v10.0). Data were expressed as mean ± standard error of the mean (SEM). As appropriate, statistical significance was determined using either the Student's *t*‐test or the Wilcoxon rank‐sum test. Multiple comparisons following one‐way analysis of variance (ANOVA) with Tukey's post‐hoc test and the Kruskal–Wallis test were performed. Before the comparisons, the normality of the distributions was tested with the Shapiro‐Wilk test. Correlation analysis was created with Pearson's correlation. The statistical tests used in the figures are specified in the figure legends, and statistical significance was set at a *P*‐value < 0.05.

## Funding

This work was supported by grants from the National Natural Science Foundation of China (No. 81803886, and 81774078) to Chundong Gu, the Dalian Science and Technology Innovation Fund (No.2022JJ12SN044) to Chundong Gu, and the Dalian Key Medical Specialty Dengfeng Project (No.2022DF040) to Shilei Zhao.

## Conflicts of Interest

The authors declare no conflict of interest.

## Supporting information




**Supporting File 1**: advs73373‐sup‐0001‐SuppMat.docx.

**Supporting File 2**: advs73373‐sup‐0002‐Supplementary Tables.xls.

## Data Availability

The spatial transcriptomics generated in this study have been deposited in the GSA database under accession code HRA009412. The murine metabolomics data can be accessed in OMIX under project OMIX013839. All data utilized for this study are publicly published and accessible, with detailed access links provided in the public datasets collection section. Additionally, all analysis code generated is available from the authors upon request.
