# Supplementary material for: IL4I1⁺ Macrophages and TDO2⁺ Myofibroblasts Drive AhR‐Mediated Immunosuppression and Ferroptosis Resistance in Solid Predominant Lung Adenocarcinoma
Source: Adv Sci (Weinh). 2025 Dec 22;13(13):e13606. doi: 10.1002/advs.202513606 (PMC12955909; doi:10.1002/advs.202513606)
Supplement: Supplementary file 1 — Supporting File 1: advs73373‐sup‐0001‐SuppMat.docx. [file ADVS-13-e13606-s001.pdf]

Supporting Information

Title

IL4I1$^+$ Macrophages and TDO2$^+$ Myofibroblasts Drive AhR-Mediated Immunosuppression and Ferroptosis Resistance in Solid Predominant Lung Adenocarcinoma

Zhaoxuan Wang, Weijiao Xu, Lei Zhao, Lin Zhong, Wendan Yu, Shengmin Wang, Lu Sun, Tao Guo, Fengzhou Li, Zhuoshi Li, Lei Fang, Shiqing Wang, Guohui Zhang, Guoqing Xue, Wei Guo*, Shilei Zhao*, Chundong Gu**

Supplemental Figures and figure legends

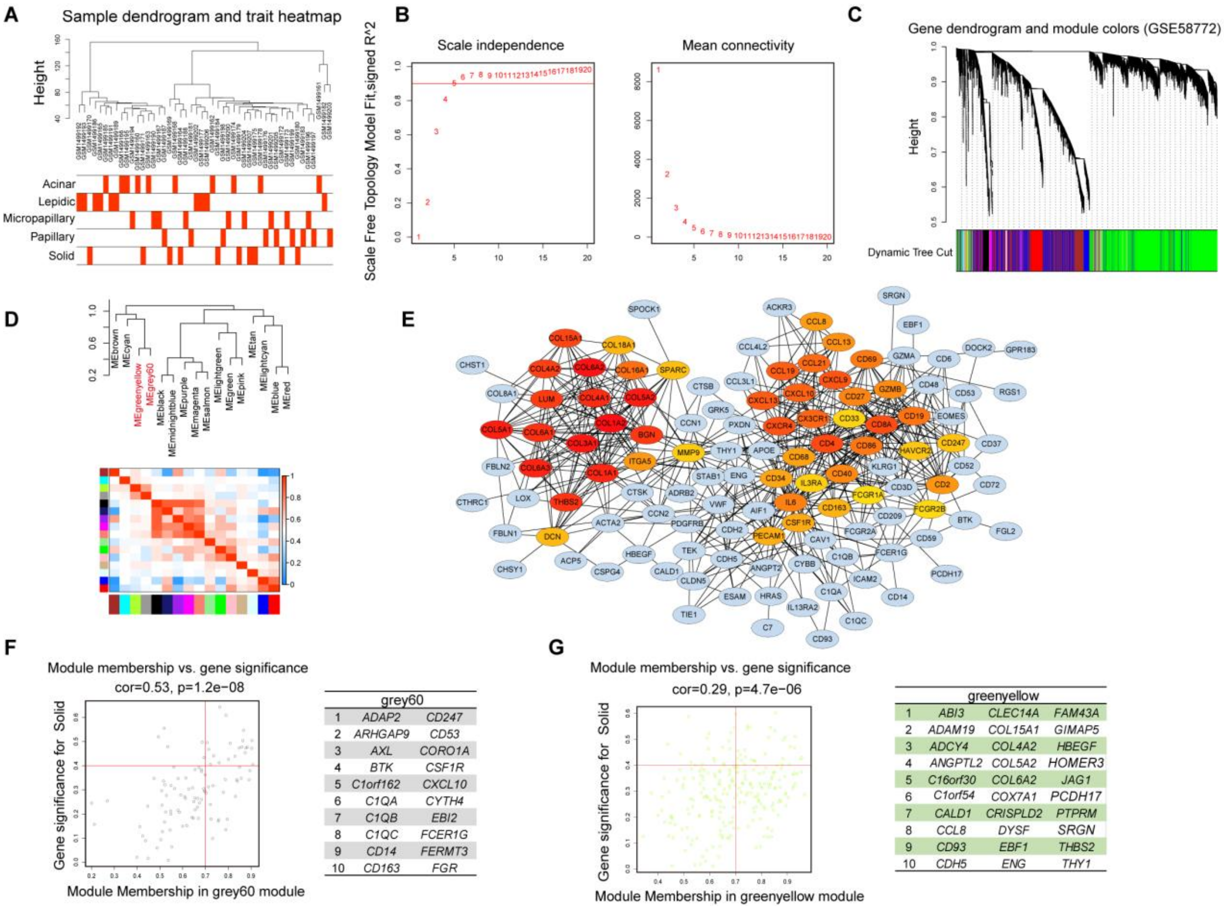

Figure S1. WGCNA analysis identified gene modules relevant to solid pattern in GSE58772. A. The sample dendrogram and trait heatmap from WGCNA analysis (n=48). **B.** Analysis of network topology for soft powers to identify the threshold best fit in the scale-free network. A soft power of 5 was selected to meet the threshold of 0.90. **C.** The gene dendrogram of genome-wide transcriptome and matched module colors in WGCNA analysis. **D.** Clustering dendrogram based on ME correlation. Heatmap of 17 module communities organized by module related order. **E.** Cytohubba showing the protein-protein network in the grey60 module and greenyellow module. **F and G.** Scatter plot showing the relationship between gene significance and module membership in the grey60 module and greenyellow module (GS > 0.4 and MM > 0.7).

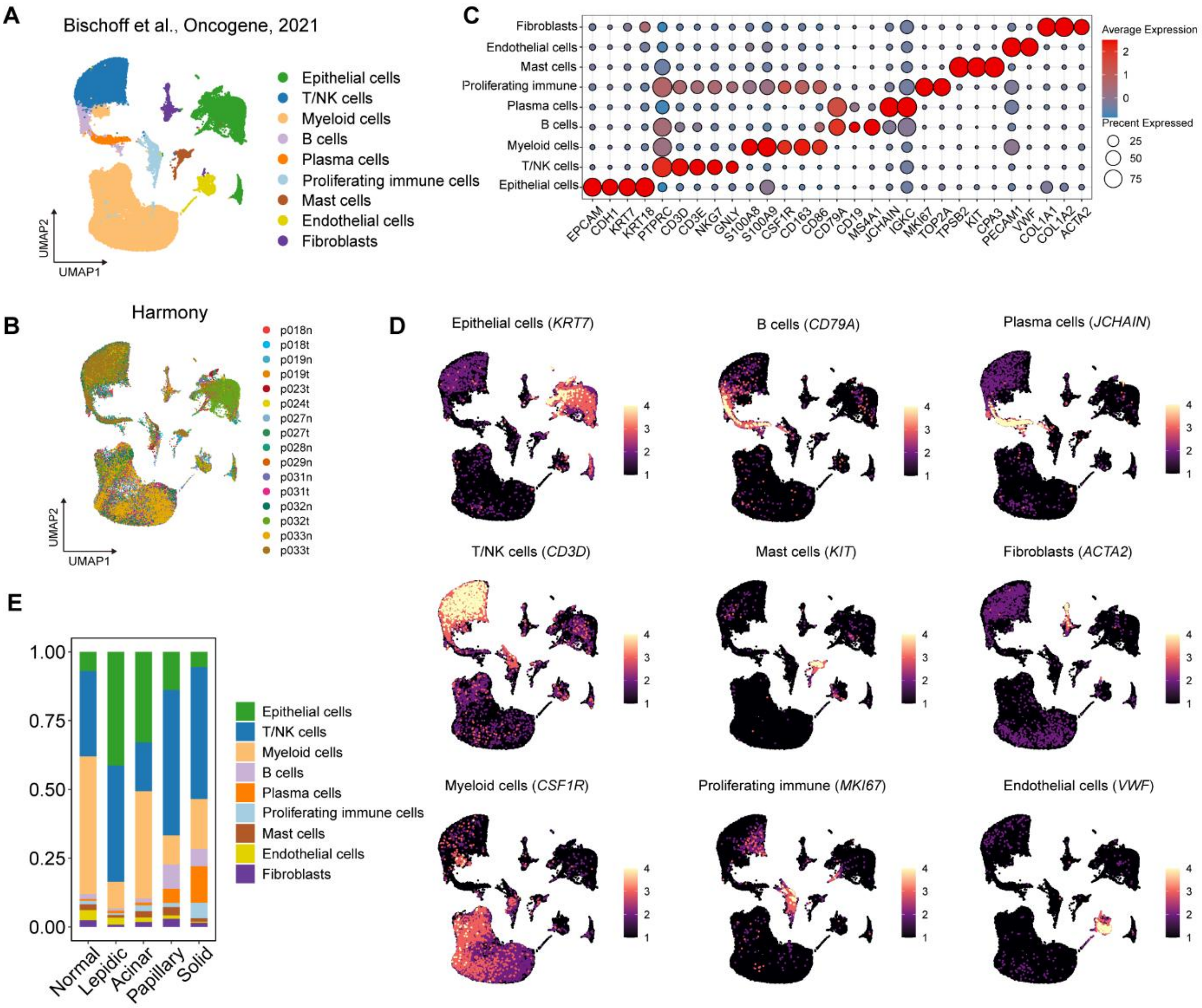

Figure S2. scRNA-seq analysis revealed major cell types from Bischoff et al.'s study. A. UMAP plots showing the major cell types. **B.** UMAP plots showing sample enrichment in the harmony embedding. **C.** Bubble heatmap showing the marker genes expression for the major cell types. Dot size indicates the fraction of expressing cells, colored based on normalized expression levels. **D.** UMAP plots showing the marker gene expression for major cell types including *KRT7*, *CD79A*, *JCHAIN*, *CD3D*, *KIT*, *ACTA2*, *CSF1R*, *MKI67*, and *VWF*. **E.** Stacked histogram showing the frequency of major cell types across histologic subtypes including normal, lepidic, acinar, papillary, and solid.

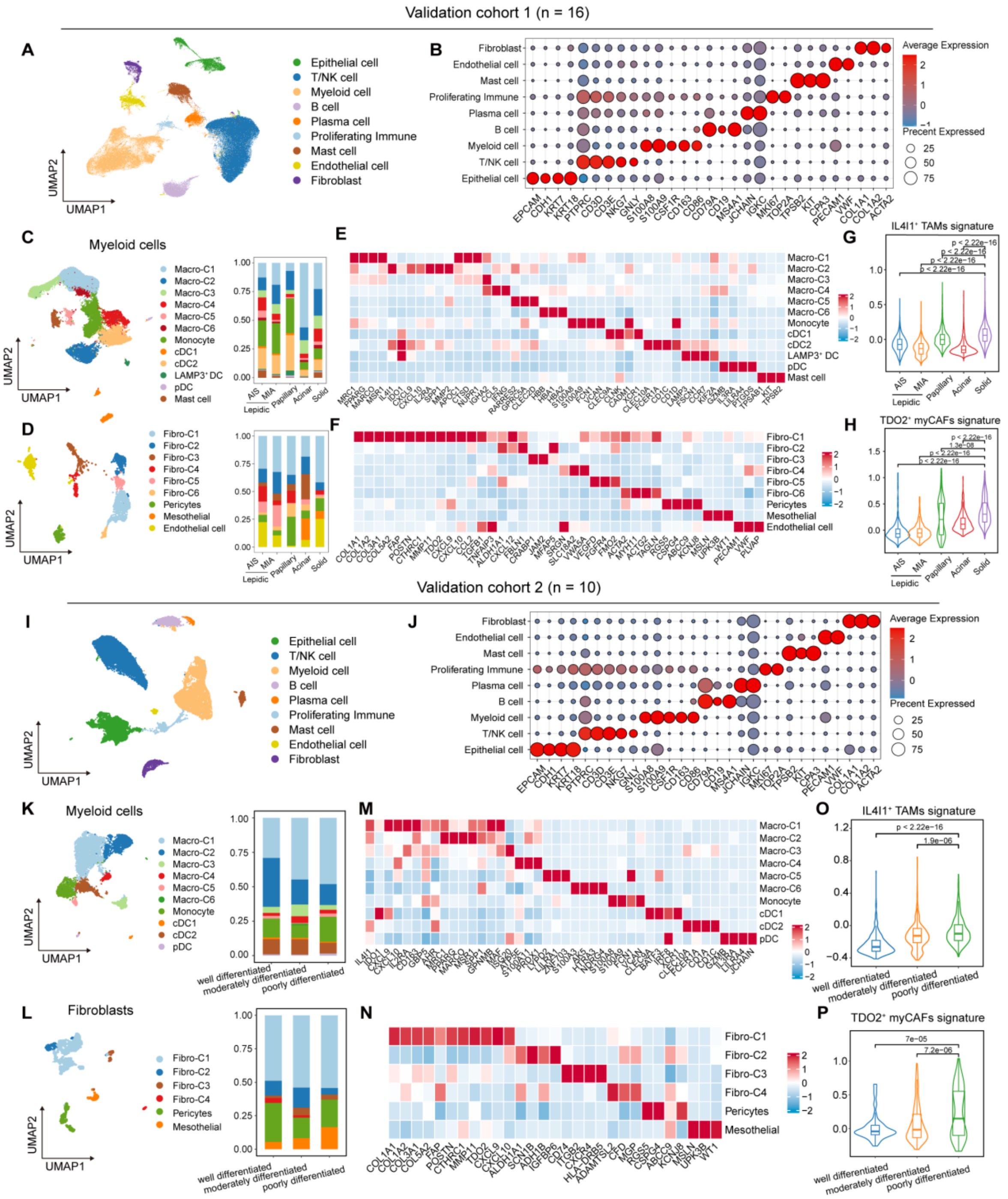

Figure S3. Cellular composition and marker expression profiles in two validation LUAD cohorts. **A**. UMAP plots showing cell types in validation cohort 1 (n=16). **B.** Bubble heatmap showing the marker genes expression for the major cell types. Dot size indicates the fraction of expressing cells, colored based on normalized expression levels. **C-D.** UMAP plots showing myeloid cell **(C)** and fibroblast **(D)** subtypes in validation cohort 1. **E-F.** Heatmaps showing expression profiles in myeloid cell **(E)** and fibroblast **(F)** subpopulations in validation cohort 1. **G-H.** Violin plots showing the enrichment of IL4I1^{+} TAMs signature **(G)** and TDO2^{+} myCAFs signature **(H)** within macrophage and fibroblast subpopulations in validation cohort 1. P-values were calculated with Wilcoxon test. **I**. UMAP plots showing cell types in validation cohort 2 (n=10). **J.** Bubble heatmap showing the marker genes expression for the major cell types. Dot size indicates the fraction of expressing cells, colored based on normalized expression levels. **K-L.** UMAP plots showing myeloid cell **(K)** and fibroblast **(L)** subtypes in validation cohort 2. **M-N.** Heatmaps showing expression profiles in myeloid cell **(M)** and fibroblast **(N)** subpopulations in validation cohort 2. **O-P.** Violin plots showing the enrichment of IL4I1^{+} TAMs signature **(O)** and TDO2^{+} myCAFs signature **(P)** within macrophage and fibroblast subpopulations in validation cohort 2. P-values were calculated with Wilcoxon test.

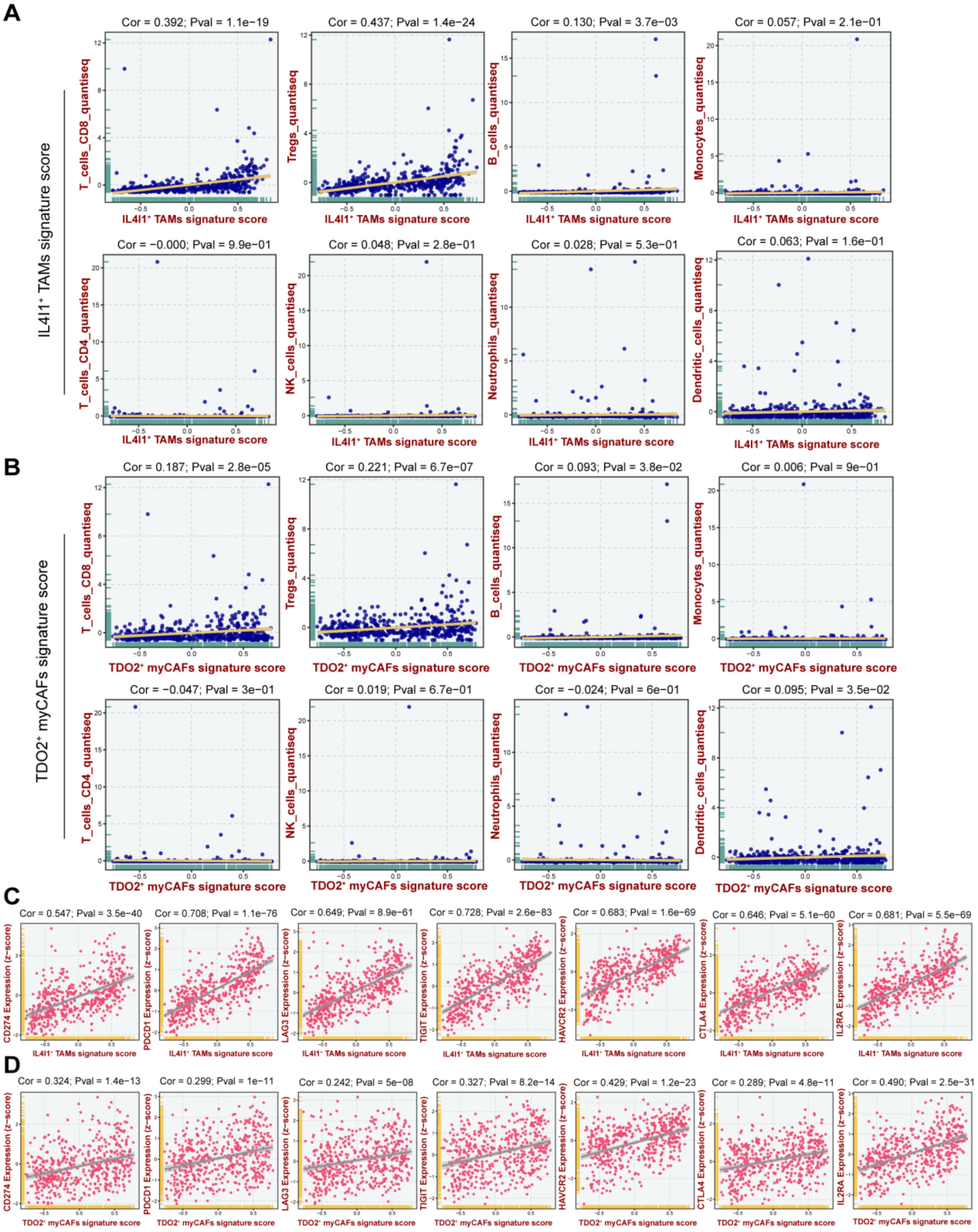

Figure S4. Correlation analysis revealing the association between Macro-C3 and myCAF-C2 and immune characteristics in LUAD. A. Correlation plots between Macro-C3 (IL4I1^{+} TAMs) signature score and immune cell abundance using quantiseq (CD8^{+} T cells, CD4^{+} T cells, Treg, NK cells, B cells, neutrophils, monocytes, and dendritic cells). **B.** Correlation plots between myCAF-C2 (TDO2^{+} myCAFs) signature score and immune cell abundance (CD8^{+} T cells, CD4^{+} T cells, Treg, NK cells, B cells, neutrophils, monocytes, and dendritic cells). **C.** Correlation plots between Macro-C3 (IL4I1^{+} TAMs) signature score and co-inhibitory immune checkpoints. **D.** Correlation plots between myCAF-C2 (TDO2^{+} myCAFs) signature score and co-inhibitory immune checkpoints. Statistical significance was assessed using Pearson's correlation test.

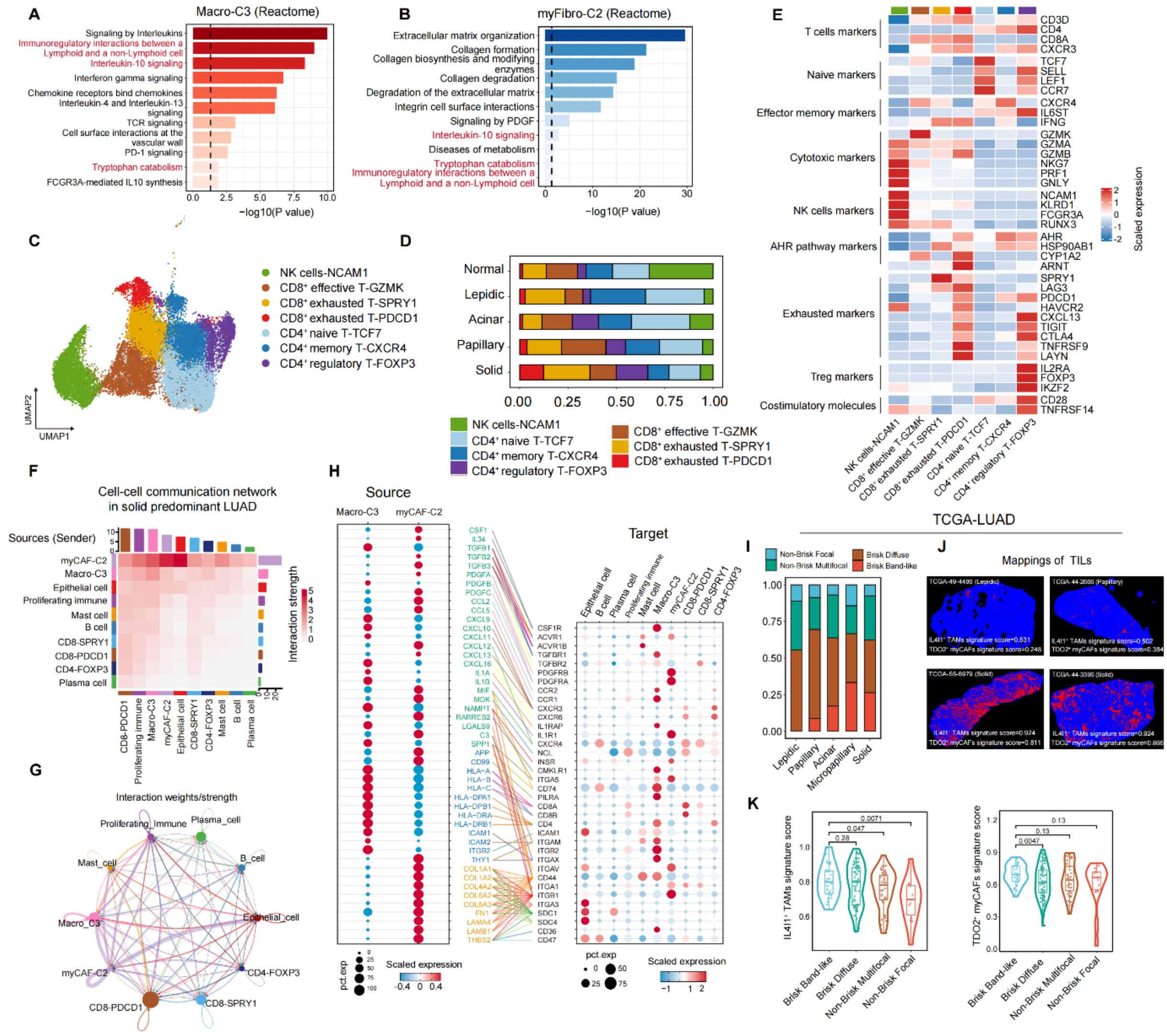

Figure S5. Functional characteristics and cell-cell communications in Macro-C3 and myCAF-C2. A-B. The barplot showing the functional enrichment in Macro-C3 (IL4I1^{+} TAMs) and myCAF-C2 (TDO2^{+} myCAFs) based on Reactome database. **C.** UMAP plots showing T and NK cell subpopulations in the scRNA-seq from Bischoff et al.'s study. **D.** Stacked histogram showing the frequency of T and NK cell subpopulations across histologic subtypes. **E.** Heatmap showing the expression level of specific markers across T and NK cell subpopulations. **F.** Heatmap showing interaction strengths based on ligand-receptor interaction scores using CellChat in solid predominant LUAD. **G.** Chord diagram showing interaction strengths based on ligand-receptor interaction scores using CellChat in solid predominant LUAD. **H.** Dot plots showing gene expression levels of receptor-ligand pairs involved in interactions between different clusters in solid predominant LUAD. **I.** Stacked histogram showing the tumor-infiltrating lymphocytes (TILs) subtypes across histologic subtypes in TCGA-LUAD cohort based on deep learning for H&E images by Saltz et al.'s study. **J.** Representing images of TILs subtypes across histologic subtypes and matched signature scores of Macro-C3 (IL4I1^{+} TAMs) and myCAF-C2 (TDO2^{+} myCAFs). **K.** The violin plot showing signature scores of Macro-C3 (IL4I1^{+} TAMs) and myCAF-C2 (TDO2^{+} myCAFs) across TILs subtypes. P-values were calculated by the Student's t test.

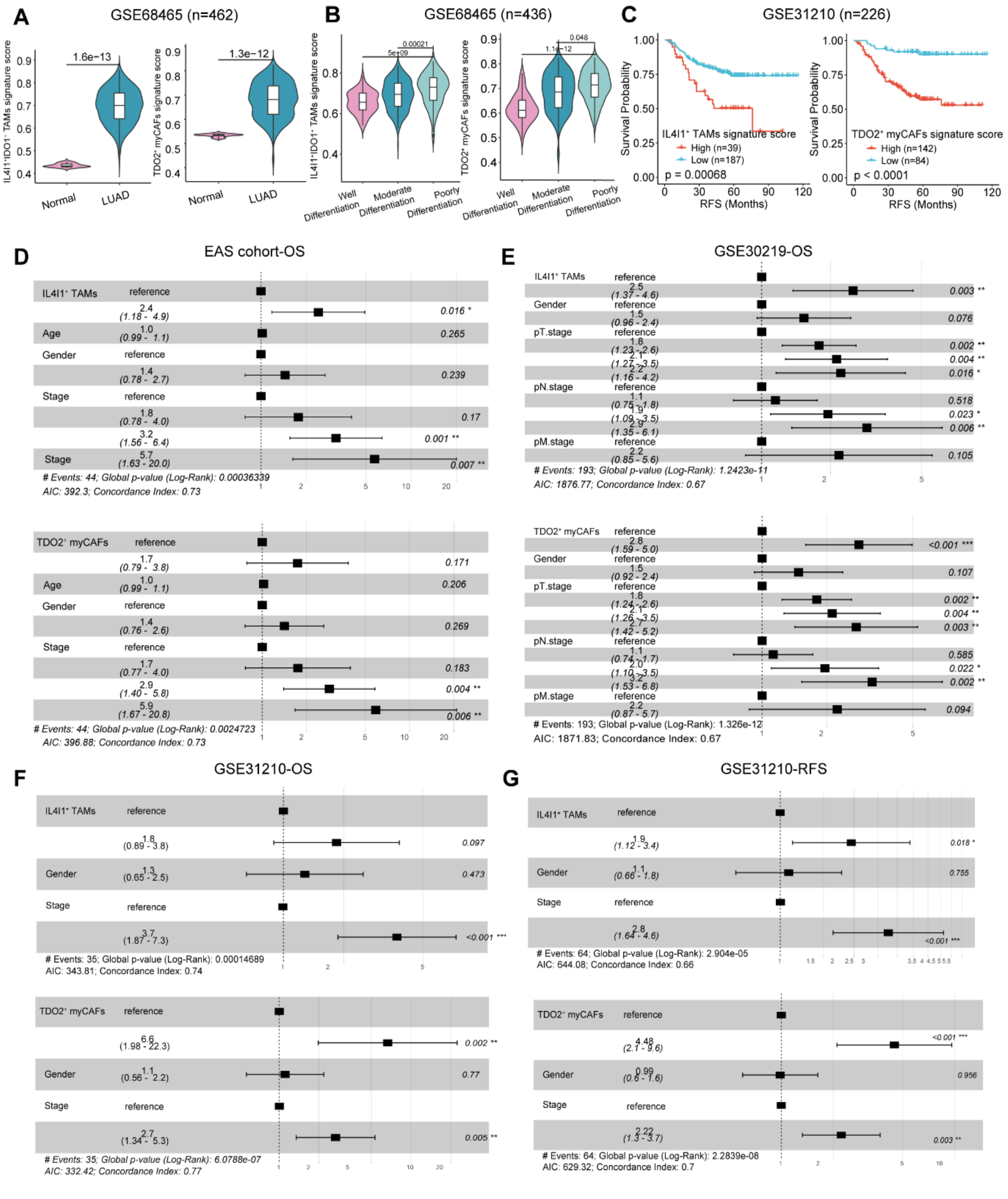

Figure S6. Prognostic value of IL4I1⁺ TAMs and TDO2⁺ myCAFs signature scores in multiple LUAD cohorts. A. Violin plot showing signature scores of IL4I1⁺ TAMs and TDO2⁺ myCAFs between tumor samples and normal samples in GSE68465. P-values were calculated with Wilcoxon test. **B.** Violin plot showing signature scores of IL4I1⁺ TAMs and TDO2⁺ myCAFs across histological grades in GSE68465. P-values were calculated with Wilcoxon test. **C.** Kaplan-Meier survival curves showing recurrence-free survival (RFS) in GSE31210 (n=226) stratified by IL4I1⁺ TAM (left) and TDO2⁺ myCAF (right) signature scores (high vs. low groups). **D-E.** Forest plots showing hazard ratios (HR) from multivariate Cox regression analysis for overall survival (OS) in EAS cohort (**D**) and GSE30219 (**E**). **F-G**. Forest plots showing HR from multivariate Cox regression analysis of OS (**F**) and RFS (**G**) in GSE31210. *P-value < 0.05, ** P-value < 0.01, *** P-value < 0.001.

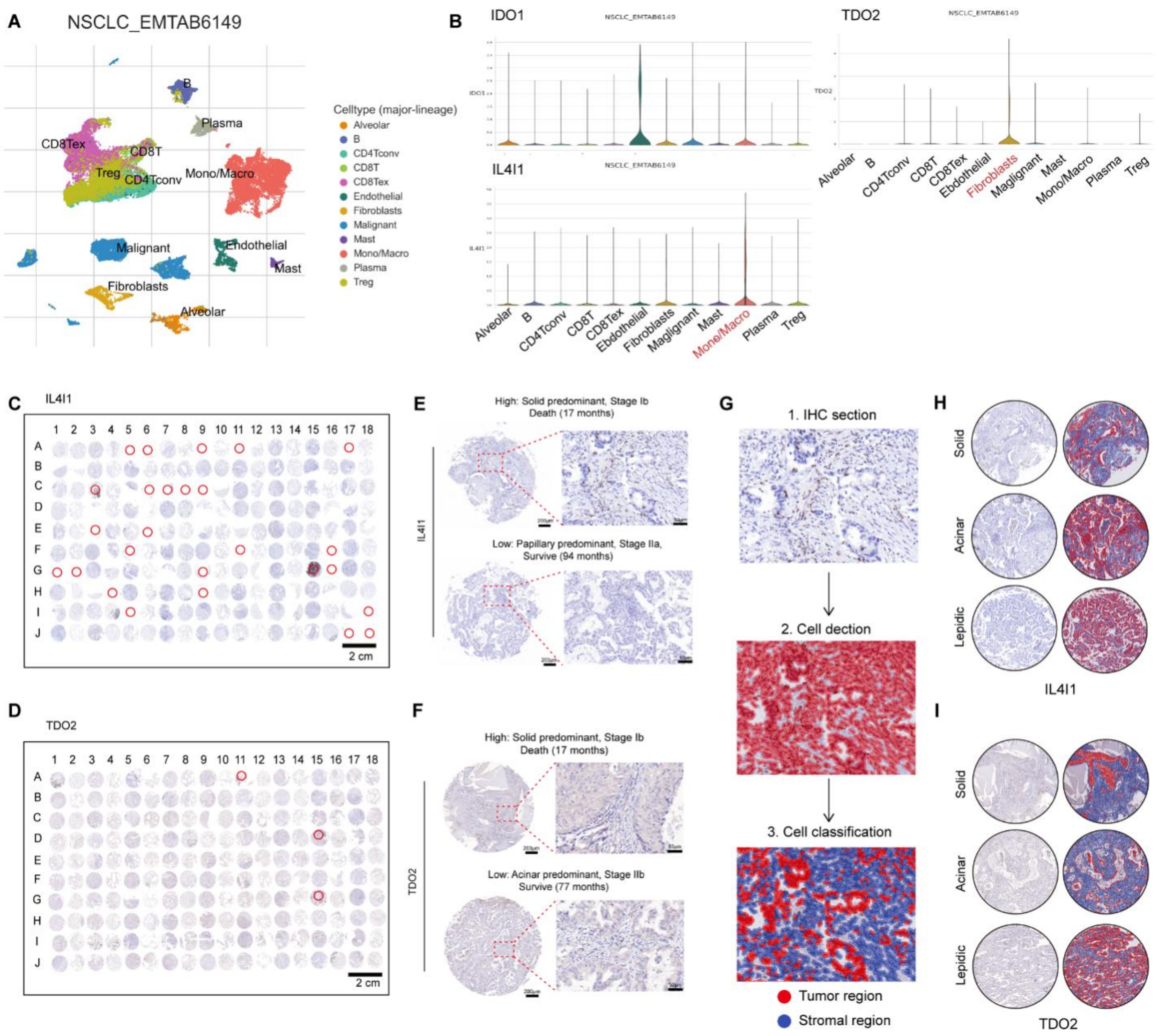

Figure S7. IL4I1 and TDO2 expression profile in scRNA-seq and tissue microarray of LUAD. A. UMAP plot showing scRNA-seq clustering in NSCLC-EMTAB6149. **B**. Violin plots showing the expression levels of IDO1, TDO2, and IL4I1 across different cell types in NSCLC-EMTAB6149. IL4I1 is predominantly expressed in Mono/Macro subsets, while TDO2 is enriched in fibroblasts. **C-F.** Overall staining of IL4I1 and TDO2 in the tissue microarray. The microarray contained 180 samples, and the detached spots (red circles) were excluded, leaving IL4I1 (n=154, tumor n=74, normal n=80) and TDO2 (n=177, tumor n=87, normal n=90) retained for analysis. **G.** The image-processing pipeline used to recognize LUAD tumor region and stromal region based on QuPath software. **H-I.** Spatial distribution of IL4I1 (**H**) and TDO2 (**I**) expression in LUAD tissues with distinct histological subtypes (lepidic, acinar, and solid), with tumor (red) and stromal (blue) regions mapped separately.

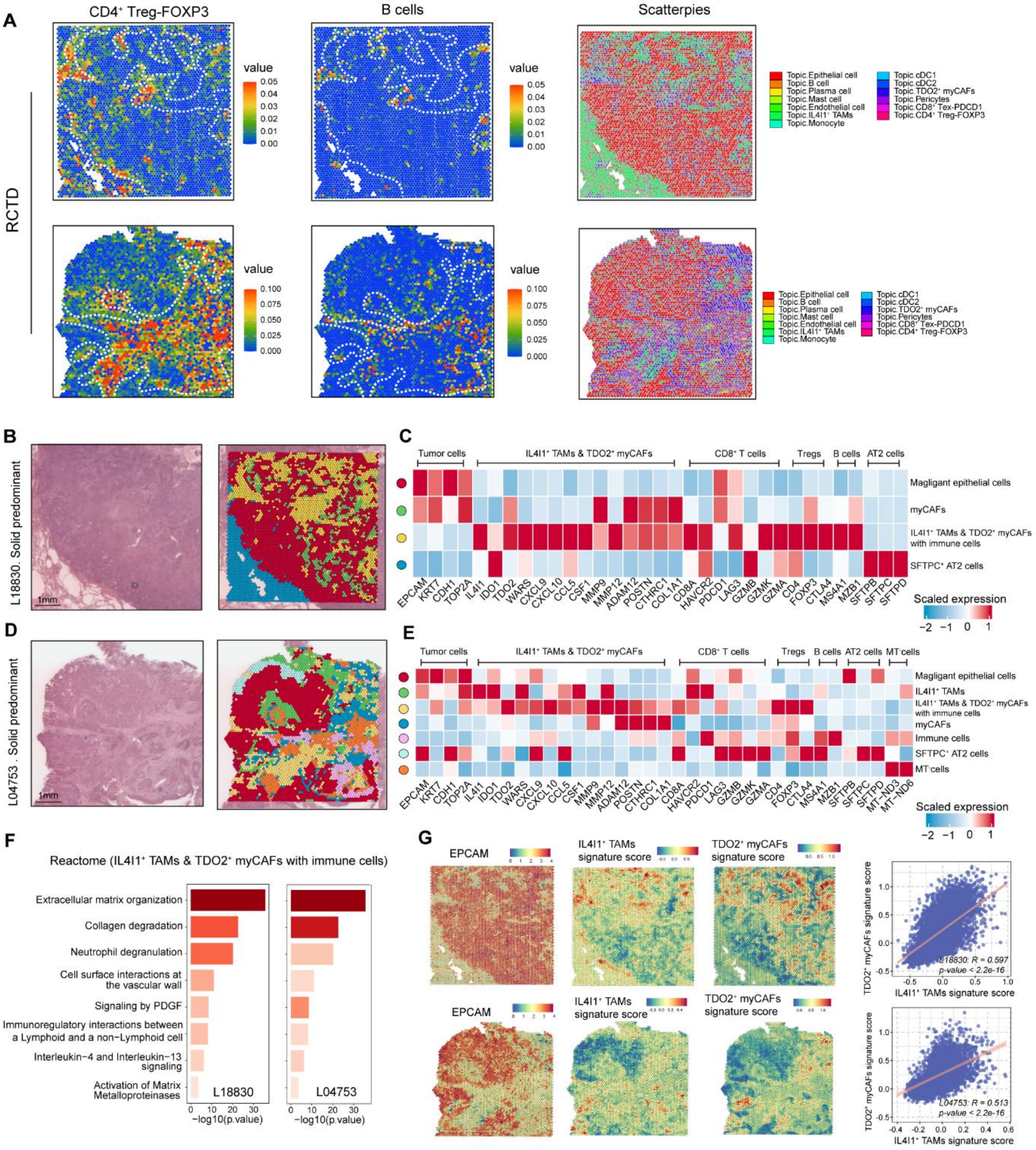

Figure S8. Spatial profiles of solid predominant LUAD. A. Deconvolution of spatial transcriptomics data using RCTD showing spatial distribution of $CD4^+$ Treg-FOXPE and B cells in solid predominant LUAD samples (n=2). Scatter pie plots depict spatially resolved cell-type proportions, highlighting enrichment of $IL4I1^+$ TAMs and $TDO2^+$ myCAFs in the tumor stroma. **B.** Representative H&E staining (left) and cell-type annotation (right) in LUAD samples L18830. **C.** Scaled expression of selected marker genes across annotated cell types in L18830. **D.** Representative H&E staining (left) and cell-type annotation (right) in LUAD samples L04753. **E.** Scaled expression of selected marker genes across annotated cell types in L04753. **F.** Reactome pathway enrichment analysis illustrating biological processes associated with $IL4I1^+$ TAMs and $TDO2^+$ myCAFs. **G.** Spatial correlation analysis showing co-localization of $IL4I1^+$ TAM and $TDO2^+$ myCAF signature scores. Statistical significance was assessed using Pearson's correlation test.

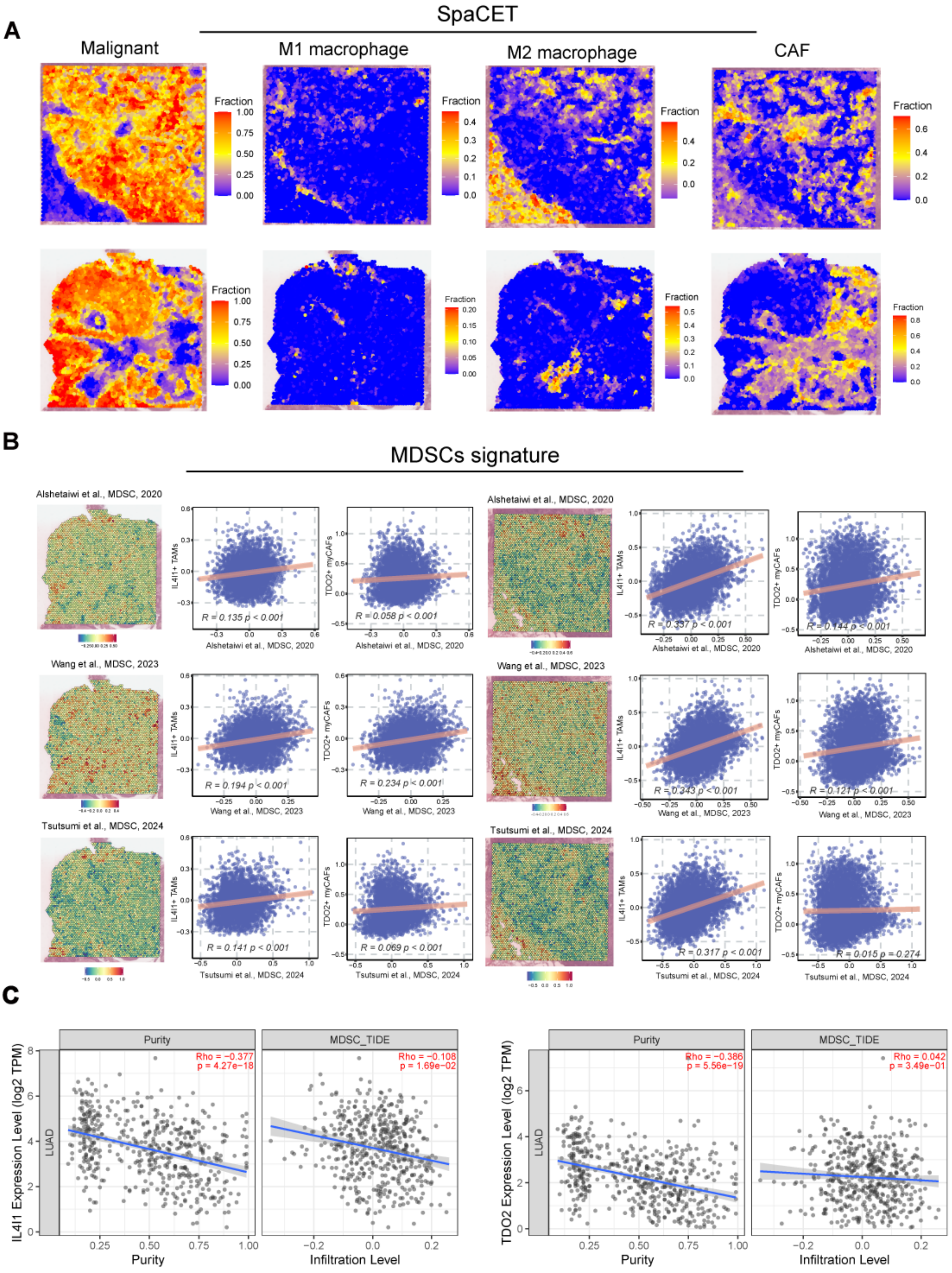

Figure S9. Spatial Mapping and Myeloid Context of IL4I1^{+} TAMs and TDO2^{+} myCAFs. **A.** Spatial distribution of malignant cells, M1 macrophage, M2 macrophage, and CAF using SpaCET. **B.** Association of MDSC enrichment with IL4I1^{+} TAMs and TDO2^{+} myCAFs referring to MDSC signature scores from multiple independent studies. Representative immune maps (left panels) and scatterplots show positive correlations (right panels). **C**. Correlation of *IL4I1* and *TDO2* mRNA expression with tumor purity and myeloid-derived suppressor cell (MDSC) infiltration levels (TIDE) in LUAD.

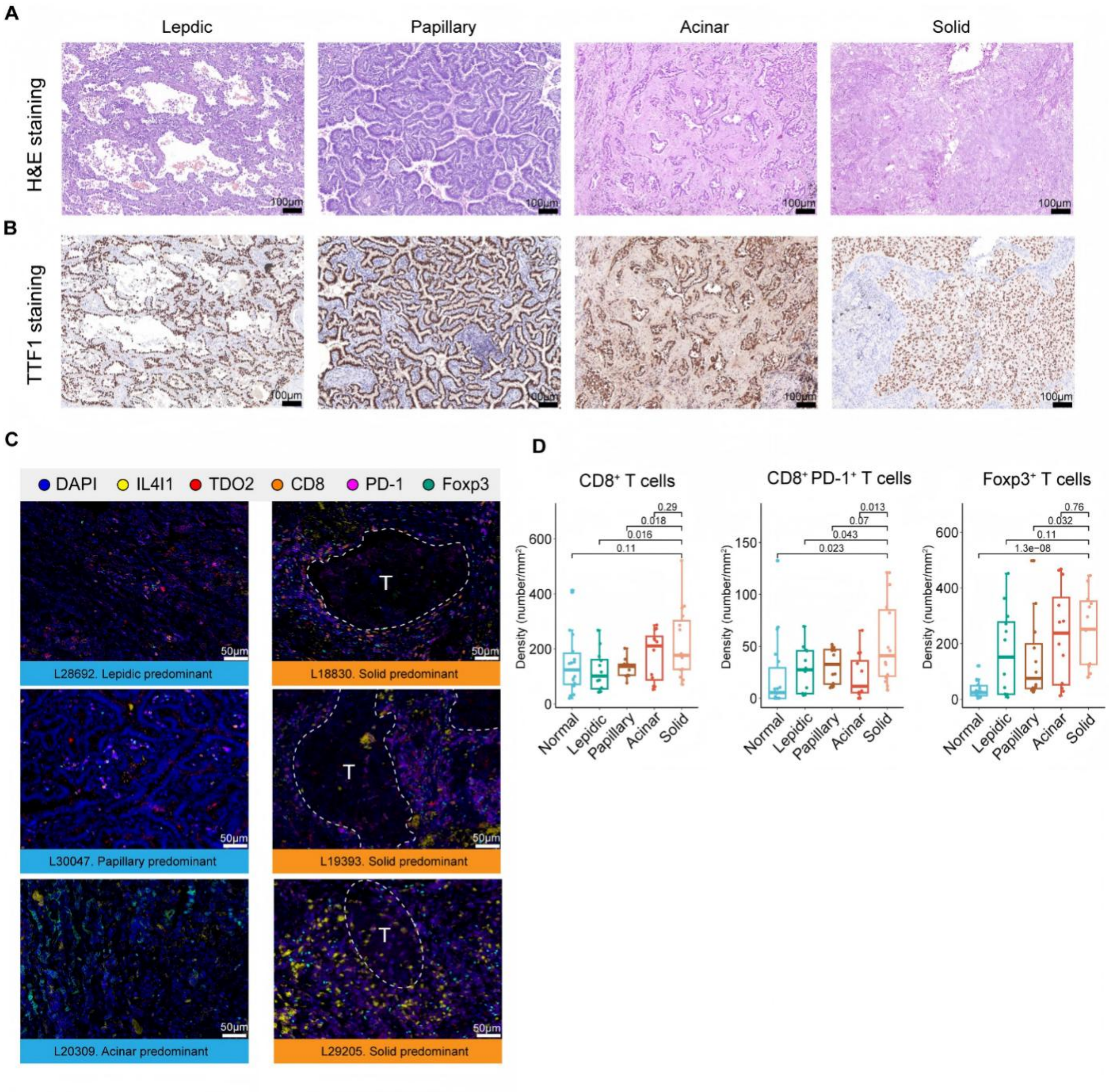

Figure S10. H&E, TTF1, and multiplexed IHC staining in LUAD. A. Representative H&E staining among histological subtypes. **B.** Representative TTF1 staining among histological subtypes. **C.** Representative multiplexed IHC staining of solid predominant LUAD samples and non-solid predominant LUAD samples stained for IL4I1, TDO2, CD8, PD-1, and Foxp3. **D.** Boxplots showing the density of $CD8^+$ T cells, $CD8^+$ $PD\text{-}1^+$ cells, and $Foxp3^+$ cells. P-values were calculated by Wilcoxon test.

A

Gene	Level of Comparison	Identity (%)	E-value
TDO2	Genomic DNA	83.09%	2×10^{-66}
	Protein	88.52%	≈ 0
IL4I1	Genomic DNA	89.92%	6×10^{-39}
	Protein	79.28%	≈ 0

B

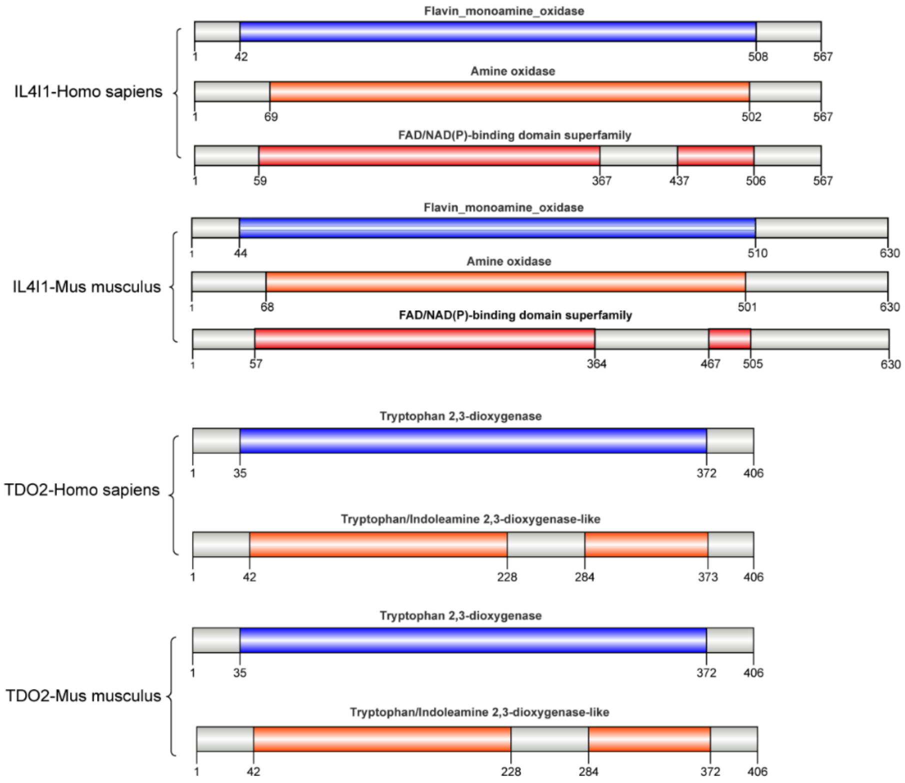

Figure S11. Conservation of human and murine TDO2 and IL4I1. A. BLAST-based homology analysis of TDO2 and IL4I1 between human and mouse. Both genes show high genomic and protein sequence identity, with TDO2 exhibiting 83.09% genomic and 88.52% protein identity, and IL4I1 showing 89.92% genomic and 79.28% protein identity. **B.** Conserved domain architecture of human and murine IL4I1 and TDO2 predicted by Pfam and InterPro. Both species display identical arrangements of key enzymatic domains, indicating preserved structural and functional features.

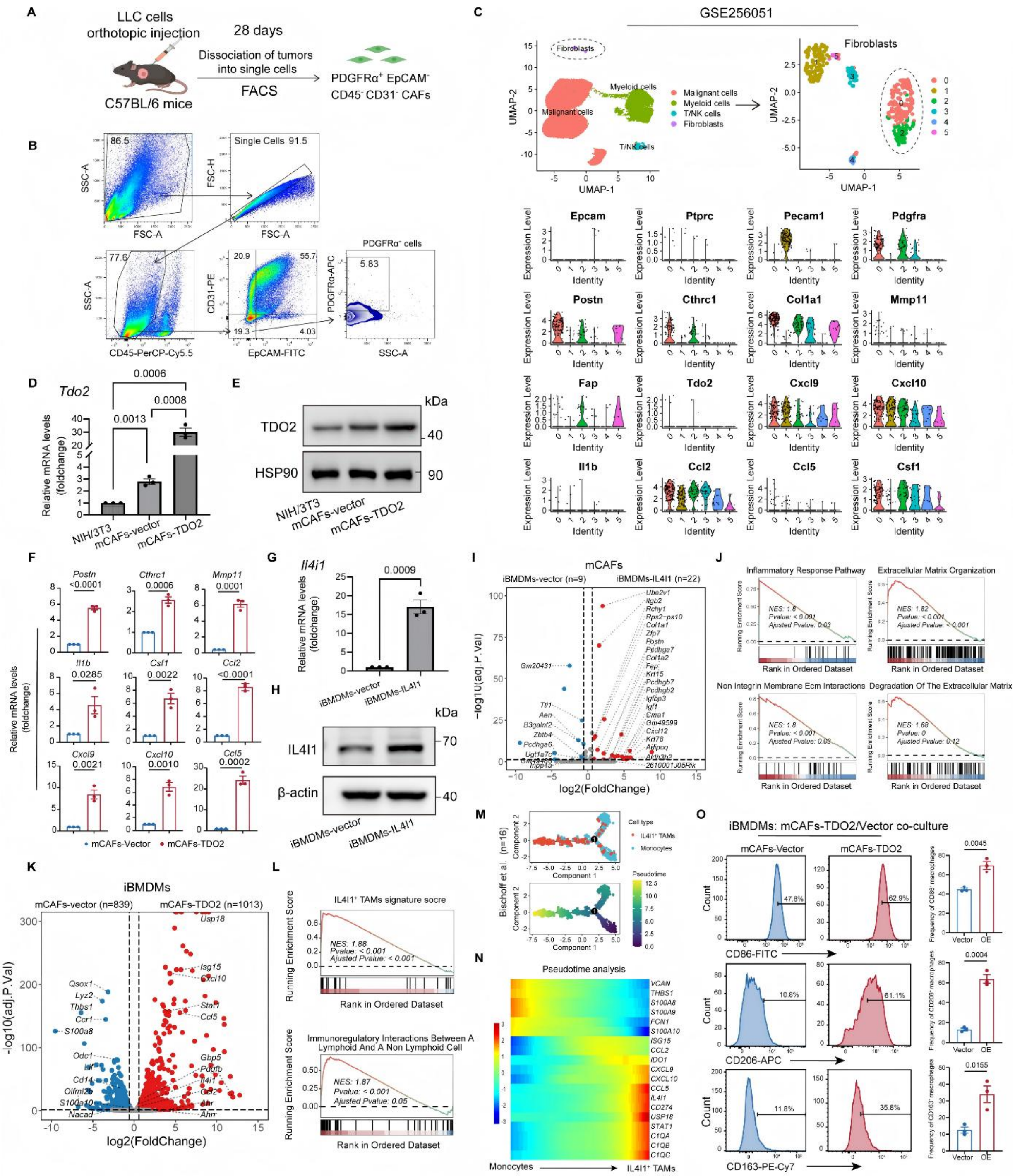

Figure S12. TDO2-overexpressing mCAFs promote IL4I1⁺ TAMs differentiation and immune remodeling. A. Schematic showing the workflow of mCAFs isolation from orthotopically implanted LLC tumors in C57BL/6 mice after 28 days. Tumors were dissociated into single cells and CAFs were sorted by FACS as PDGFRα⁺ EpCAM⁻ CD45⁻ CD31⁻ cells. **B.** Representative FACS gating strategy for identifying murine CAFs. Cells were first gated on singlets and live populations, then PDGFRα⁺ EpCAM⁻ CD45⁻ CD31⁻ murine CAFs were sorted. **C.** UMAP plots and violin plots showing expression of cell-type specific markers in GSE256051. **D**. RT-qPCR showing expression of *Tdo2* in mCAFs-TDO2 compared to vector control (n=3 per group). P-values were calculated with one-way ANOVA test with Tukey's post-hoc test. **E**. Western blotting showing TDO2 expression in NIH/3T3, mCAFs-Vector, and mCAFs-TDO2 cells. **F.** Bar plot showing the expression of *Postn*, *Cthrc1*, *Mmp11*, *Il1b*, *Csf1*, *Ccl2*, *Cxcl9*, *Cxcl10*, and *Ccl5 using* RT-qPCR from mCAFs-Vector/TDO2 cells (n=3 per group). P-values were calculated with Student's t-test. **G**. RT-qPCR showing expression of *Il4i1* in iBMDMs-IL4I1 compared to vector control (n=3 per group). P-values were calculated with Student's t-test. **H**. Western blotting showing IL4I1 expression in iBMDMs-Vector and iBMDMs-IL4I1. **I.** Volcano plot

showing differentially expressed genes (DEGs) in mCAFs co-cultured with iBMDMs-Vector and iBMDMs-IL4I1. **J.** GSEA analysis reveals enrichment in the extracellular matrix remodeling-related pathway and immune response pathway. **K**. Volcano plot showing DEGs in iBMDMs co-cultured with mCAFs-TDO2 versus mCAFs-Vector. **L.** GSEA analysis reveals enrichment in IL4I1^{+} TAM gene signatures and immune regulatory interaction terms. **M.** Pseudotime trajectory colored by pseudotime. **N.** Heatmap showing dynamic expression of key markers along pseudotime trajectory. **O.** Flow cytometry analysis of co-cultured iBMDMs with mCAFs-TDO2 reveals increased proportions of CD206^{+} and CD163^{+} macrophages (n=3 per group)**.** P-values were calculated with Student's t-test.

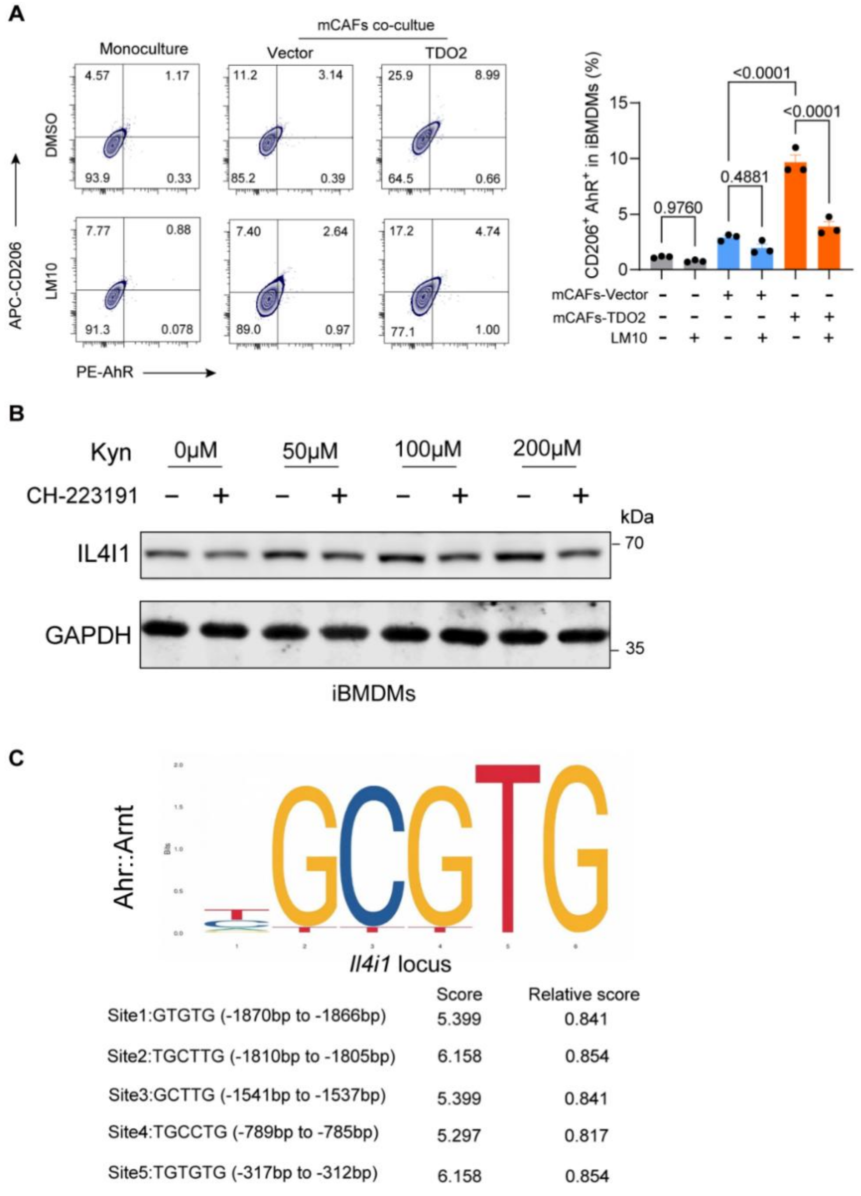

Figure S13. Kynurenine-AhR axis promotes IL4I1 expression in macrophages through direct transcriptional activation. A. Flow cytometry analysis of CD206 and AhR expression in iBMDMs monoculture or co-cultured with mCAFs-Vector/TDO2 cells in the presence or absence of TDO inhibitor LM10 for 72 h (n=3 per group). P-values were calculated with one-way ANOVA test with Tukey's post-hoc test. **B.** Western blotting showing IL4I1 expression in iBMDMs treated with increasing concentrations of kynurenine (Kyn; 0-200 μM) in the presence or absence of AhR antagonist CH-223191 (30 nM). **C.** Predicted AhR-Arnt binding motifs at the *Il4i1* promoter region identified by in silico analysis. Five high-confidence AhR-Arnt binding sites were found upstream of the *Il4i1* transcription start site (TSS), with relative scores ranging from 0.817 to 0.854.

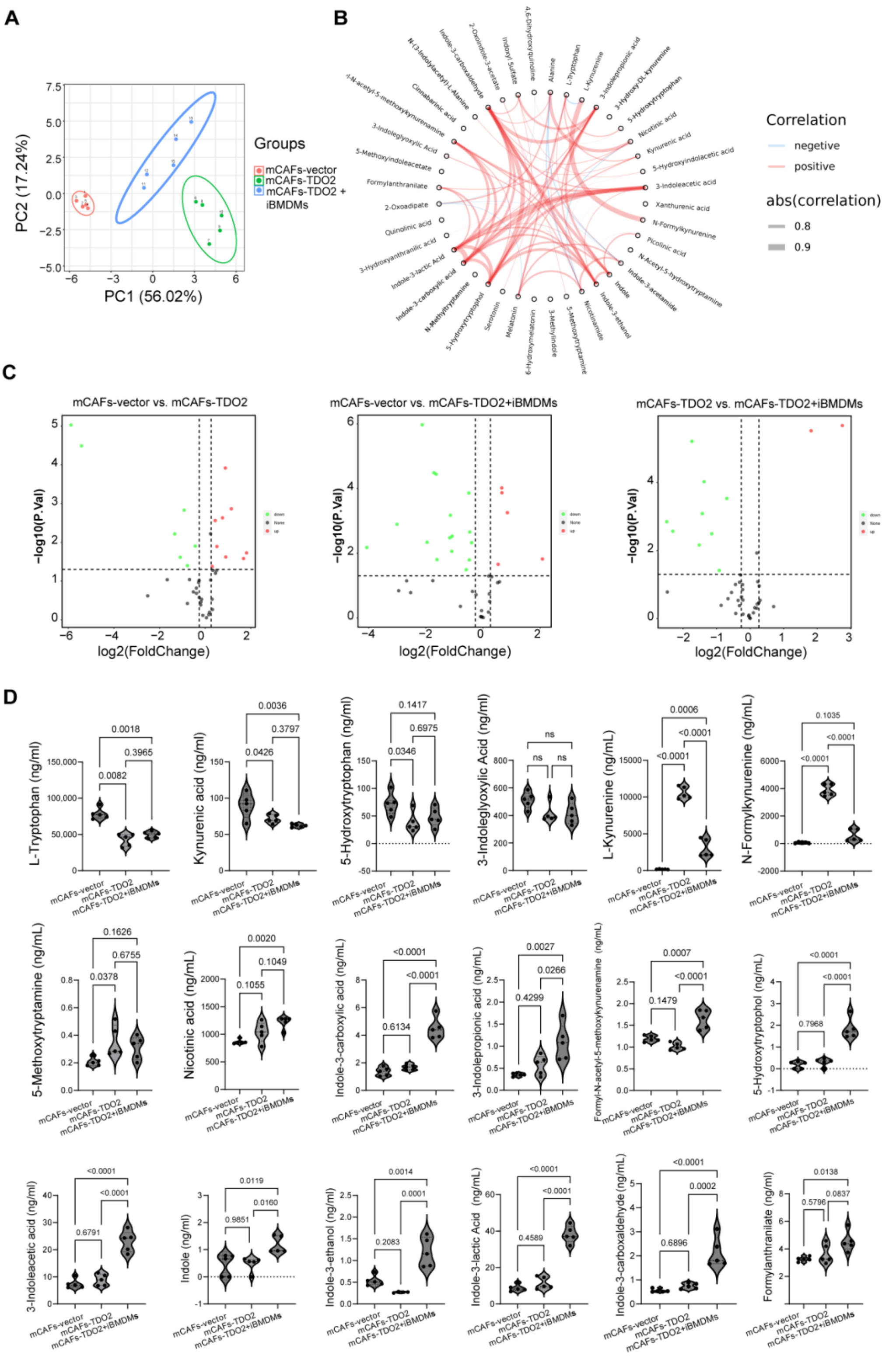

Figure S14. mCAFs-TDO2 and iBMDMs modulate tryptophan metabolism in the tumor microenvironment. A. Principal component analysis (PCA) of Trp-metabolomic profiles revealing distinct clustering among mCAFs-vector, mCAFs-TDO2, and mCAFs-TDO2 co-cultured with iBMDMs. PC1 and PC2 explain 56.02% and 17.24% of total variance, respectively. **B.** Correlation network of significantly altered metabolites in the Tryptophan pathway. **C.** Volcano plots showing differentially regulated metabolites in comparisons between mCAFs-vector vs. mCAFs-TDO2, mCAFs-vector vs. mCAFs-TDO2+iBMDMs, and mCAFs-TDO2 vs. mCAFs-TDO2+iBMDMs. **D.** Violin plots of quantified tryptophan metabolites by LC-MS/MS across indicated conditions (n = 5 per group). P-values were calculated with one-way ANOVA test with Tukey's post-hoc test.

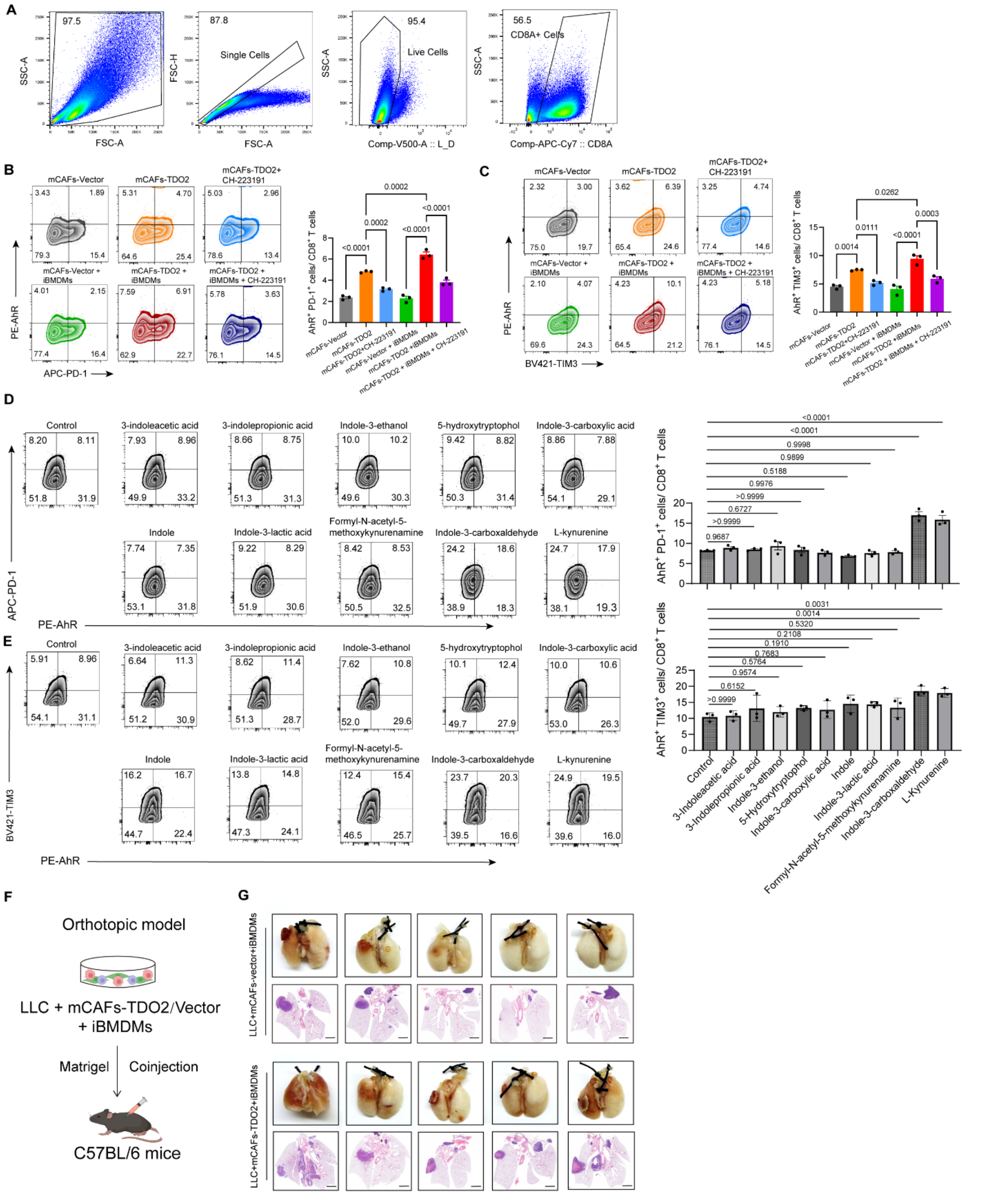

Figure S15. TDO2-expressing CAFs and iBMDMs promote CD8$^+$ T cell exhaustion via AhR activation and tryptophan metabolite signaling. A. Flow cytometry gating strategy for live CD8$^+$ T cells from co-culture in vitro. **B-C.** Representative flow cytometry and quantification showing proportions of AhR$^+$ PD-1$^+$ (**B**) and AhR$^+$ TIM3$^+$ (**C**) in CD8$^+$ T cells co-cultured with mCAFs-vector/TDO2 ± iBMDMs (n=3 per group). The effect is reversed upon treatment with the AhR antagonist CH-223191. P-values were calculated with one-way ANOVA test with Tukey's post-hoc test. **D-E.** CD8$^+$ T cells were treated with indicated tryptophan-derived metabolites for 72 h. Flow cytometry analysis shows increased frequencies of AhR$^+$ PD-1$^+$ (**D**) and AhR$^+$ TIM3$^+$ (**E**) CD8$^+$ T cells (n=3 per group). P-values were calculated with one-way ANOVA test with Tukey's post-hoc test. **F.** Schematic of the orthotopic lung tumor model: LLC cells were co-injected with mCAFs (TDO2 or vector) and iBMDMs in Matrigel into the left lung of C57BL/6 mice. **G.** Representative gross images and H&E staining of lungs at endpoint (n=5 per group). Scale bar = 2mm.

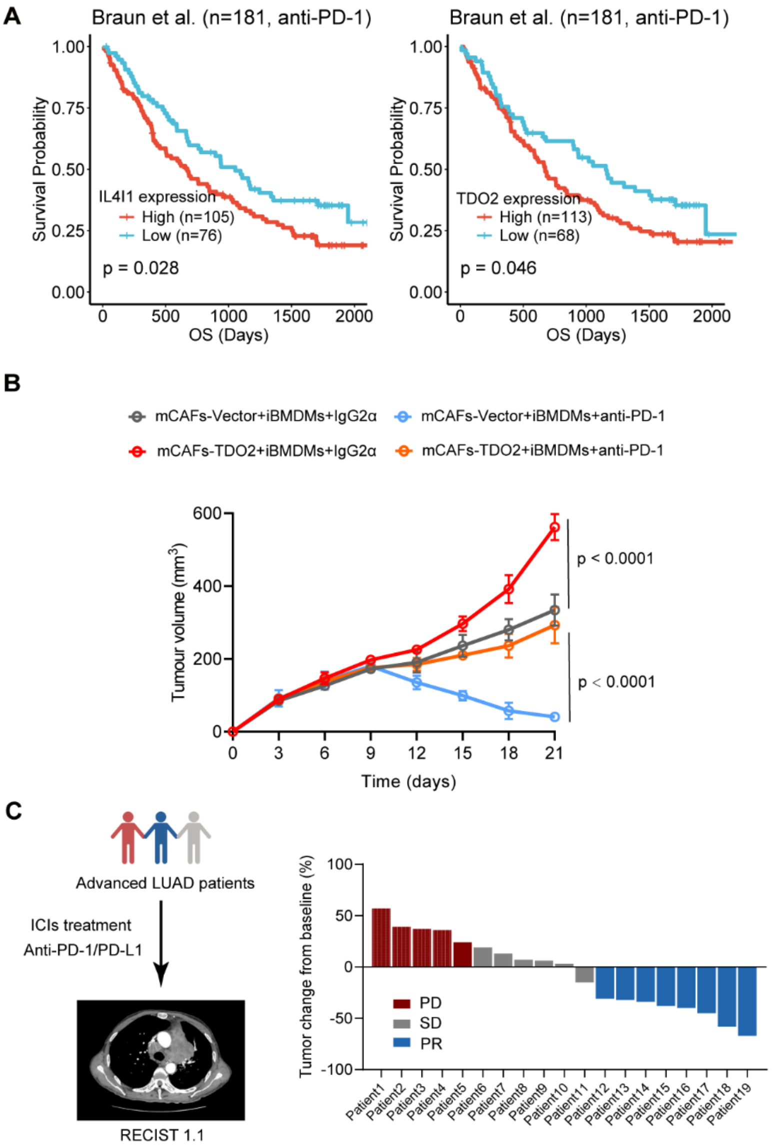

Figure S16. TDO2 and IL4I1 expression correlate with poor anti-PD-1 therapy response and tumor progression. A. Kaplan-Meier survival curves showing overall survival (OS) of anti-PD-1 treated patients (n = 181) from the Braun et al.'s cohort, stratified by IL4I1 (left) or TDO2 (right) expression. **B.** Tumor growth curves in subcutaneous xenograft tumor model treated with anti-PD-1 or isotype control. Mice were co-injected with mCAFs (vector or TDO2) and iBMDMs, followed by treatment with anti-PD-1 or IgG2α control antibody (n = 5 per group). P-values were made by two-way ANOVA with Tukey's correction. **C.** Schematic diagram showing the treatment strategy for advanced LUAD patients, where patients were treated with immune checkpoint inhibitors (ICIs) targeting anti-PD-1/PD-L1. The bars are based on the RECIST 1.1 criteria: Progressive Disease (PD, red, n=5), Stable Disease (SD, gray, n=6), and Partial Response (PR, blue, n=8). Each bar represents the response for an individual LUAD patient (n=19).

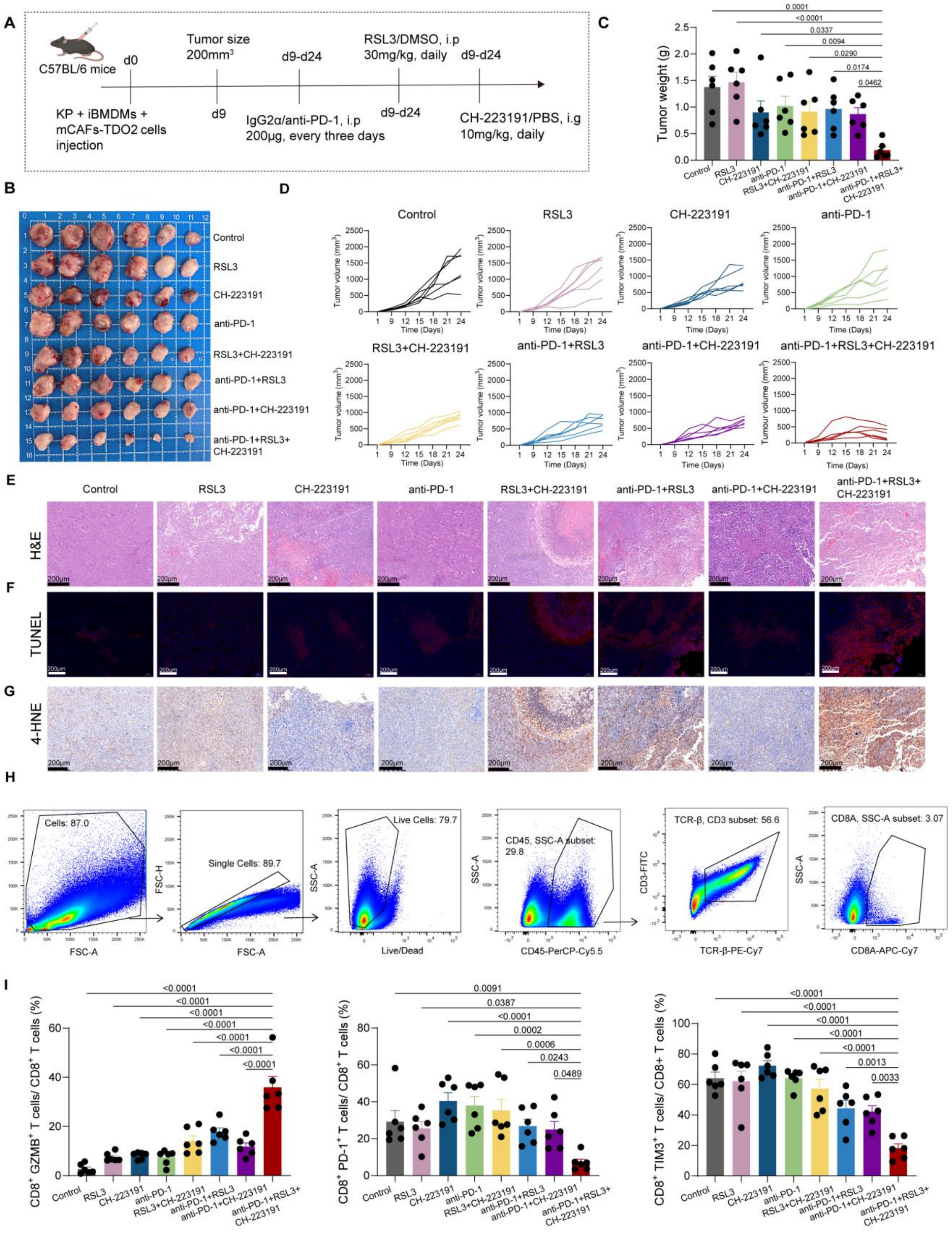

Figure S17. Synergistic efficacy of AhR antagonist, ferroptosis inducer RSL3, and immunotherapy in LUAD treatment. A. Schematic outline showing combination treatment regimen (CH-223191, RSL3, and anti-PD-1) for subcutaneous tumors constructed by mouse KP lung cancer cells, mCAFs-TDO2, and iBMDMs. **B-D.** Gross appearance of the tumor mass (**B**), tumor weight (g) (**C**), and the tumor volume (mm^3) (**D**) were measured and documented for C57BL/6 mice in group (n=6 per group). P-values were calculated with one-way ANOVA test with Tukey's post-hoc test. **E-G.** Representing H&E staining (**E**), TUNEL staining (**F**), and 4-HNE staining (**G**) of tumor tissues under different treatment regimens. **H.** Flow cytometry gating strategy for live $CD8^+$ T cells from treated subcutaneous tumors. **I.** Bar plot showing percentages of $CD8^+$ $GZMB^+$ T cells among $CD8^+$ T cells, $CD8^+$ $PD\text{-}1^+$ T cells among $CD8^+$ T cells, and $CD8^+$ $TIM3^+$ T cells among $CD8^+$ T cells under different treatment regimens (n=6 per group). P-values were calculated with one-way ANOVA test with Tukey's post-hoc test.

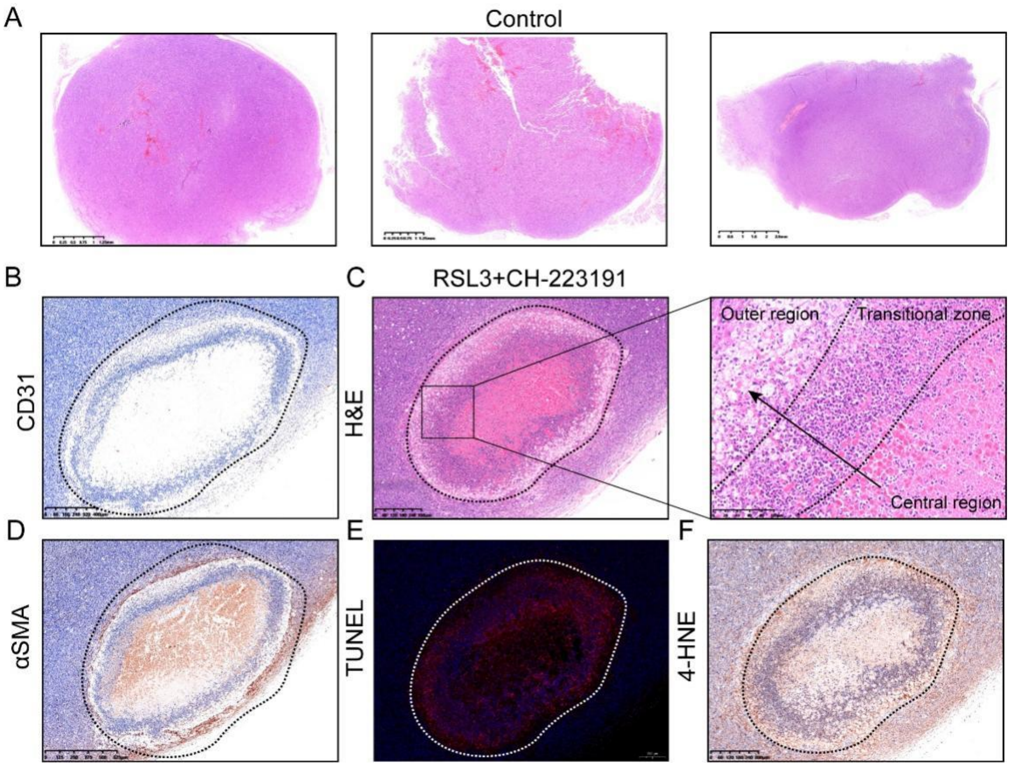

Figure S18. Histopathological characterization of circular structures induced by combined Drug treatment. A. H&E staining of tumor sections from the control group. **B.** CD31 staining showing vascular components. **C.** HE staining of the RSL3+CH-223191 group, highlighting the circular structure with a magnified view of the central, transitional, and outer regions. **D.** αSMA staining indicating fibrosis with hyaline degeneration in the central region. **E.** TUNEL staining showing DNA fragmentation, indicative of cell death. **F.** 4-HNE staining highlighting oxidative stress within the tumor tissue.

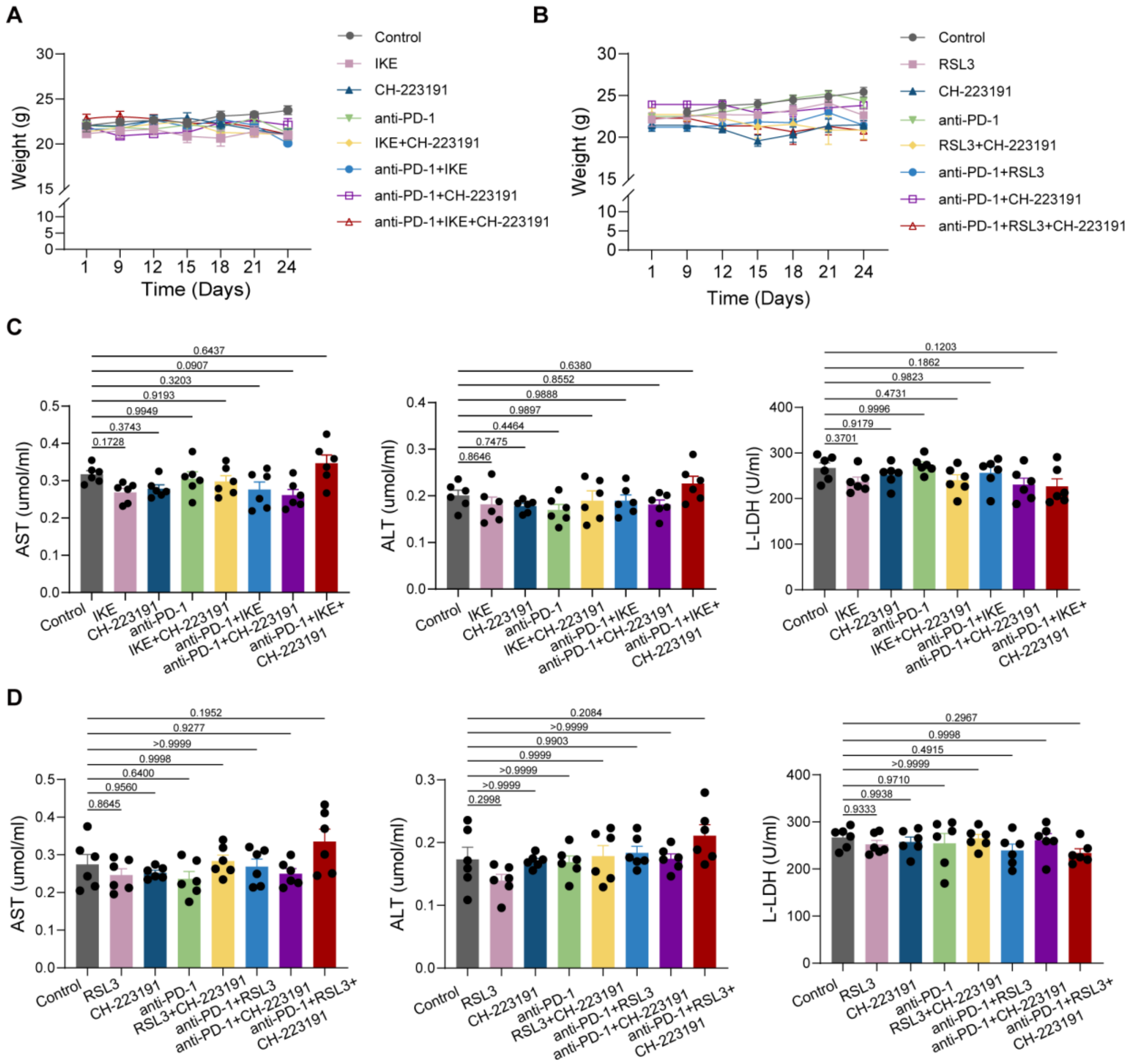

Figure S19. Safety analysis of combination therapy with AhR antagonist, ferroptosis inducer (IKE or RSL3), and anti-PD-1 therapy. A-B. Body weight changes over time in different treatment groups (n = 6 per group). **(A)** CH-223191, IKE, and anti-PD-1. **(B)** CH-223191, RSL3, and anti-PD-1. **C-D.** Serum biochemical assays measuring levels of liver enzymes including aspartate aminotransferase (AST), alanine aminotransferase (ALT), and a cardiotoxicity biomarker lactate dehydrogenase (L-LDH) in peripheral plasma of treated mice (n = 6 per group). **(C)** CH-223191, IKE, and anti-PD-1. **(D)** CH-223191, RSL3, and anti-PD-1. P-values were calculated with one-way ANOVA test with Tukey's post-hoc test.

Supplemental Methods

WGCNA and hub gene screening

Weighted correlation network analysis (WGCNA) is a systems biology method that construct gene co-expression networks for finding modules of highly correlated genes to uncover the correlation between the module eigengene (ME) and external sample traits. In our analysis, we utilized the R package "WGCNA" (v1.71)[1] to identify hub transcriptional features associated with histological subtypes of LUAD. Briefly, we set the parameters as follows: the soft-thresholding power to 5 (scale-free R^2 value of 0.9), cut height to 0.20, and a minimum module size of 30 to identify modules. Input genes were clustered into distinct modules utilizing the dynamic tree cut algorithm, guided by topological overlap matrix (TOM)-based dissimilarity. The ME was derived from the first principal component of each module. We then evaluated the correlation between ME values and histological subtypes using Pearson correlation analysis. Furthermore, we calculated the genetic significance (GS) and module membership (MM) of modules significantly associated with the solid subtype. The inclusion criteria for hub genes were as follows: GS>0.4 and MM>0.7. The STRING (v12.0) database was used to construct a protein-protein interaction (PPI) network. Then, the Cytoscape software (v3.9.1) was applied to visualize the network interactions, and cytoHubba plugins provided top-rank nodes based on maximal Clique Centrality (MCC) scores[2].

Immunological characteristics of TME analysis

We employed a multi-faceted approach to evaluate the diversity in the TME within histological subtypes of LUAD. Initially, we utilized the ESTIMATE tool[3] to calculate immune scores, stromal scores, and tumor purity for each sample. In addition, we harnessed the CIBERSORT[4], EPIC[5], xCell[6], MCP-counter[7], TIMER[8], and quantiseq[7] deconvolution algorithms to elucidate the proportions of immune and stromal cells across histological subtypes. The above algorithms are included in the R package "IOBR" (v0.99.9)[9]. Besides, data on the use of deep learning to identify mappings of TILs from H&E pathological images of TCGA-LUAD were derived from Saltz et al.'s study[10].

Differential expression analysis and functional gene set enrichment analysis

Differential gene analysis of bulk RNA-seq was conducted using DESeq2, which was performed using a generalized linear model with the Wald statistical test, with the assumption that underlying gene expression count data were distributed per a negative binomial distribution with DESeq2. Differentially expressed genes (DEGs) were considered for further analysis with adjusted P-value<0.01 and absolute log2(FoldChange) >1. The adjusted P-value for multiple hypothesis correction was calculated using the Benjamini-Hochberg procedure. The over-representation analyses of Gene Ontology (GO) and Reactome pathways were performed using R package "clusterProfiler" (v4.6.0)[11] and "ReactomePA" (v1.42.0)[12]. Hallmark gene sets were downloaded from the Molecular Signatures Database (MSigDB, https://www.gsea-msigdb.org/gsea/msigdb/). We performed Gene Set Enrichment Analysis (GSEA)[13] to examine significantly enriched pathway.

scRNA-seq processing

The processed data generated from CellRanger were used for downstream analysis based on the R package "Seurat" (v4.3.0)[14]. Only genes expressed at >3 cells and cells with >200 genes detected were kept in the expression matrix. We then removed low-quality cells according to the following criteria: (1) <500 or >100,000 unique molecular identifiers (UMIs); (2) <500 or >10,000 genes; (3) >15% UMIs derived from the mitochondrial genome; (4) >5% UMIs derived from the red-cell genome. After filtering, the function "NormalizeData" was applied to each expression matrix for log transformation, and the function "FindVariableFeatures" was used to select the top 2,000 variable genes. Then, we used the function "ScaleData" to scale and center the expression of 2,000 genes, on which principal component analysis (PCA) was subsequently performed and visualized cells with the uniform manifold

approximation and projection (UMAP) algorithm. R package “harmony” (v0.1.1)[15] was used to eliminate the batch effect and applied “FindNeighbors” and “FindCluster” in Seurat to obtain cell subtypes. The parameter resolution in the Louvain algorithm was set from 0.01 to 1.0 for each cluster and the function “clustree” was used to create a tree containing clusters present at all resolutions.

Cell type annotation

We utilized the function "FindAllMarkers" in Seurat to identify the top differential markers for each identified cluster or sub-cluster. The following parameters were used to identify differentially expressed genes between clusters: min.pct=0.1, logfc.threshold=0.25, pseudocount.use=0.1, only.pos=T. These clusters were firstly identified nine major cell types according to cell type-specific marker genes that previously described in the literature, including epithelial cells (*EPCAM*, *CDH1*, *KRT7*, *KRT18*), T/NK cells (*PTPRC*, *CD3D*, *CD3E*, *NKG7*, *GNLY*), myeloid cells (*CD68*, *CD86*, *CD163*, *S100A8*, *S100A9*, *CSF1R*, *CD14*), B cells (*CD19*, *CD79A*, *MS4A1*), plasma cells (*JCHAIN*, *IGKC*), proliferating immune cells (*MKI67*, *TOP2A*, *PTPRC*), mast cells (*TPSB2*, *KIT*, *CPA3*), endothelial cells (*PECAM1*, *VWF*), and fibroblasts (*COL1A1*, *COL1A2*, *ACTA2*). Cells with expression of double-lineage genes such as PTPRC$^+$ EPCAM$^+$ cells, were excluded to eliminate potential doublet capture bias. The normalized expression of marker genes was scaled and clustered using k-means clustering implemented in the R package “ComplexHeatmap” (v2.15.1)[16] with parameters “column_km=2” and “column_km_repeats=1000”.

Gene signature calculation

Gene signature score could evaluate the strength of a specific cell type or biological process based on the transcriptome. To validate the predictive value of IL4I1$^+$ TAMs and TDO2$^+$ myCAFs in solid predominant LUAD, we characterized the signatures of IL4I1$^+$ TAMs and TDO2$^+$ myCAFs based on the integration from multiple perspectives. The gene sets in the study are listed in **Table S5**. Due to the distinct transcriptomic distributions between bulk RNA-seq and scRNA-seq data, signatures derived from bulk RNA-seq were computed using single-sample gene set enrichment analysis (ssGSEA), while gene signature enrichment in scRNA-seq was performed by the AddModuleScore function of the R package “Seurat”. The normalized matrix was used to establish signature scores following the official guidelines.

Cell-cell interaction analysis

The R package "CellChat" (v1.6.1)[17] was utilized to systematically infer and visualize the number of cell-to-cell interactions between cell clusters. This analysis was conducted using the standard framework outlined. "CellChat" employs a curated ligand-receptor interactions database to infer biologically significant cell-cell communication from scRNA-seq data.

Trajectory inference analysis

To investigate the cellular differentiation from monocytes to IL4I1$^+$ TAMs, we utilized the Monocle2 algorithm[18]. Dimensionality reduction was performed using the “reduceDimension” function with the DDRTree method. Genes associated with pseudotime progression were identified via differentialGeneTest. To examine branch-specific transcriptional changes, we used BEAM analysis. Gene expression dynamics along branches were visualized using “plot_genes_branched_pseudotime”, displaying Loess-smoothed expression curves.

Tissue microarray analysis

The tissue microarray containing 90 cases of LUAD samples was purchased from Outdo Biotech (cat. no. HLugA180Su11). The anti-TDO2 (15880-1-AP, proteintech, 1:300), and anti-IL4I1 (ab222102, Abcam, 1:2000) for signals. Tissue spots that were detached or carbon dust involvement by > 30% were excluded. The image analysis software QuPath[19] was used to calculated H-scores and identify the tumor region or stromal region, and by multiplying the staining intensity by the percentage of stained cells. In

brief, the stain vector was initially estimated. Subsequently, an object classifier was trained using a random trees algorithm with tumor and stromal regions, each color-coded according to their type. Following multiple rounds of cell classification review and correction, the classifier was considered trained when it achieved satisfactory performance, as determined by pathologist assessment indicating the correct classification of the majority of cells.

H&E, IHC staining, and multiplexed IHC staining

Representative 4-μm-thick slides of FFPE tissue were stained for H&E and checked by 2 certified pathologists to determine histological patterns. The clinical datasets were summarized in **Table S2.** IHC staining was performed using anti-TTF1 (BX50074, Biolyx, 1:100), anti-GPX4 (ab125066, Abcam, 1:200), anti-NRF2 (ab76026, Abcam, 1:100), anti-xCT (ab307601, Abcam, 1:500), according to standard procedures. TTF1 staining was used to differentiate primary LUAD from LUSC and metastatic adenocarcinomas from other sites. Multiplexed IHC staining was performed using Novo-light 5-color kit (D110051-50T, WiSee Bio) according to the manufacturer's protocol. Briefly, deparaffinized slides were incubated with various primary antibodies, followed by treated with horseradish peroxidase-conjugated secondary antibody incubation and tyramide signal amplification working solution. Between all steps, the slides were washed with buffer. Finally, nuclei were subsequently visualized with DAPI, and the slides were coverslipped using an anti-fade mounting medium. Multiplexed IHC of human LUAD tissues was performed with the same protocols but different primary antibodies for two panels: Panel A: anti-Pan-Keratin (4545, CST, 1:1000), anti-TDO2 (15880-1-AP, proteintech, 1:300), anti-IL4I1 (ab222102, Abcam, 1:2000), anti-Collagen Type I (67288-1-Ig, proteintech, 1:5000), and anti-CD68 (BX50031, Biolyx, 1:400); Panel B: anti-TDO2 (15880-1-AP, proteintech, 1:300), anti-IL4I1 (Ab222102, Abcam, 1:2000), anti-CD8 (BX50036, Biolyx, 1:300), anti-PD-1 (10377-MM23, Sino Biological, 1:400), and anti-Foxp3 (ab215206, Abcam, 1:100).

Lentiviral vector construction and stable cell line generation

To achieve stable overexpression of TDO2 in mCAFs and IL4I1 in iBMDMs, lentiviral vectors were constructed and transduced. The coding sequences of *Tdo2* and *Il4i1* were cloned into the GV492 lentiviral vector (Ubi-MCS-3FLAG-CBh-gcGFP-IRES-puromycin) by Genechem Co. (Shanghai, China), resulting in the vectors GV492-TDO2 and GV492-IL4I1, respectively. Then, we used lipofectamine 3000 (Invitrogen) to transfect HEK-293T cells with GV492-TDO2/IL4I1 (GV492 empty vector as control) together with psPAX2 and pMD2.G. The supernatant media containing lentivirus were collected by centrifugation and filtration to remove cellular contaminant after 72 h. Further, mCAFs and iBMDMs were infected with these viral particles, and the stably overexpressing cells were screened with puromycin for 5 days. All stable transfected cells were used to analysis within 10 passages.

Conditioned medium collection and cell co-culture system

To obtain conditioned medium for co-culture experiments, mCAFs-vector/TDO2, iBMDMs-vector/IL4I1, and mCAFs-vector/TDO2 co-cultured with iBMDMs (1:1 ratio) were seeded in 100 mm dishes. When the cell confluence reached more than 80%, cells were washed with 1× phosphate-buffered saline (PBS) and incubated with 5 mL serum-free DMEM. The medium was collected after 72 h, then centrifuged and filtered through a 0.22 μm strainer, and diluted with DMEM with 20% FBS at a ratio of 1:1. For co-culture of mCAFs and iBMDMs, mCAFs or iBMDMs were respectively incubated with conditioned medium from iBMDMs-vector/IL4I1 and mCAFs-vector/TDO2. For co-culture of $CD8^+$ T cells and LLC cells, these cells were incubated with conditioned medium from mCAFs-vector/TDO2 and mCAFs-vector/TDO2 co-cultured with iBMDMs. After 72 h, co-cultured cells were collected. RT-qPCR, western blotting, and flow cytometry were used to analyze functional phenotype.

RNA sequencing

mCAFs co-cultured with conditioned medium from iBMDMs-vector/IL4I1 and iBMDMs co-cultured with conditioned medium from mCAFs-vector/TDO2 were harvested, and total RNA was extracted using the TRIzol reagent (Invitrogen) and only high-quality RNA samples [optical density at 260/280 nm (OD260/280) =1.8 to 2.2, OD260/230 ≥ 2.0, RNA integrity number (RIN) ≥ 7.0, 28S:18S ≥ 1.0, > 1μg] were used to construct sequencing library following the manufacturer's protocol. Total RNA sample were then sequenced on Illumina NovaSeq6000 by SHBIO Co. (Shanghai, China). The TPM of each gene were calculated based on the length of the gene and reads count mapped to this gene. DEGs were identified using DESeq2 with adjusted P-value < 0.01 and absolute log2(FoldChange) > 0.585.

Immunoblotting and immunofluorescence staining

For immunoblotting analyses, the whole-cell lysates were in RIPA lysis buffer with protease inhibitor cocktail and phosphatase inhibitor cocktail. The resultant cell lysates were resolved by SDS-polyacrylamide gel electrophoresis and blotted with the antibodies including anti-TDO2 (15880-1-AP, proteintech, 1:800), anti-IL4I1 (ab317248, Abcam, 1:1000), anti-CXCL9 (22355-1-AP, proteintech, 1:1000), anti-IDO1 (13268-1-AP, proteintech, 1:3000), anti-AhR (67785-1-Ig, proteintech, 1:2000), anti-NRF2 (12721, CST, 1:1000), anti-GPX4 (59735, CST, 1:1000), anti-xCT (ab307601, Abcam, 1:1000), anti-KEAP1 (8047, CST, 1:1000), anti-β-actin (20536-1-AP, proteintech, 1:5000), anti-Tubulin (66031-1-Ig, proteintech, 1:20000), and anti-HSP90 (4874, CST, 1:2000).

To assess the subcellular localization of AhR protein, nuclear and cytoplasmic fractions were isolated from iBMDMs using the NE-PER Nuclear and Cytoplasmic Extraction kit (Thermo Scientific, 78833). Briefly, iBMDMs were harvested, washed twice with ice-cold PBS, and resuspended in cytoplasmic extraction buffer containing protease inhibitors. After incubation on ice for 15 minutes, samples were centrifuged at 3,000 rpm for 10 minutes at 4°C. The supernatant (cytoplasmic fraction) was collected, and the pellet (nuclei) was resuspended in nuclear extraction buffer, incubated on ice for 30 minutes with intermittent vortex, followed by centrifugation at 14,000 × g for 20 minutes at 4°C. The resulting supernatant was collected as the nuclear extract. Histone H3 was used as a nuclear control and β-actin was used as a cytosolic control.

For immunofluorescence staining, iBMDMs were seeded on sterile glass coverslips and fixed with 4% paraformaldehyde in PBS for 10 minutes, followed by permeabilization with 0.2% Triton X-100 in PBS for 10 minutes. Cells were then blocked with 5% bovine serum albumin (BSA) in PBS for 30 minutes and incubated with anti-AhR antibody (67785-1-Ig, proteintech, 1:800) for 2h at room temperature. After washing with PBS, cells were incubated with Alexa Fluor 488-conjugated goat anti-mouse IgG secondary antibody (A-11001, Thermo Fisher Scientific) for 1 hour in the dark. Nuclei were counterstained with DAPI-containing mounting medium, and coverslips were mounted and sealed with clear nail polish.

Enzyme-linked immunosorbent assay

To evaluate the expression level of POSTN, CTHRC1, MMP11, IL-1β, CSF1, CCL2, CCL5, CXCL9, and CXCL10 in supernatant of mCAFs-Vector/TDO2, mCAFs-Vector/TDO2 were cultured until they reached 80% confluency and then washed with PBS and cultured in a serum-free medium for 48h. The supernatants were harvested and used for enzyme-linked immunosorbent assay (ELISA) evaluation using mouse Periostin ELISA Kit (MOSF20, R&D Systems), mouse CTHRC1 ELISA Kit (MBS7229601, MyBioSource), mouse MMP11 ELISA Kit (NBP3-06935, Novus), mouse IL-1β ELISA Kit (CSB-E08054m, CUSABIO), mouse M-CSF ELISA Kit (CSB-E04659m, CUSABIO), mouse CCL2 ELISA Kit (CSB-E07430m, CUSABIO), mouse CCL5 ELISA Kit (CSB-E09256m-IS, CUSABIO), mouse CXCL9 ELISA Kit (CSB-EL006252MO, CUSABIO), and mouse CXCL10 ELISA kit (CSB-E08183m-IS, CUSABIO) in accordance with the manufacturer's instructions. To evaluate the expression level of IL4I1 and CXCL9 in supernatant of co-cultured iBMDMs, iBMDMs were cultured

until they reached 80% confluency and then washed with PBS and cultured in a serum-free medium for 48h. The supernatants were harvested and used for ELISA evaluation using mouse L-amino-acid oxidase (IL4I1) ELISA kit (CSB-EL011660MO, CUSABIO) and mouse CXCL9 ELISA kit (CSB-EL006252MO, CUSABIO) in accordance with the manufacturer's instructions. To evaluate plasma levels of IL4I1 in advanced-stage LUAD patients undergoing immunotherapy, we prospectively collected peripheral blood samples from the First Affiliated Hospital of Dalian Medical University and assayed IL4I1 concentrations in plasma using a human IL-4I1/LAO SimpleStep ELISA® kit-Extracellular (ab316898, Abcam) according to manufacturer's instructions. Briefly, the blood samples were collected using EDTA-treated tubes and centrifuged for 15 min at 1000g, and the plasma layer was transferred to separate tubes and stored at −80°C. All samples were analyzed in duplicates, and absorbance was measured at 450 nm using a microplate reader.

Macrophage transwell assays

To evaluate the ability of mCAFs-vector/TDO2 cells to promote chemotaxis and ECM remodeling on macrophages, the chemotaxis and invasion assays were performed. Briefly, for macrophage chemotaxis assay, 4×10^4 iBMDMs were seeded into the upper chamber of an 8.0 μm Transwell insert (3464, Corning) and the same amount of mCAFs-vector/TDO2 cells were placed into the lower chamber as a chemoattractant for 12 h. For invasion assay, the Matrigel matrix (356231, Corning) was diluted 2.5-fold with a serum-free DMEM medium. 100 μL of Matrigel was transferred to the Transwell insert to polymerize at 37°C, 5% CO_2. After polymerization, 4×10^4 iBMDMs were seeded into the upper chamber, and the same amount of mCAFs-vector/TDO2 cells were added to the lower chamber, and iBMDMs were co-cultured for 48 h. After incubation, cells on the upper side of the inserts were gently removed using moistened cotton swabs and the membranes were fixed in ice-cold paraformaldehyde. Cells on the lower surface of the membrane were stained with crystal violet and counted.

Chemicals

CH-223191 (S7711), LM10 (S8368), Staurosporine (S1421), Venetoclax (S8048), Rapamycin (S1039), Olaparib (S1060), Triclabendazole (S4114), Simvastatin (S1792), RSL3 (S8155), Erastin (S7242), and FIN-56 (S8254) were purchased from Selleck.

Cell viability assays

For cell viability assays, 2×10^3 LLC cells were seeded in a 96-well plate and cultured with conditioned medium from mCAFs-vector/TDO2, and mCAFs-vector/TDO2 co-cultured with iBMDMs (1:1 ratio) for 72 h. Then, 100 μL of fresh medium containing the corresponding concentration of various drugs was added to each well for 48 h. The cells were lysed using Cell Counting Kit-8 (HY-K0301, MedChemExpress) according to the manufacturer's instructions. The concentration of the drug resulting in 50% inhibition of cell viability (IC50) was calculated using three-parameter logistic curve fitting. For combination treatments, drug interaction effects were evaluated using the Bliss independence model, and synergy scores were calculated to determine synergistic effects. For colony formation assay, a total of 2×10^3 LLC cells were seeded in 6-well plate and incubated with CH-223191 (30nM) and conditioned medium from mCAFs-vector/TDO2, and mCAFs-vector/TDO2 co-cultured with iBMDMs (1:1 ratio) at 37 °C, 5% CO_2 in humidified incubator for 12 days. Colonies were fixed with 4% paraformaldehyde and stained with crystal violet solution.

C11 BODIPY (581/591) staining

The BODIPY™ 581/591 C11 kit (D3861, Invitrogen) was utilized to evaluate the levels of reactive oxygen species (ROS) detection according to the manufacturer's instructions. Briefly, LLC cells were cultured in conditioned media derived from co-cultures (mCAFs and iBMDMs) for 72 h, followed by treatment with RSL3 for 48 h. After incubation, cells were stained with BODIPY-C11 for 30 min at

37℃, trypsinized, washed with PBS, and analyzed by flow cytometry to measure lipid ROS accumulation.

Determination of intracellular free Fe^{2+}

The FerroOrange intracellular iron measurement kit (F374, Dojindo) was utilized to evaluate the levels of intracellular free Fe^{2+}, in accordance with the manufacturer's instructions.

TUNEL staining

In Situ Cell Death Detection Kit (11684817910, Roche) was utilized to evaluate apoptotic cells, in accordance with the manufacturer's instructions. Briefly, paraffin-embedded tissue sections were subjected to deparaffinization in xylene and a graded ethanol series, followed by rehydration in distilled water. Antigen retrieval was performed by incubating sections with proteinase K solution at 37°C for 30 min. After washing in PBS, membrane permeabilization was conducted using permeabilization buffer at room temperature for 20 min. TdT and dUTP reagents were mixed in a 2:29 ratio and applied to the tissue sections, followed by incubation in a humidified chamber at 37°C for 2 h. After PBS washes, nuclei were counterstained with DAPI for 10 min. Slides were mounted using an anti-fade mounting medium and imaged using a Nikon inverted fluorescence microscope.

References

[1] P. Langfelder, S. Horvath,"Wgcna: An R Package for Weighted Correlation Network Analysis," *BMC bioinformatics* (2008), *9*, 559. https://doi.org/10.1186/1471-2105-9-559.

[2] C. H. Chin, S. H. Chen, H. H. Wu, et al.,"Cytohubba: Identifying Hub Objects and Sub-Networks from Complex Interactome," *BMC systems biology* (2014), *8 Suppl 4*, S11. https://doi.org/10.1186/1752-0509-8-s4-s11.

[3] K. Yoshihara, M. Shahmoradgoli, E. Martínez, et al.,"Inferring Tumour Purity and Stromal and Immune Cell Admixture from Expression Data," *Nature communications* (2013), *4*, 2612. https://doi.org/10.1038/ncomms3612.

[4] B. Chen, M. S. Khodadoust, C. L. Liu, A. M. Newman, A. A. Alizadeh,"Profiling Tumor Infiltrating Immune Cells with Cibersort," *Methods in molecular biology (Clifton, N.J.)* (2018), *1711*, 243-259. https://doi.org/10.1007/978-1-4939-7493-1_12.

[5] J. Racle, D. Gfeller,"Epic: A Tool to Estimate the Proportions of Different Cell Types from Bulk Gene Expression Data," *Methods in molecular biology (Clifton, N.J.)* (2020), *2120*, 233-248. https://doi.org/10.1007/978-1-0716-0327-7_17.

[6] D. Aran, Z. Hu, A. J. Butte,"Xcell: Digitally Portraying the Tissue Cellular Heterogeneity Landscape," *Genome biology* (2017), *18*, 220. https://doi.org/10.1186/s13059-017-1349-1.

[7] E. Becht, N. A. Giraldo, L. Lacroix, et al.,"Estimating The population Abundance of Tissue-Infiltrating Immune and Stromal Cell Populations Using Gene Expression," *Genome biology* (2016), *17*, 218. https://doi.org/10.1186/s13059-016-1070-5.

[8] T. Li, J. Fan, B. Wang, et al.,"Timer: A Web Server for Comprehensive Analysis of Tumor-Infiltrating Immune Cells," *Cancer Res* (2017), *77*, e108-e110. https://doi.org/10.1158/0008-5472.Can-17-0307.

[9] D. Zeng, Z. Ye, R. Shen, et al.,"Iobr: Multi-Omics Immuno-Oncology Biological Research to Decode Tumor Microenvironment and Signatures," *Frontiers in Immunology* (2021), *12*. https://doi.org/10.3389/fimmu.2021.687975.

[10] J. Saltz, R. Gupta, L. Hou, et al.,"Spatial Organization and Molecular Correlation of Tumor-Infiltrating Lymphocytes Using Deep Learning on Pathology Images," *Cell reports* (2018), *23*, 181-193.e187. https://doi.org/10.1016/j.celrep.2018.03.086.

[11] T. Wu, E. Hu, S. Xu, et al.,"Clusterprofiler 4.0: A Universal Enrichment Tool for Interpreting Omics Data," *Innovation (Cambridge (Mass.))* (2021), *2*, 100141. https://doi.org/10.1016/j.xinn.2021.100141.

[12] G. Yu, Q. Y. He,"Reactomepa: An R/Bioconductor Package for Reactome Pathway Analysis and Visualization," *Molecular bioSystems* (2016), *12*, 477-479. https://doi.org/10.1039/c5mb00663e.

[13] A. Subramanian, P. Tamayo, V. K. Mootha, et al.,"Gene Set Enrichment Analysis: A Knowledge-Based Approach for Interpreting Genome-Wide Expression Profiles," *Proceedings of the National Academy of Sciences of the United States of America* (2005), *102*, 15545-15550. https://doi.org/10.1073/pnas.0506580102.

[14] A. Butler, P. Hoffman, P. Smibert, E. Papalexi, R. Satija,"Integrating Single-Cell Transcriptomic Data across Different Conditions, Technologies, and Species," *Nature biotechnology* (2018), *36*, 411-420. https://doi.org/10.1038/nbt.4096.

[15] I. Korsunsky, N. Millard, J. Fan, et al.,"Fast, Sensitive and Accurate Integration of Single-Cell Data with Harmony," *Nat Methods* (2019), *16*, 1289-1296. https://doi.org/10.1038/s41592-019-0619-0.

[16] Z. Gu, R. Eils, M. Schlesner,"Complex Heatmaps Reveal Patterns and Correlations in Multidimensional Genomic Data," *Bioinformatics (Oxford, England)* (2016), *32*, 2847-2849. https://doi.org/10.1093/bioinformatics/btw313.

[17] S. Jin, C. F. Guerrero-Juarez, L. Zhang, et al.,"Inference and Analysis of Cell-Cell Communication Using Cellchat," *Nature communications* (2021), *12*, 1088. https://doi.org/10.1038/s41467-021-21246-9.

[18] X. Qiu, Q. Mao, Y. Tang, et al.,"Reversed Graph Embedding Resolves Complex Single-Cell Trajectories," *Nat Methods* (2017), *14*, 979-982. https://doi.org/10.1038/nmeth.4402.

[19] P. Bankhead, M. B. Loughrey, J. A. Fernández, et al.,"Qupath: Open Source Software for Digital Pathology Image Analysis," *Scientific reports* (2017), *7*, 16878. https://doi.org/10.1038/s41598-017-17204-5.
